# Longitudinal evidence for decreasing statistical learning abilities across childhood

**DOI:** 10.1038/s41467-026-77261-1

**Published:** 2026-09-03

**Authors:** Eszter Tóth-Fáber, Bence C. Farkas, Tímea Tánczos, Dezső Németh, Karolina Janacsek

**Affiliations:** 1 Institute of Cognitive Neuroscience and Psychology, HUN-REN Research Centre for Natural Sciences, Budapest, Hungary; 2 Institute of Psychology, ELTE Eötvös Loránd University, Budapest, Hungary; 3 LNC2, Département d’études Cognitives, École Normale Supérieure, INSERM, PSL Research University, Paris, France; 4 Department of Cognitive and Neuropsychology, Institute of Psychology, University of Szeged, Szeged, Hungary; 5 Centre de Recherche en Neurosciences de Lyon CRNL U1028 UMR5292, INSERM, CNRS, Université Claude Bernard Lyon 1, Bron, France; 6 BML-NAP Research Group, ELTE Eötvös Loránd University & HUN-REN Research Centre for Natural Sciences, Budapest, Hungary; 7 Gran Canaria Cognitive Research Center, Atlántico Medio University, Las Palmas de Gran Canaria, Spain; 8 Centre for Thinking and Learning, Institute for Lifecourse Development, School of Human Sciences, Faculty of Education, Health and Human Sciences, University of Greenwich, London, UK

**Keywords:** Human behaviour, Learning and memory

## Abstract

Statistical learning enables us to identify patterns in our environment and predict future events, thereby supporting a range of motor, cognitive, and linguistic skills. Despite its significance, the developmental trajectory of statistical learning has remained unclear due to a lack of comprehensive longitudinal studies. Cross-sectional research has hinted at enhanced statistical learning in children up to 11-13 years, followed by a decline into adulthood, yet definitive insights require a longitudinal approach. Our study addresses this gap by assessing statistical learning in the same participants at ages 7, 8, 11, and 14. Utilising linear mixed models and latent class analyses, we observe a decline in statistical learning with age, and find that higher executive functions—cognitive functions that underlie higher-order goal-directed behaviour—are associated with this decline. This study provides evidence that statistical learning is more effective in early childhood, offering insights into the developmental trajectory of this fundamental cognitive ability.

## Abstract (web summary)

Through statistical learning, people extract patterns from the environment to predict future events. In a longitudinal study, the authors show that statistical learning declines with age and that higher executive functions are linked to this decline.

## Introduction

Drawing predictions from environmental cues to prepare for the future is a key part of our everyday life, from infancy to old age. Extracting complex regularities embedded in the environment–often referred to as statistical learning–typically happens in an incidental and unsupervised fashion and contributes to the acquisition, storage, and use of implicit motor, cognitive and social behaviours such as skills and habits, which are often considered as manifestations of procedural memory^1–4^. This learning mechanism, often referred to as statistical learning, is crucial for the brain in building predictive models^5,6^. It is remarkably consistent and robust, leading to persistent memory^7–9^.

To understand the neuroplasticity that underpins predictive processes, it is essential to examine the development of statistical learning by observing age-related changes in the same individual over time. However, such longitudinal research on statistical learning is scarce, with most conclusions drawn from cross-sectional data. These cross-sectional studies suggest changes in statistical learning across development in most cases, with the exact trajectories depending on modality and types of the acquired regularity^10–17^. Although cross-sectional studies with between-person comparisons help characterise age differences in cognitive functions, they cannot equate to the age-related changes measured by longitudinal studies involving within-person comparisons. Single cross-sectional designs are confounded by cohort and period effects; hence, they are not well-suited to provide an accurate picture of an individual’s developmental trajectory^18^. Longitudinal studies better describe how certain cognitive functions change over the lifespan.

To the best of our knowledge, only two longitudinal studies involved the development of statistical learning. Kidd et al.^19^ assessed children in their first two years of primary education four times, with 6-month-long intervals. However, their study focused only on the relationship between statistical learning and language proficiency, leaving the development of statistical learning itself unexplored. In another study, François et al.^20^ examined the effect of musical training on auditory statistical learning in childhood, and the control group showed no change in auditory statistical learning between ages 8 and 10. As the main focus of these studies was not the development of statistical learning itself and their scope was limited to a relatively short testing period, drawing conclusions on the developmental trajectory of statistical learning remains challenging. Here, we address these gaps by directly testing the changes in statistical learning performance from childhood to adolescence using a longitudinal design with four testing sessions spanning seven years.

The development of statistical learning can be better understood in relation to broader cognitive development and other cognitive functions, such as top-down cognitive processes^21^. These processes involve internal models and executive functions, which are crucial for the flexible control of behaviour^22–25^. Executive functions (EF) are a set of higher-order cognitive skills that are responsible for attention, thought, and action, including working memory, cognitive flexibility, and inhibition^22,26^. These functions rely on the prefrontal cortex and continue to mature until young adulthood^27^.

The ‘competition model’ suggests that the superior statistical learning found in children under the age of 12 may stem from a relatively lower reliance on top-down processes such as internal models and EF^11,28,29^. Children may be more attuned to raw environmental probabilities as their internal models–structured mental representations of the world, formed through experience–are less developed. During adolescence, as individuals gain more experience, their internal models become increasingly refined. This maturation enables more complex interpretations of events but may come at the cost of reduced sensitivity to statistical regularities in the environment. Thus, there is a developmental shift toward greater reliance on internal models at the expense of detecting raw probabilities.

Similarly, Wu et al.^30^ distinguish between open-minded input-driven learning and knowledge-driven learning in their theoretical framework. They argue that in childhood, learning tends to be input-driven and open-minded due to limited prior experience. This form of learning is well-suited for detecting raw environmental regularities, a core component of statistical learning. As individuals mature and accumulate knowledge, they tend to rely more heavily on prior knowledge, including schemas, internal models, routines, and assumptions^30–32^. Although the present study does not directly test prior knowledge, these frameworks highlight the importance of investigating the development of statistical learning and its relation to top-down cognitive processes.

Here, we utilised the benefits of longitudinal designs to assess participants at four time points: at 7, 8, 11, and 14 years of age. This approach enabled us to follow the development of statistical learning from childhood to adolescence, a period where cross-sectional studies have suggested significant changes^11,12,14^. To measure statistical learning, we employed the well-established Alternating Serial Reaction Time (ASRT) task^33^, which has also been used in previous large-scale cross-sectional studies^11,12^. This task choice allows for direct comparison between our longitudinal results and previous cross-sectional findings. Furthermore, the ASRT task provides an online measure of statistical learning, capturing the learning process as it unfolds. Online measures are thought to be more sensitive and reliable indices of learning than offline measures, which may be confounded by individual differences in memory and metacognitive abilities^34,35^. To reveal the developmental trajectory of statistical learning, we employed linear mixed models with a random effects structure to account for participant- and age-specific differences in baseline performance, providing a more accurate estimation of age effects in statistical learning. These models indicated a decreasing trend in statistical learning across development. Additionally, to examine the potential influence of broader cognitive functions, we also assessed EFs using a battery of well-established and widely used tasks suitable for children aged 7 to 14^36–40^. These included complex, verbal and visuo-spatial working memory tasks to evaluate the updating component of EF, along with a verbal fluency task designed to tap into all EF components^41^. We then used a latent class analysis to identify subgroups of participants with distinct developmental trajectories of statistical learning and tested whether EF predicted subgroup membership. We found that interindividual differences in these trajectories were associated with EF performance. Specifically, higher EF at age 14 was linked to a decreasing statistical learning trajectory.

## Results

**Fig. 1:**
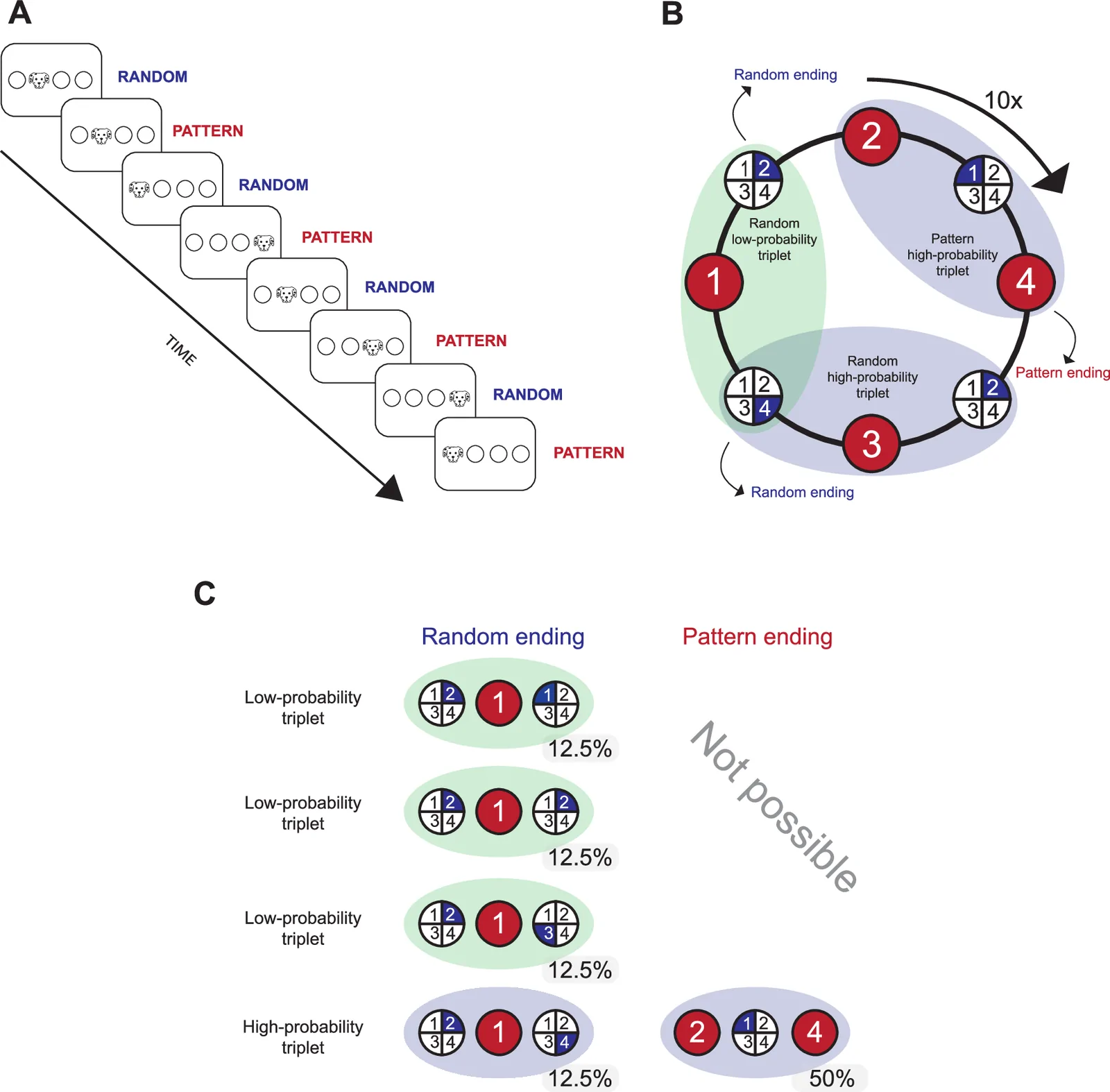
The Alternating Serial Reaction Time (ASRT) task. **A** In the ASRT task, participants had to press keys corresponding to the location of the target stimulus (dog’s head, the stimulus is shown here as a schematic redrawing). The presentation of stimuli followed an eight-element sequence, with pattern and random trials alternating with each other. **B** The red circles represent the pattern trials (numbers indicating the location of the stimulus on the screen from left to right) and the white circles represent random trials, with the dark blue shaded number indicating the location where the stimulus appeared by chance in that trial. The black arrow shows the order in which the stimuli would appear in this example. The alternating sequence of pattern and random trials makes some runs of three consecutive trials (called triplets) occur with higher probability than others, referred to as high-probability and low-probability triplets, respectively. High-probability triplets can occur in two ways: either as the third element of the triplet being part of the pattern (with light purple shading) or as the third element of the triplet being selected randomly (with light purple shading). Low-probability triplets can only occur with the third element of the triplet being random (with light green shading). Note that each trial serves as the third, middle and first element of a triplet in a moving window manner (for details, see the main text), but here, we only categorise a few trials for illustrative purposes. **C** The alternating sequence structure resulted in higher occurrences of some triplets (high-probability triplets, 62.5% of all trials) than others (low-probability triplets, 37.5% of all trials). Statistical learning can be measured by performance improvements on trials that serve as the third element of a high-probability triplet versus those that serve as the third element of a low-probability triplet.

The final sample consisted of 97 participants (42 boys, 55 girls, age at first assessment: *M* = 6.81 years, *SD* = 0.55 years). Our longitudinal study included four assessments conducted at ages 7 (1st grade), 8 (2nd grade), 11 (5th grade) and 14 (8th grade). At each assessment session, statistical learning was measured with the Alternating Serial Reaction Time (ASRT) task (Fig. 1). Statistical learning was indexed by the difference in performance between high- and low-probability triplets. As previous studies have demonstrated, successful learning is reflected by faster and more accurate responses to high-probability than to low-probability triplets, indicating sensitivity to the underlying regularities acquired incidentally during the task. To measure executive functions, participants also completed the Counting span, Digit span, Backward digit span, Corsi block tapping, and Fluency tasks (for details, see Methods).

### What are the developmental trajectories of statistical learning?

We investigated the developmental trajectories of statistical learning by using block-wise median RT as the outcome variable in a linear mixed model; analyses on accuracy scores are reported in the Supplementary Information (Supplementary Note S1). The RT model included Triplet type (high- vs. low-probability), Block (Block 1-20), and Age (7, 8, 11 or 14), along with all their higher-order interactions as fixed effects, and uncorrelated by-participant intercepts and slopes for Block, Age, and their interaction as random effects. The Triplet type factor indicates differences in statistical learning, while main effects and interactions without the Triplet type factor reflect differences in overall RTs. Crucially, the fixed-effects estimates tested our main hypotheses regarding the overall developmental trajectories in statistical learning, while the random-effects estimates accounted for differences in overall RTs and RT differences attributable to practice (within-block) and age across participants. Henceforth, we use the term *learning score contrast* to refer to performance differences between high- and low-probability triplets estimated from the LMM using the estimated marginal means approach^42^, and the term *average learning score* to refer to performance differences between high- and low-probability triplets calculated from raw data by averaging.

Model results are summarised in Tables 1 and 2. We first summarise the random effects (see also Supplementary Fig. S2A). The adjusted ICC of the model was .43, indicating moderate clustering in the data. Variance estimates at each level revealed clustering in both intercepts, reflecting interindividual variability in overall RTs (*SD* = 72.78), and slopes, reflecting interindividual variability in the amount of speed-up within blocks (*SD* = 4.16) and at different ages (*SD*_*Age7*_ = 112.95, *SD*_*Age8*_ = 59.33, *SD*_*Age11*_ = 45.45), with very little variance of the Age × Block interaction (*SD*_*Block × Age7*_ = 7.51, *SD*_*Block × Age8*_ = 3.63, *SD*_*Block × Age11*_ = 2.78). This indicates that most interindividual variability came from differences in overall RT levels at different ages, and the amount of speed-up within sessions, with remarkable homogeneity in the trajectories across ages. Residual variance was *SD* = 83.84.

**Table 1:** Type III test of effects of the linear mixed model, predicting block-wise median reaction times

Type III test of effects	*F*	*df*	*p*
Age	140.91	3, 99.49	<.001*
Block	282.67	1, 88.58	<.001*
Triplet type	126.18	1, 11660.97	<.001*
Age × Block	17.43	3, 101.48	<.001*
Age × Triplet type	4.19	3, 11660.96	.006*
Block × Triplet type	7.68	1, 11661.03	.006*
Age × Block × Triplet type	0.57	3, 11661.02	.637

Significant terms are marked with an asterisk. All *p* values are two-sided and unadjusted for multiple comparisons.

**Table 2:** Fixed and random effects of the linear mixed model, predicting block-wise median reaction times

Fixed effects	*b*	*SE b*	*95% CI*	*t*	*df*	*p*
(Intercept)	576.10	7.71	560.80–591.40	74.76	94.18	<.001*
Age [7 y]	213.23	12.09	189.26–237.24	17.63	101.31	<.001*
Age [8 y]	23.23	7.73	7.85–38.60	3.01	79.15	.004*
Age [11 y]	−67.45	6.01	−79.54 to −55.47	−11.23	70.07	<.001*
Block	−7.76	0.46	−8.67 to −6.84	−16.81	88.58	<.001*
Triplet type [Low]	8.63	0.77	7.12–10.13	11.23	11660.97	<.001*
Age [7 y] × Block	−4.53	0.83	−6.18 to −2.89	−5.48	102.23	<.001*
Age [8 y] × Block	−1.93	0.53	−2.99 to −0.88	−3.64	83.31	<.001*
Age [11 y] × Block	1.46	0.43	0.60–2.33	3.37	77.35	.001*
Age [7 y] × Triplet type [Low]	3.34	1.28	0.83–5.84	2.61	11660.85	.009*
Age [8 y] × Triplet type [Low]	1.39	1.43	−1.41–4.18	0.97	11660.93	.331
Age [11 y] × Triplet type [Low]	−1.46	1.38	−4.16–1.24	−1.06	11661.13	.289
Block × Triplet [Low]	0.37	0.13	0.11–0.63	2.77	11661.03	.006*
Age [7 y] × Block × Triplet type [Low]	−0.24	0.22	−0.67–0.20	−1.07	11660.87	.286
Age [8 y] × Block × Triplet type [Low]	0.23	0.25	−0.25–0.72	0.94	11660.90	.348
Age [11 y] × Block × Triplet type [Low]	0.09	0.24	−0.38–0.56	0.37	11661.13	.712
**Random Effects**						
σ^2^	7028.39 (83.84)					
τ_00 Participant_	5297.33 (72.78)					
τ_11 Block_	17.30 (4.16)					
τ_11 Age[7y]_	12757.80 (112.95)					
τ_11 Age[8y]_	3519.55 (59.33)					
τ_11 Age[11y]_	2065.67 (45.45)					
τ_11 Block × Age[7y]_	56.47 (7.51)					
τ_11 Block × Age[8y]_	13.20 (3.63)					
τ_11 Block × Age[11y]_	7.72 (2.78)					
ICC	0.43					
N _Participant_	97					
Observations	12310					
Marginal R^2^ / Conditional R^2^	0.668 / 0.811					

The table shows regression coefficients of fixed effects and summary information about the random effects. Random effects are presented as variance and SD in brackets. The marginal R-squared considers only the variance of the fixed effects, while the conditional R-squared takes both the fixed and random effects into account (based on Nakagawa et al.^76^). Degrees of freedom are based on Satterthwaite’s approximation. Significant terms are marked with an asterisk. All *p* values are two-sided and unadjusted for multiple comparisons. Terms in brackets indicate the level of the factor that is contrasted against the reference level (High triplets for Triplet type and Age 14 for Age). Because we were interested in lower order effects too, both Age and Triplet type were effects coded, therefore lower order interactions and main effects are to be interpreted as deviations from the grand mean value of the dataset (at age 10 in our case, even though this value was not practically observed, and at the average of high- and low-triplets).

Model equation in *lmer* syntax: RT ~ Age*Block*Triplet type + (Block*Age || Participant).

Regarding the fixed effects, the model revealed a significant main effect of Block (*F*_(1, 88.58)_ = 282.67, *p* < .001), indicating a decrease in overall RTs throughout the task (*b* = −7.76, 95% CI = [−8.67, −6.84]). The main effect of Age was also significant (*F*_(3, 99.49)_ = 140.91, *p* < .001), indicating decreasing overall RTs with development (Age 7: 789 ms, 95% CI = [761, 818]; Age 8: 599 ms, 95% CI = [577, 622]; Age 11: 509 ms, 95% CI = [489, 528]; Age 14: 407 ms, 95% CI = [376, 438]; with each pairwise comparison being significant at *p* < .001). The Age × Block interaction was also significant (*F*_(3, 101.48)_ = 17.43, *p* < .001), with post hoc estimated marginal trends revealing a progressively less steep slope for Block with development (Age 7: *b* = −12.29, 95% CI = [−14.16, −10.42]; Age 8: *b* = −9.69, 95% CI = [−11.13, −8.25]; Age 11: *b* = −6.29, 95% CI = [−7.55, −5.03]; Age 14: *b* = −2.75, 95% CI = [−4.76, −0.74]; all pairwise comparisons *p*s < .062). While one could argue that generally slower RTs at younger ages could provide more room to improve, a cross-sectional ASRT study, using various control analyses, showed that the greater within-session improvement in younger age groups could not be explained by generally slower RTs^12^.

**Fig. 2:**
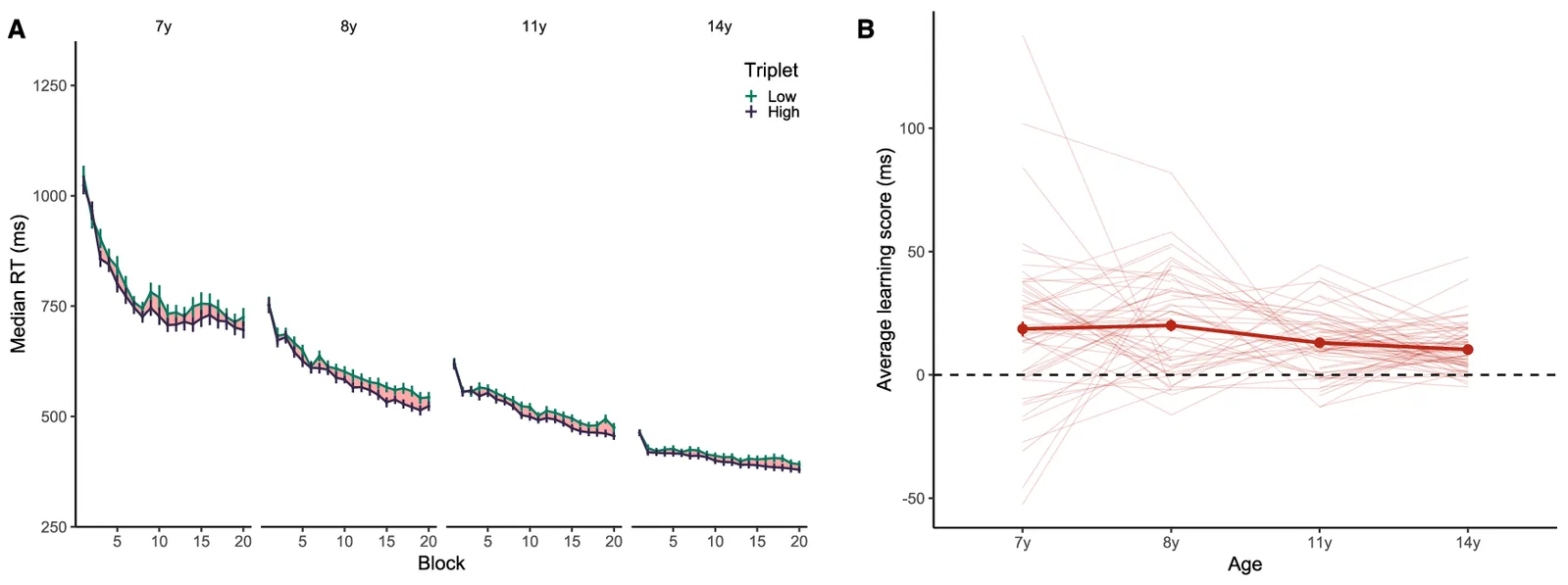
Trajectories of statistical learning. **A)** Average block-wise median RTs as a function of triplet type (green for low-probability, indigo for high-probability) for the 4 ages tested. Differences in RTs between the more (high-probability) and less predictable (low-probability) stimuli indicate statistical learning and are highlighted by light red shading. Visually, statistical learning is marked by the distance between the green and indigo lines. Linear mixed models revealed that the amount of statistical learning decreased with development. **B)** Whole task average learning scores for the 4 ages. Learning scores indicate the overall amount of statistical learning and are calculated by subtracting RTs between the high- and low-probability triplets (corresponding with the light red shading of panel A). Individual learning trajectories are shown by the light red lines. In all panels, points indicate the mean, error bars indicate 1 within-subjects standard error of the mean calculated by the method of Morey^86^ and data are shown for N = 97 participants.

The model also revealed a significant main effect of Triplet type (*F*_(1, 11660.97)_ = 126.18, *p* < .001), with faster RTs for high-probability triplets than for low-probability triplets (high: 568 ms, 95% CI = [552, 583]; low: 585 ms, 95% CI = [569, 600]), indicating statistical learning. There was a significant Block × Triplet type interaction (*F*_(1,11661.03)_ = 7.68, *p* = .006), revealing increasingly faster RTs for high-probability triplets (*b* = −8.12, 95% CI = [−9.08, −7.17]) than low-probability triplets (*b* = −7.39, 95% CI = [−8.34, −6.43]), indicating increasing statistical learning as the task progressed. There was a significant Age × Triplet type interaction (*F*_(3,11660.96)_ = 4.19, *p* = .006). Learning score contrasts indicated decreasing statistical learning with development (Fig. 2A; Age 7: 23.90 ms, 95% CI = [18.25, 29.60]; Age 8: 20.00 ms, 95% CI = [13.36, 26.70]; Age 11: 14.30 ms, 95% CI = [7.97, 20.70]; Age 14: 10.70 ms, 95% CI = [5.42, 16.00]); age 14 learning score contrasts were significantly lower than age 7 (*p* < .001) and 8 (*p* = .032), and age 11 learning score contrasts were significantly lower than age 7 (*p* = .027). This decreasing pattern was also evident when calculating average learning scores from the raw data instead of model estimates, with average learning scores being about twice as high at age 7 (*M* = 18.62 ms) as at age 14 (*M* = 10.27 ms) (Fig. 2B). Of the 83 participants with data at both ages, 48 ( ~ 58%) had higher learning scores at age 7 than at age 14, and this corresponded with a medium-sized effect (Cohen’s d = 0.228) and a statistically significant difference in a paired t-test (*t*_(82)_ = 2.08, *p* = .040). The lack of a significant three-way Triplet Type × Age × Block interaction indicates that, despite the decrease in overall levels of statistical learning with development, the dynamics of learning during the task remained relatively constant. Finally, a contrast which compared changes in high- and low-probability triplets between successive timepoints revealed that the amount of developmental changes was comparable between triplet types (7y–8 y: *p* = .381; 8y–11 y: *p* = .225; 11y–14 y: *p* = .394), indicating that the Age × Triplet interaction was driven by an overall reduction in performance *difference* between triplets (which is commonly taken as the relevant SL measure in the ASRT) rather than a selective age effect on one triplet type. A Bayesian re-estimation of our primary LMM yielded virtually identical parameter estimates and the same substantive conclusions, with inference based on 95% credible intervals fully consistent with the frequentist results (Supplementary Fig. S3).

### Are there identifiable subgroups with different developmental trajectories of statistical learning?

To identify potential subgroups of participants with different developmental trajectories of statistical learning, we employed Latent Class Analysis (LCA), specifically using the growth mixture model approach (for details, see Statistical analysis section).

**Fig. 3:**
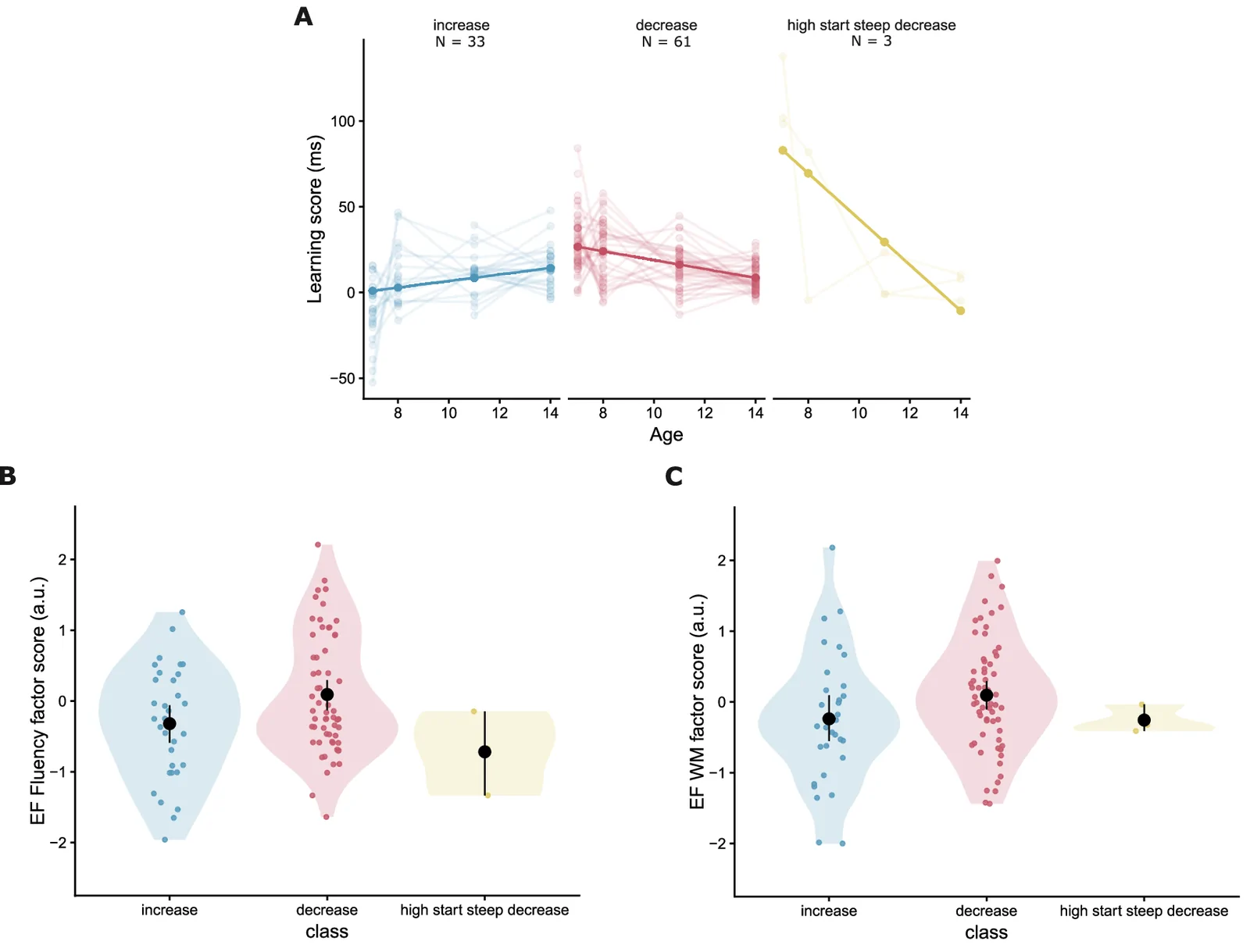
Identification of subgroups, based on developmental trajectories in statistical learning. **A)** Results of the selected 3-class latent class analysis model. This model identified 3 classes of participants, which on the basis of their developmental trajectories were labelled ‘increase’, ‘decrease’, and ‘high start steep decrease’, with most participants being characterised by a decreasing pattern. Estimated means within each class are indicated by thick lines and points, whereas actual individual trajectories of participants within each class are indicated by slightly transparent lines and points. **B)** Fluency executive function factor scores in the various subgroups. **C)** Working memory executive function factor scores in the various subgroups. In panels B) and C), points are individual participants, violin plots indicate the smoothed distribution, thick black points indicate the mean, black error bars indicate 1 standard error. In all panels, data are shown for N = 97 participants.

Although the two-class model resulted in the lowest SABIC (SABIC = 2689.649), it comprised two very unbalanced classes (class 1 = 3.1%; class 2 = 96.9%) (Table 3). The three-class model had the second lowest SABIC (SABIC = 2691.337) and produced more balanced classes (class 1 = 62.9%; class 2 = 3.1%; class 3 = 34.0%). Mean posterior classification probabilities were also adequate in each class (*p*_(class1)_ = .85; *p*_(class2)_ = .99; *p*_(class3)_ = .80). We therefore selected the three-class model as our primary model of interest (Fig. 3A). Results of the 1 and 2-class models are reported in Supplementary Note S2.

**Table 3:** Results of the Latent Class Mixed Models

*Model*	*AIC*	*SABIC*	*Entropy*	*class1 (N, %)*	*class2 (N, %)*	*class3 (N, %)*
1-class	2697.972	2694.475	1.00	97 (100%)	-	-
2-class	2694.895*	2689.649*	0.95	3 (3.1%)	94 (96.9%)	-
3-class*	2698.332	2691.337	0.65	61 (62.9%)	3 (3.1%)	33 (34.0%)

The AIC and SABIC are shown as goodness-of-fit indices, the entropy characterises the classification performance, with higher values indicating that individuals can be classified with more confidence. Sizes of classes (in absolute number, and relative percentage of participants) are also reported. Asterisks indicate which model is the best fitting according to the fit index. The solution that provided the best trade-off between goodness of fit, class balance, and classification performance is the 3-class model. AIC = Akaike information criterion; SABIC = Sample size adjusted Bayesian information criterion.

The first class of this model included the largest number of participants (*N* = 61). It was characterised by a relatively high intercept (*b* = 26.63 ± 3.18 SE, *p* < .001) and a statistically significant decreasing slope of average learning scores with development (*b* = −2.59 ± 0.64 SE, *p* < .001) leading to a predicted difference of −18.11 ms (0.89 SDs) between the first and last timepoint. Hence this category was labelled ‘decrease’. The second class had the fewest participants (*N* = 3). It was characterised by an extremely high intercept (*b* = 82.91 ± 8.25 SE, *p* < .001) and a substantial statistically significant decrease in average learning scores with development (*b* = −13.37 ± 1.88 SE, *p* < .001), leading to a predicted difference of −93.57 ms (4.58 SDs) between the first and last timepoint. Hence it was labelled ‘high start steep decrease’. The third class included about one-third of participants (*N* = 33). It was characterised by an intercept close to 0 (*b* = 0.95 ± 5.11 SE, *p* = .852) and a slight, but statistically significant increase in average learning scores with development (*b* = 1.91 ± 0.94 SE, *p* = .043), leading to a predicted difference of +13.34 ms (0.65 SDs) between the first and last timepoint. Hence it was labelled ‘increase’.

In light of the modest entropy, we thought the robustness and validity of the classes should be more thoroughly investigated. To assess this, we fit a range of longitudinal clustering models spanning distinct algorithmic families^43^, including regression-based k-means (LMKM), mixed-model–based k-means (GCKM), group-based trajectory modelling (GBTM), and growth mixture modelling with random intercept only (GMM1), and compared them to our original growth mixture model including random slopes (GMM2). Across all methods, SABIC favoured the 3-class solution (Supplementary Note S3), with qualitatively similar trajectory patterns as our original model—a decreasing trajectory with the highest number of participants, an increasing trajectory with a smaller number of participants, and a high-initial-then-decrease trajectory with the smallest number of participants—indicating strong structural replicability, with GBTM, GMM1, and our original GMM2 model recovering the exact same classification (Supplementary Fig. S6). Agreement between methods was moderate to perfect (Adjusted Rand Index ≈ 0.48–1.00; Normalised Mutual Information ≈ 0.55–1.00). Differences across methods primarily reflected the treatment of borderline trajectories rather than disagreement about the underlying trajectory structures.

The LCA results align well with our LMM findings, indicating that most participants exhibited a decreasing trend in statistical learning scores with development, while a subset showed a modest increase. We next aimed to understand the factors influencing whether a given participant showed a decreasing or an increasing trend in their statistical learning, thus predicting class membership.

### Do executive functions predict subgroup membership?

We investigated whether EF ability predicted the developmental trajectory subgroup of participants. To this end, we performed a logistic regression, with class membership weighted by the posterior probabilities as outcomes, and Fluency and Working memory (WM) latent EF factor scores as predictors (for the calculation of the EF factor scores, see Supplementary Note S4). The reference class was set to ‘increase’. Due to the very low number of participants, we omitted the ‘high start steep decrease’ class from this analysis.

The logistic regression yielded a significant effect, with a joint LRT suggesting that the inclusion of EF scores improved model fit (χ2(2) = 8.55, *p* < .001). When examining the two EF factors individually in the model, as Fluency scores increased, participants were less likely to be classified in the ‘increase’ trajectory subgroup and more likely to be classified in the ‘decrease’ trajectory subgroup (OR = 2.03, 95% CI = [1.19, 3.83], *p* = .019). Additionally, as WM scores increased, participants were less likely to be classified in the ‘increase’ trajectory subgroup and more likely to be classified in the ‘decrease’ trajectory subgroup, but this effect did not reach significance (OR = 1.58, 95% CI = [0.93, 2.80], *p* = .103, Table 4). These results suggest that, overall, better EF ability (especially as measured by the Fluency factor) at age 14 is related to a decreasing trajectory of statistical learning between ages 7 and 14 (Fig. 3BC; note that the factor scores for the ‘high start steep decrease’ subgroup are presented for completeness only, this subgroup was not included in the analysis as described above).

**Table 4:** Results of the probability-weighted logistic regression predicting class membership (increase vs decrease)

*Predictor*	*B (SE)*	*t*	*p*	*OR*	*95% CI for OR*
Intercept	0.75 (0.24)	3.12	.002*	2.12	[1.34, 3.46]
EF WM	0.46 (0.28)	1.65	.103	1.58	[0.93, 2.80]
EF Fluency	0.71 (0.30)	2.40	.019*	2.03	[1.19, 3.83]

Residual deviance = 91.29 (df = 91), null deviance = 99.84 (df = 93). Dispersion parameter = 0.86. Coefficients are reported on the log-odds scale, and as Odds Ratios (OR). All *p* values are two-sided and unadjusted for multiple comparisons. Significant terms are marked with an asterisk.

We also assessed the robustness of this finding by extracting classes from the 3-class solutions from each of our sensitivity models, and estimated multinomial or binary logistic regression models relating WM and Fluency factors to the probability of belonging to the ‘increase’ versus other classes for each clustering algorithm. Joint likelihood ratio tests indicated that the combined contribution of EF measures significantly improved model fit in each analysis. Across all methods, higher executive function was associated with increased odds of belonging to the ‘decrease’ over the ‘increase’ trajectory, although the relative strength of the Fluency and WM scores was model dependent (Supplementary Figs. S9 and S10).

To further examine the relationship between statistical learning and EFs, we correlated the EF factor scores with statistical learning scores at age 14. No significant correlations were found either with the WM (*r* = −.06, *p* = .546, BF_01_ = 2.40, posterior mean r = −.056, [−.257, .138]) or Fluency factor (*r* = .11, *p* = .271, BF_01_ = 3.59, posterior mean r = .108, [−.087, .299]). In addition, we examined the relationship between statistical learning scores at age 7 and the EF factors measured at age 14, and found no significant correlation either with the WM (*r* = .09, *p* = .402, BF_01_ = 2.88, posterior mean r = .085, [−.119, .290]) or the Fluency factor (*r* = .14, *p* = .210, BF_01_ = 1.92, posterior mean r = .130, [−.079, .335]). The BFs provide anecdotal to moderate evidence for the null hypotheses, suggesting a lack of relationship between statistical learning and EF cross-sectionally. In conjunction with the results of the logistic regression, this indicates that the relevant information lies not in isolated snapshots of SL ability, but in how SL develops over time.

### Are the results driven by age-related changes in overall reaction times?

A central and recurring concern in developmental psychology is the variation in baseline RTs across the lifespan. It is well-established that baseline RTs follow a U-shaped trajectory with age: both children and older adults tend to exhibit slower RTs than young and middle-aged adults across a range of tasks. One could argue that slower RTs provide more room to improve in learning tasks such as the ASRT^12^. To examine whether the observed longitudinal trajectory of statistical learning was due to age-related changes in overall RTs, we conducted three sets of control analyses.

First, if lower RTs are generally associated with lower learning scores (leading to a ceiling effect), this should be evident not only across ages but also *between individuals* within a certain age group. To test this, we performed correlational analyses, and found no significant association between overall RTs and statistical learning scores at any age (age 7: *r* = .17, *p* = .127, BF_01_ = 1.34 (posterior mean r = .155, [−.052, .357]); age 8: *r* = .11, *p* = .406, BF_01_ = 2.51 (posterior mean r = .100, [−.146, .327]); age 11: *r* = .10, *p* = .406, BF_01_ = 2.62 (posterior mean r = .090, [−.150, .314]); age 14: *r* = .16, *p* = .116, BF_01_ = 1.32 (posterior mean r = .151, [−.040, .346]); anecdotal evidence in favour of the null hypothesis). A similar absence of correlation between overall RTs and statistical learning scores was reported across nine distinct age groups—ranging from 7-8 to 61-85 years—in a cross-sectional ASRT study spanning the human lifespan^12^. Our results, in line with the findings of Juhasz et al.^12^, provide little credible evidence that lower RTs are associated with lower learning scores.

Second, following the same logic, if lower RTs were systematically associated with lower statistical learning scores (e.g., due to a ceiling effect), this relationship should also be evident *within individuals* in a single ASRT session. In other words, for a given individual, the blocks in which they respond faster should also show lower learning scores, resulting in a positive within-subject correlation. To test this, we used repeated measures correlation^44^ to assess the association between median RTs and learning scores across blocks for each session. We restricted this analysis to the second half of the task (blocks 11–20), where learning is more likely to have stabilised. The rationale for this restriction is that the ASRT task combines two processes: 1) participants respond increasingly faster to high-probability compared to low-probability triplets (reflecting statistical learning), while 2) also becoming faster overall as the task progresses (general practice-related speed-up). These processes can create a spurious negative correlation between RTs and learning scores. By focusing on the second half of the task, when most learning has already taken place, we aimed to minimise this confounding influence. This analysis revealed a statistically significant, weak positive relationship at age 7, *r*_*rm*_(755) = .100, 95% CI = [.029, .170], *p* = .006, BF_01_ = 0.25, posterior mean *r*_*rm*_ = .100, [.030, .170], but no significant relationships at any other age (Age 8: *r*_*rm*_(547) = .006, 95% CI = [−.078, .090], *p* = .890, BF_01_ = 10.00, posterior mean *r*_*rm*_ = .007, [−.077, .090]; Age 11: *r*_*rm*_(600) = .014, 95% CI = [−.066, .093], *p* = .738, BF_01_ = 10.00, posterior mean *r*_*rm*_ = .014, [−.068, .095]; Age 14: *r*_*rm*_(859) = .030, 95% CI = [−.037, .097], *p* = .377, BF_01_ = 8.33, posterior mean *r*_*rm*_ = .031, [−.035, .097]). Although a statistically significant positive correlation was found at age 7, the association between overall RTs and learning scores was very weak. Importantly, we found no significant associations at other ages and the corresponding Bayes Factors provided moderate evidence in favour of the null hypothesis. Notably, this includes age 14, a critical point in our dataset where potential confounds might arise due to near-ceiling performance, as reflected in the lowest overall RTs in the dataset. For completeness, we also conducted the correlation analyses across the full task (all 20 blocks; Supplementary Note S5). These analyses yielded either no significant correlation (age 7) or weak negative correlations (ages 8, 11, and 14), which we interpret as reflecting the confounding influence of ongoing learning. Altogether, based on these correlation analyses, we found little credible evidence that differences in overall RTs confounded our main results.

**Fig. 4:**
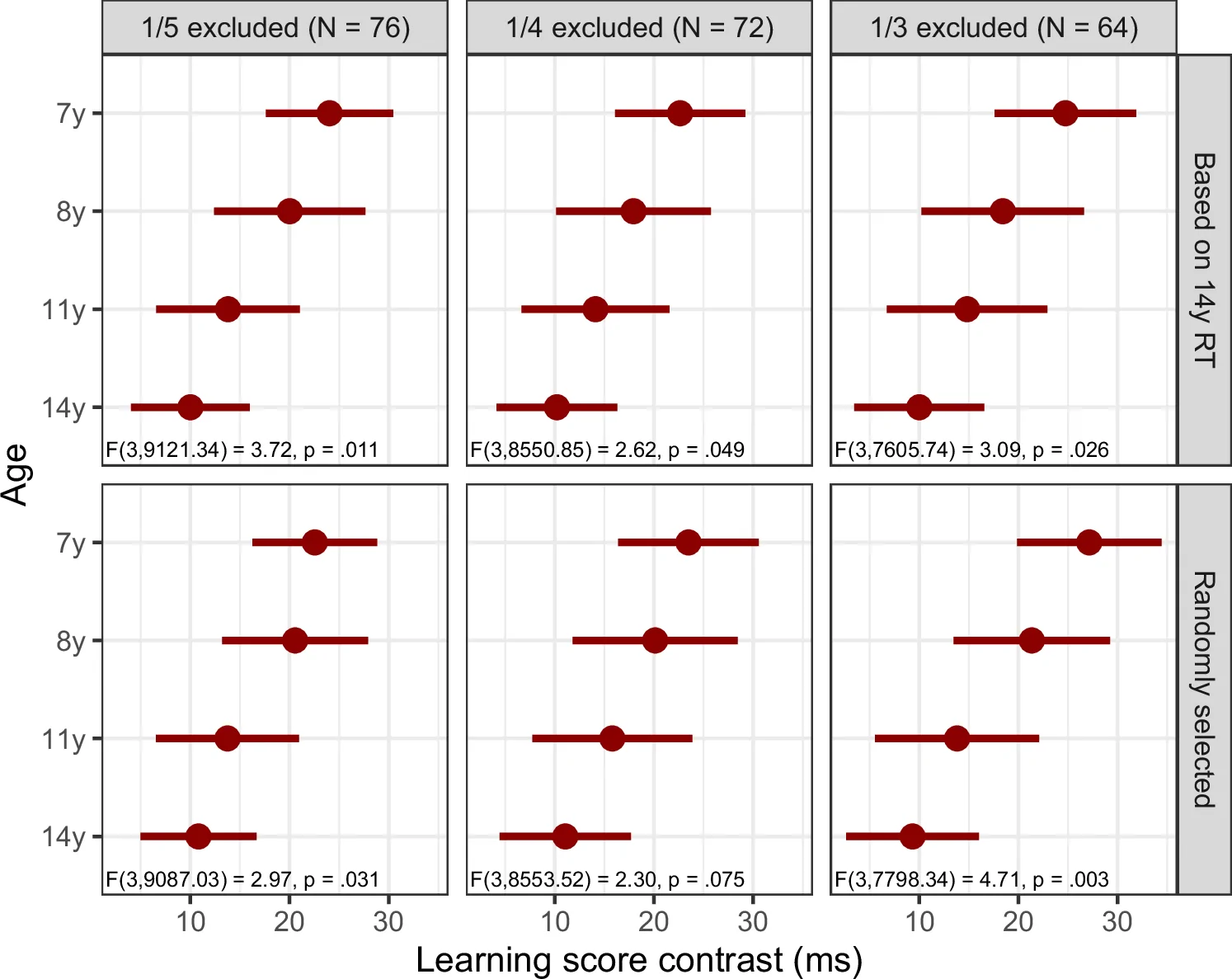
Control analyses of baseline RT. Sensitivity analyses with listwise exclusion of participants based on either their RT at age 14 (top), or at random (bottom). Shown are the mean learning score contrasts and their 95% CI on the x-axis as a function of age on the y-axis, separately for excluding one third, one fourth, or one fifth of participants. The results of the corresponding F test of the Age × Triplet type interaction are also shown on the bottom left of each panel. All *p-*values are two-sided and unadjusted for multiple comparisons. It is clear that the decreasing statistical learning scores are present in all models.

Third, we conducted an additional analysis based on an approach proposed in the literature to account for potential confounding effects of overall RTs on learning measures^12^. This approach involves selecting subsets of participants with comparable overall RTs within each age group and testing learning effects within these subgroups. Using this method, Juhasz et al.^12^ showed that the greater within-session improvement in younger age groups could not be explained by generally slower RTs. This approach had to be adjusted for our longitudinal design as selectively including only certain assessment timepoints for a participant—while discarding others—would have undermined the integrity of the dataset. Therefore, we excluded participants who at age 14 fell within the lowest fifth of the sample in terms of their overall RTs and thus were the most likely to have reached ceiling performance, and reanalysed these data (*N* = 76). The Age × Triplet type interaction remained significant (*F*_(3,9121.34)_ = 3.72, *p* = .011), and the age-related decrease in statistical learning was still clearly present (learning score contrasts at Age 7: 24.00 ms, 95% CI = [17.61, 30.50]; Age 8: 20.00 ms, 95% CI = [12.40, 27.60]; Age 11: 13.80 ms, 95% CI = [6.56, 21.00]; Age 14: 10.00 ms, 95% CI = [4.02, 16.00]). We replicated this analysis while excluding the fastest fourth and fastest third of participants, respectively. The results remained remarkably consistent across all models (see Fig. 4). In addition, to demonstrate that any changes in these models (wider confidence intervals and variability in *p*-values) reflect reduced statistical power due to the smaller sample size rather than selective exclusion of participants with the lowest overall RTs at age 14, we conducted further sensitivity analyses. We repeated the subsampling procedure, this time excluding the same number of participants at random instead of based on their RTs. These sensitivity analyses yielded highly comparable results, indicating that reduced sample size—not RT-based selection—was responsible for the wider confidence intervals and variability of p-values for the Age × Triplet Type interaction. Across all analyses, the age-related decline in statistical learning remained a consistent and robust finding.

## Discussion

The aim of our study was to address key limitations of cross-sectional research on the development of statistical learning. To achieve this, we conducted a longitudinal investigation tracking changes in statistical learning from ages 7 to 14, while also examining the role of top-down cognitive processes. Our findings reveal a gradual decline in statistical learning across development, which appears to be associated with the emergence of stronger top-down cognitive control observed by age 14.

Previous cross-sectional studies have shown varied developmental trajectories in statistical learning, potentially due to differences in the type of input^21^. Purely visual statistical learning and auditory statistical learning with non-linguistic stimuli seem to increase with age^10,15,16^, while auditory statistical learning with linguistic stimuli seems to be comparable across development^15,45^. The different developmental trajectories may be explained by linguistic entrenchment—that is, the idea that existing knowledge of language makes it harder to learn new linguistic patterns^46^. Visuomotor statistical learning, when employing probabilistic regularities, seems to decrease with age^11,12,14^, while with deterministic regularities, no changes were found from age 5 to 6 years^47^ and an inverted U-shaped trajectory was shown with a peak in young adulthood^13^. The present study employed a visuomotor statistical learning paradigm with probabilistic regularities and found a decreasing profile, consistent with the cross-sectional literature^11,12,14^. However, unlike cross-sectional studies that identified a step-like decline around 11–13 years of age, our longitudinal study revealed a progressive decline across development, with a relatively constant magnitude. Discrepancies between cross-sectional and longitudinal studies are not uncommon. For instance, Keresztes et al.^48^ and Seblova et al.^49^ found differences in hippocampal development and cognitive abilities, respectively, when comparing cross-sectional and longitudinal data: cross-sectional studies suggest hippocampal subfield volumes increase during middle childhood and early adolescence, whereas longitudinal evidence indicates a decrease across most subfields during this period. Cross-sectional studies often fail to account for generational differences and aging effects, whereas longitudinal studies, by tracking the same individuals over time, are better suited for causal interpretations. Our findings emphasise the importance of longitudinal designs in understanding the development of statistical learning and its interaction with top-down cognitive processes.

Given that the brain functions as an ecosystem, it is suboptimal to examine cognitive functions in isolation. Instead, it is beneficial to concentrate on the interplay among various cognitive processes. Therefore, here, we also considered the potential relationship between a broader cognitive function, such as EF and the development of statistical learning. We found that interindividual differences in EF ability were related to statistical learning trajectories. Specifically, children with higher EF abilities at age 14 were more likely to show a decreasing profile of statistical learning with development. In contrast, children with lower EF abilities at age 14 were more likely to exhibit an increasing profile of statistical learning. Prior cross-sectional studies suggested a competitive relationship between statistical learning and top-down cognitive processes in adults (e.g., refs. ^29, 50–52^, although in some cases, a positive relationship has also been found, see refs. ^33, 53, 54^). Our results revealed a negative association between the developmental trajectory of statistical learning between ages 7 and 14 and executive functions at 14. Notably, we found little credible evidence that EF measures at age 14 correlated with statistical learning scores at either age 7 or 14, with Bayes factors providing anecdotal and moderate support for the null hypothesis. The lack of cross-sectional correlations in our sample might be explained by developmental asynchrony. In adults, both systems underlying SL and EF are fully mature, enabling stable patterns of functional interaction. In contrast, the developmental timing of these systems is asynchronous in younger populations: SL typically reaches its peak level earlier in childhood, whereas EF continues to develop into late adolescence and young adulthood^55,56^. This developmental asynchrony may explain why cross-sectional associations emerge in adults but are absent or inconsistent in our younger sample. We also need to acknowledge an important limitation of our dataset: executive functions were assessed only once, at age 14. This inevitably constrains our ability to disentangle the mechanisms underlying the observed associations. Future studies should therefore examine the relationship between SL and EF in late adolescence, when both systems are closer to maturity, to determine more clearly whether they compete with one another during development.

In developmental psychology, the U-shaped trajectory of baseline RTs across the lifespan poses a methodological challenge when interpreting results from RT-based tasks. A central concern is whether age-related differences in learning scores are driven by differences in baseline RTs—namely, that faster responders have less room to improve, leading to apparent age-related declines in learning. To address this issue, we conducted a series of control analyses, which provided compelling evidence against this interpretation. Specifically, we found no significant relationship between baseline RTs and statistical learning scores, either between or within individuals, at any age—except for a very small effect at age 7. The corresponding Bayes Factors provided anecdotal to moderate support for the null hypothesis. Furthermore, we conducted additional analyses on subsampled data, excluding the fastest participants at age 14—those most likely to reach a performance ceiling—and consistently observed the same age-related decline in statistical learning. Although transforming RTs is a common approach to control for baseline RT differences, we chose not to apply it, as transformation can alter RT variability and potentially obscure meaningful information (for an illustration of how different transformation strategies distort distributional properties of the dataset, see Supplementary Note S6 and Fig. S12). In our data, RT variability was positively correlated with learning scores at age 14, but not at ages 7, 8, or 11 (Supplementary Note S6 and Fig. S11). This suggests that greater RT variability may be linked to more effective learning—an association that appears to emerge over the course of development. Taken together, we found no evidence that the observed developmental decline in statistical learning can be explained by baseline RT confounds.

The presence of distinct learning trajectories may help explain individual differences in learning styles and responsiveness to instruction, particularly in educational contexts that implicitly rely on statistical learning, such as language acquisition, reading, or music (e.g.^57^). Identifying learners who are more or less sensitive to statistical regularities could inform the development of tailored interventions or training strategies. For example, individuals with stronger top-down control may benefit from learning environments that make regularities more explicit or reduce reliance on incidental pattern detection. Conversely, early learners with high sensitivity to statistical input may thrive in less structured, exploratory environments, in which top-down cognitive processes are required to a lesser extent. Taken together, the identification of distinct statistical learning trajectories—and their relationship to EF—provides a valuable framework for understanding developmental heterogeneity in learning processes. It also highlights the need for educational approaches and cognitive models that account for these individual differences across development.

In summary, we found a decrease in statistical learning in a longitudinal design between the ages of 7 and 14 years. This decreasing profile was associated with better executive functioning, providing empirical evidence for a negative relationship between statistical learning and top-down cognitive processes in a longitudinal setting. We propose that the ability to construct new predictive models from environmental inputs varies with age, with younger children being more adept at this process. This may explain why younger children excel in language acquisition and the development of new skills. Our findings also highlight the importance of considering individual variability and developmental changes when studying cognitive functions, emphasising the nuanced interplay among cognitive processes across the lifespan.

## Methods

### Participants

A total of 135 participants were initially recruited for the study, which commenced data collection in October 2010 and concluded in 2018. The sample consisted of children enrolled in the 1st grade of primary school within the Hungarian education system. The extent of missing data at each assessment is provided in Table 5. Given that our primary focus was the longitudinal, within-subject development of statistical learning, we excluded participants who did not complete at least two of the four assessments (24 participants). We also excluded participants with missing data on any of the neuropsychological tests (14 participants). This resulted in a final sample size of 97 participants (42 boys, 55 girls, age at first assessment: *M* = 6.81 years, *SD* = 0.55 years). Gender was recorded based on the participant's self-report. Gender was not included as a factor in the analyses because the study was not designed to investigate gender differences. Due to an experimental error, 17 participants were assigned the same sequence in the ASRT task (see below) at least twice. Since robust long-term memory consolidation effects have been documented for this task^8,58^, we conducted control analyses excluding these participants. The pattern of results remained the same in this smaller sample (see Supplementary Note S7), therefore, results of the full sample are reported in the main text. No further exclusions were made. No statistical method was used to predetermine the sample size. Instead, the sample size was determined based on previous studies using the same task. The sample represents a convenience sample rather than a representative sample of the general population. Legal guardians of all participants provided written consent and children assented to participate in the study before testing. Participants were not compensated for taking part in the study. The study was approved by the Research Ethics Committee of Eötvös Loránd University, Budapest, Hungary and was conducted in accordance with the Declaration of Helsinki.

**Table 5:** Descriptive statistics of the sample. Average ASRT performance at each age, and fraction of missing ASRT data at each age are reported

	1st assessment (Age 7)	2nd assessment (Age 8)	3rd assessment (Age 11)	4th assessment (Age 14)
ASRT mean accuracy (in %, M ± SD)	88.56 ± 9.65	88.78 ± 9.15	91.06 ± 7.96	93.55 ± 3.36
ASRT mean RT (in ms, M ± SD)	782.55 ± 177.01	588.91 ± 76.59	502.33 ± 58.61	404.39 ± 45.37
Participants with missing ASRT data (N, %)	13 (13.4%)	36 (37.1%)	30 (30.9%)	2 (2.1%)

No inferential statistics were computed on these descriptive data.

### Alternating Serial Reaction Time task

The Alternating Serial Reaction Time (ASRT) task was programmed in E-Prime version 2.0.10. The task involves presenting participants with a visual stimulus (a drawing of a dog’s head) in one of four horizontal locations on the screen, and participants are instructed to indicate the location of the target stimulus by pressing the corresponding key on the keyboard (Z, C, B, M) (Fig. 1A). The stimulus remained on the screen until the participants responded, then, after a 120-ms-long delay, the next target appeared. The presentation of stimuli followed an eight-element sequence, with pattern and random trials alternating with each other (e.g., 2 – r – 4 – r – 3 – r – 1 – r, where *r* indicates a randomly selected location out of the four possible ones, and the numbers represent the predetermined positions from left to right). Each participant was assigned one of six unique sequences (note that the sequence could start at any position, hence, 2 – r – 4 – r – 3 – r – 1 – r and 1 – r – 2 – r – 4 – r – 3 – r are identical sequences), which they were exposed to throughout the task. The ASRT task consisted of 20 blocks, with each block containing 10 repetitions of the 8-element sequence, following a 5-trial practice period, for a total of 85 trials per block. Feedback about performance (mean speed and accuracy) was provided after each block.

In the task, three successive trials are referred to as triplets. Due to the alternating sequence structure, some triplets occurred with a higher probability than others. For example, in the sequence 2 – r – 4 – r – 3 – r – 1 – r, triplets 2-X-4, 4-X-3, 3-X-1, and 1-X-2 (where X indicates the middle element of a triplet) occurred with a higher probability because the third element of the triplet could occur as part of the repeating sequence (Pattern) and could also occur in a random trial. This is illustrated in Fig. 1C for the example triplet 2-X-4, which could appear both in a Pattern-random-Pattern or a random-Pattern-random structure. In contrast, triplets such as 2-X-1 or 2-X-2 had a lower occurrence probability because they would never occur as part of the repeating sequence, only as a random-Pattern-random structure. The former triplet types are referred to as high-probability triplets, and the latter ones as low-probability triplets. Note that triplets were identified using a moving window approach. Thus, each trial was categorised as the third element of a high- or a low-probability triplet, and this categorisation was used in our analyses. The same trial also served as the middle element or the first element of the following triplets. For example, the random trial 4 in Fig. 1B served as the third element of the triplet 2-3-4, as the second element of the triplet 3-4-1, and as the first element of the triplet 4-1-2. Of all trials, 62.5% was categorised as the third element of a high-probability triplet and 37.5% was categorised as the third element of a low-probability triplet (Fig. 1C).

Previous research has robustly shown that people are sensitive to the statistical structure of the task and respond both faster and more accurately to high-probability triplets compared to the low-probability ones^8,11,33,59^. Individuals acquire the statistical structure incidentally (that is, without instruction) and implicitly^60^. The ASRT task shows relatively good reliability^61^.

### Executive function tasks

#### Counting span task

Updating or working memory capacity was measured by a computerised version of the Counting span (CSPAN) task^36^. In this task, different shapes (blue circles, blue squares, and yellow circles) appeared on the computer screen. Participants were asked to count out loud and memorise the number of blue circles (targets) among the other shapes (distractors) in a set of trials. Each trial consisted of one image, and included between 3 and 9 blue circles, between 1 and 9 blue squares and between 1 and 5 yellow circles. At the end of each trial, participants repeated orally the total number of targets presented in the image and if their response was correct, the experimenter moved on to the next trial. When presented with a recall cue at the end of a set, participants had to name the total number of targets of each image again in their order of presentation. If the recall was correct, participants started a new set containing an extra image. When participants made a mistake during recall, the task was stopped, and a new run, starting with a set of two trials, would begin. Each participant completed three runs of the task. Performance was operationalised as the mean of the largest set size the participant was able to recall correctly in the three runs.

#### Digit span task

Phonological short-term memory capacity was measured by the Digit span (DSPAN) task^62^. In this task, participants listened to and then repeated a list of digits enunciated by an experimenter. The task comprised seven levels of difficulty, ranging from three to nine items. Each level comprised four different lists. If participants correctly recalled all items for at least three out of the four lists, they moved up a level. If participants could not correctly recall at least three of the four lists, the task ended. Performance was operationalised as the level (three to nine) at which the participant was still able to recall three from the four lists correctly.

#### Backward digit span task

The Backward digit span (BackDSPAN) task is identical to the DSPAN, except that participants have to repeat the digits in reverse order. Performance was operationalised as the level (three to nine) at which the participant was still able to recall three from the four lists correctly.

#### Corsi block tapping task

Visuo-spatial short-term memory capacity was assessed using the Corsi block tapping task^63^. In this task, nine cubes were placed in front of the participant in a fixed pseudorandom manner. The blocks were labelled with numbers only visible to the experimenter. The experimenter tapped several blocks in a specific sequence, after which the participant had to tap them in the same order. The task comprised seven levels, each with three to nine items. Four sequences were presented within a level. Performance was operationalised as the level (three to nine) at which the participant was still able to recall three from the four sequences correctly.

#### Fluency task

Verbal fluency was measured with two subtasks testing the lexical and semantic components of verbal fluency^64^. In the lexical fluency subtask, participants were required to say as many words as possible that began with a given letter. There were two runs, one with the letter K, and one with the letter T. In the semantic fluency subtask, participants were required to name as many words as they could that belonged to a certain category. There were two runs, one with the category ‘animals’, and one with the category ‘things you can find in a supermarket’. In each subtask, participants were instructed to say as many words as possible within one minute while avoiding word repetitions, words with the same etymological root and proper nouns. The task was conducted in Hungarian, the native language of all participants. Performance was operationalised as the total number of correct words produced within one minute, and averaged across the two runs for each subtask.

### Procedure and study design

**Fig. 5:**
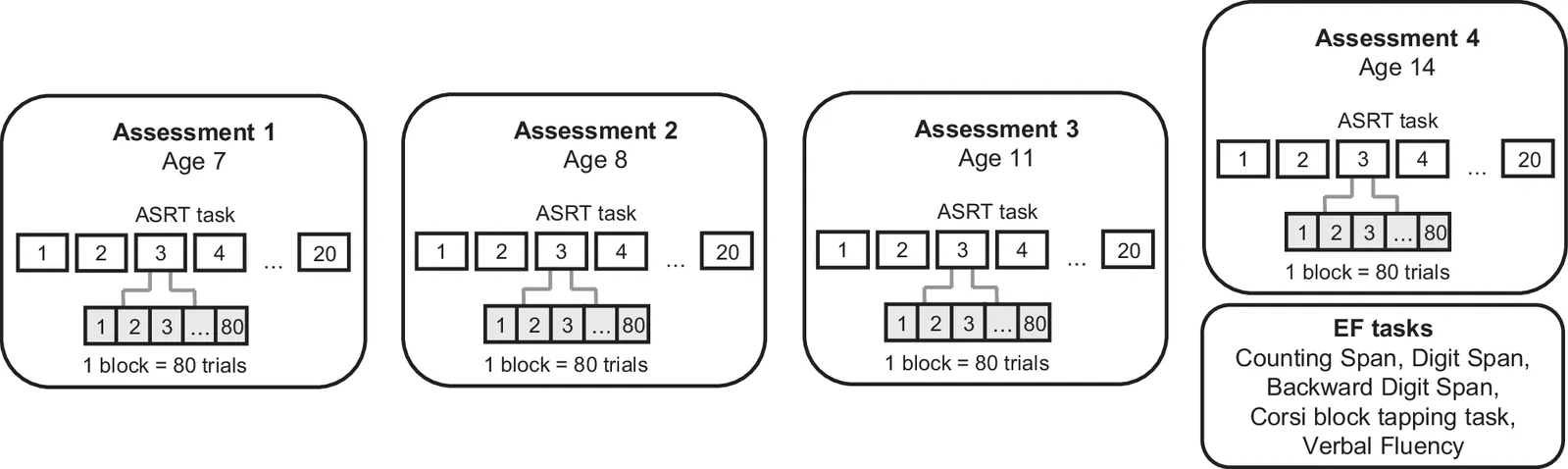
Study design. In our longitudinal design, participants took part in four assessments conducted at ages 7, 8, 11 and 14. During each assessment, they completed 20 blocks of 80 trials of the ASRT task, and during the fourth assessment, they also completed executive function (EF) tasks.

Our longitudinal study included four assessments conducted at ages 7 (1st grade), 8 (2nd grade), 11 (5th grade) and 14 (8th grade) (see Fig. 5). All assessments were performed at the participants’ schools. At each assessment session, participants first completed the ASRT task. In accordance with standard protocol, participants were administered a series of questions at the end of the ASRT task to evaluate conscious knowledge of the hidden sequence. Specifically, they were asked if they noticed anything unusual or any regularities in the task, and if so, to elaborate on their response^65–67^. None of the participants could describe the alternating sequence at any point in the assessment. This was then followed by a questionnaire regarding demographic data and supplementary information. As part of the broader data collection process, participants also completed other experimental tasks and neuropsychological tests, which are not analysed here. Each assessment session lasted approximately two hours.

### Statistical analysis

Each trial of the ASRT task was categorised based on the two preceding trials as the last element of a high- or low-probability triplet. Trills (e.g., 1-2-1) and repetitions (e.g., 2-2-2) were removed from the data, as participants can show pre-existing response tendencies, such as automatic facilitation, to these types of trials^68^. Trials with reaction times (RTs) below 100 ms were also removed, as these were likely errors due to inattention or distractors. Inaccurate responses were removed from the analyses of RTs. We calculated mean accuracy and median reaction times for each participant and each block, separately for high- and low-probability trials. The mean accuracy scores at the different time points were between 88.56% and 93.55% (Table 5). As high accuracy scores and relatively low variance in typically developing participants can hinder the detection of learning^69^, we used RTs as our primary measure. Analyses on accuracy scores are reported in the Supplementary Information (Supplementary Note S1).

One of the strengths of the ASRT is that it allows for a separation of sequence-specific statistical learning from general practice-related performance improvements. *Statistical learning* was operationalised as the difference in RTs between high- and low-probability trials. An alpha level of .05 was applied to all analyses detailed below.

For correlational analyses, we used Pearson’s correlation coefficients. Bayes factors (BFs) and posterior estimates were computed with the correlationBF function from the BayesFactor R package (https://search.r-project.org/CRAN/refmans/BayesFactor/html/correlationBF.html), sampling for 3000 iterations using Jeffreys’s default priors with scale *r* = 1/3^70^. BFs were interpreted following Lee and Wagenmakers^71^. For the sensitivity analysis involving within-subjects correlations between learning scores and RTs across different blocks, we employed repeated measures correlation, using the *rmcorr* R package^44^. Repeated measures correlation estimates the within-individual association between two continuous variables while accounting for the non-independence of repeated observations. It removes between-subject variance by fitting parallel regression lines (same slope, different intercepts) and computing a shared slope-based correlation coefficient. This approach provides a more precise measure of within-subject associations than traditional methods. Bayesian posterior estimates of the within-subject association were obtained using multivariate Bayesian mixed-effects models fitted with the *brms* package. Standardised learning scores and RTs were modelled jointly with participant-specific random intercepts, and the residual correlation between the two outcomes was interpreted as the within-subject correlation. Weakly informative priors were specified for all parameters, including Normal(0, 1) priors for the intercepts, Student-*t*(3, 0, 1) priors for participant-level and residual standard deviations, and LKJ(2) priors for both the within-subject residual correlation and the between-subject random-intercept correlation. Bayes factors were calculated using the *BayesFactor* package by comparing a mixed-effects model predicting RT from learning score and participant with a null model containing participant only. We report posterior means and 95% credible intervals for all aforementioned Bayesian analyses.

#### Linear mixed models

In reporting our linear mixed model (LMM) analyses, we follow best practice guidelines proposed by Meteyard and Davies^72^. All pre-processing and statistical analysis were performed in *R* 4.2.1. Linear mixed models were fit with the *mixed* function from the *afex* package^73^, which enabled appropriate effects coding and accounted for interactions in the models^74^. No a priori power analysis was done for the study. Our primary independent variables of interest were Block, Age and Triplet type. Triplet type was a factor variable, effects coded to reflect the high- and low-probability triplet categories, with high-probability triplets being the reference category. Block was treated as a continuous variable and was mean-centred before analysis. No other data transformation was carried out. Assumptions regarding linearity, homoscedasticity and normality of residuals were evaluated by scatterplots and QQ plots of residuals. Assumptions of linearity and homoscedasticity were met in each case; there was obvious non-normality of residuals of the models. We note however, that fixed effects estimates of LMMs have been shown to be remarkably robust against violation of this assumption^75^. Therefore, we deemed our models adequate.

Our model building was guided by a priori, theoretical considerations and our specific use of random effects to control for overall RT differences between individuals and assessment timepoints, no model comparisons were made. Our main model included Age, Block, and Triplet type, and all their higher-order interactions as fixed effects. Age was effects coded (contr.sum R function). Our random effects structure included uncorrelated by-participant intercepts and slopes for Block, Age, and their interaction. We note that this model converged with a gradient somewhat above the standard tolerance level of 0.002. Therefore, we compared fits with different optimisers, using the allFit function of *afex*. If parameter estimates were consistent across optimisers, we deemed the model adequate (our default optimiser was bobyqa). This was the case.

Sample sizes in terms of total number of data points and of sampling units, random effects estimates, Nakagawa’s marginal and conditional R^2^^76,77^, and the adjusted ICC are all consistently reported in summary tables.

Post-hoc contrasts were conducted with the *emmeans R* package^42^. For inference about fixed effects, we used Type III tests, relying on comparing nested models with the effect of interest either included or removed. We used the Satterthwaite approximation of degrees of freedom^78^. Figures were created with *ggplot2*^79^.

As a sensitivity analysis, the primary LMM was also estimated in a Bayesian framework using the *brms* package. The model specification (fixed and random effects structure) was identical to the frequentist model. Weakly informative priors were specified for all parameters, comprising a Normal(600, 200) prior for the intercept, Normal(0, 100) priors for fixed effects, and Student-*t*(3, 0, 100) priors for participant-level standard deviations and the residual standard deviation. Models were estimated using four Markov chain Monte Carlo chains of 10,000 iterations each (2,000 warm-up iterations), with convergence assessed by inspection of *Rhat*, effective sample sizes, and posterior trace plots. Posterior means and 95% credible intervals are reported.

#### Latent class analysis

To explore whether our participants could be classified into different subgroups based on the developmental trajectory of their statistical learning, we employed latent class analysis (LCA) using the *lcmm* R package^80^. In reporting our LCA analyses, we followed the GRoLTS best practice guidelines proposed by van de Schoot et al^81^. The outcome variable in these models was an age-wise learning score index. Since in these analyses we were specifically interested in between-subjects variability, we derived the learning scores using a two-stage averaging procedure (first averaging by five blocks, then for the whole task), which has been shown to yield the most reliable learning indices in the ASRT^61^. The time variable of interest was Age, coded as 0, 1, 4 and 7, representing the temporal separation (in years) between each assessment point. Variance in participant age was relatively low within assessment timepoints. We assumed data were missing at random, although no formal tests were conducted. Missing data were handled using full information maximum likelihood during model estimation.

We employed the growth mixture model approach, which assumes a heterogeneous population composed of *G* latent classes of participants characterised by *G* mean profiles of trajectories. Within each class, between-participant variability is captured by participant-specific random intercepts and slopes. The probability of belonging to each class is computed for each participant, with classification based on the highest probability. Models were fit using maximum likelihood, with starting values for the algorithm determined by a grid search implemented by the gridsearch function of *lcmm*. This involved randomly generating 200 sets of initial values for the model from the estimates of an initial 1-class baseline model, then using these values to run the optimisation algorithm for a maximum of 50 iterations and retaining the iteration with the best log likelihood.

We fit a series of models with up to 3 latent classes (more complex models did not converge), and selected our main model based on a combination of conceptual and statistical arguments. Given our relatively small sample size for these models, our primary goodness-of-fit index was the sample-size-adjusted Bayesian information criterion (SABIC), but we also report the Akaike information criterion (AIC). In addition to the SABIC, we considered the size of classes and the posterior probabilities of class membership as additional criteria for model selection. Classification performance was evaluated using the mean posterior probability within each class, as well as the entropy. After identifying our model of choice, we used the 3-step strategy for the analysis relating to predicting class membership. This involved first determining the number of latent classes without the predictors on class membership (Step 1), then saving the most likely class membership (highest posterior probability) (Step 2). Finally, we used these classes as the outcome of a multinomial or logistic regression analysis (Step 3), weighted by the posterior probabilities of class membership^82^. We also quantified the joint contribution of the two EF factor scores to improving model fit with a Likelihood Ratio Test.

In addition, to investigate the robustness of the classes uncovered in our primary analysis, we used the comprehensive *latrend* package to fit a series of longitudinal clustering models spanning algorithmic families^83^. Specifically, we fit a regression-based k-means model (LMKM), a mixed-model–based k-means model (GCKM), a group-based trajectory model (GBTM)^43^, and a growth mixture model with random intercept only (GMM1), along with our original growth mixture model including random slopes (GMM2). Within each model type, we arbitrated between different numbers of classes using the same criteria as our GMM2 model. We assessed the agreement between models qualitatively by comparing the trajectories of the extracted classes, and quantitatively by using the Adjusted Rand Index^84^ and Normalised Mutual Information^85^. To investigate the robustness of our logistic regression analysis, we repeated the procedure and predicted class assignment from the two EF factor scores for each model class, using either weighted multinomial regression (for models with a large enough third class, LMKM and GCKM) or weighted logistic regression.

### Reporting summary

Further information on research design is available in the Nature Portfolio Reporting Summary linked to this article.

## Supplementary information

Supplementary Information

Reporting Summary

Transparent Peer Review file

## Data Availability

All data reported in this study are available to download via https://doi.org/10.17605/OSF.IO/GRBD4.

## Code availability

The codes for data analyses are available via https://doi.org/10.17605/OSF.IO/GRBD4.
