# Supplementary material for: Longitudinal evidence for decreasing statistical learning abilities across childhood
Source: Nat Commun. 2026 Sep 3;17:10471. doi: 10.1038/s41467-026-77261-1 (PMC13635216; doi:10.1038/s41467-026-77261-1)
Supplement: Supplementary file 1 [file 41467_2026_77261_MOESM1_ESM.pdf]

Supplementary Information of the manuscript entitled

"Longitudinal evidence for decreasing statistical learning abilities across childhood"

Authors: Eszter Tóth-Fáber, Bence C. Farkas, Tímea Tánczos, Dezső Németh, Karolina Janacsek

Supplementary Note S1 - Developmental trajectories of statistical learning in accuracy

Block wise accuracy was used as the outcome variable in a linear mixed model including Triplet type (high- vs. low-probability), Block (Block 1-20), and Age (7, 8, 11 or 14), as well as all their higher order interactions as fixed effects, and uncorrelated by-participant intercepts and slopes for Block, Age, and their interaction as random effects.

Model results are summarized in Table S1 and S2, mean learning trajectories are plotted in Supplementary Fig. S1. We first summarise the random effects (see also Fig. S2B). The adjusted ICC of the model was .33, indicating moderate clustering in the data. Variance estimates at each level revealed clustering in both intercepts, reflecting interindividual variability in overall baseline accuracy (SD = 5.39), and at different ages (SD_{Age7} = 6.87, SD_{Age8} = 6.19, SD_{Age11} = 5.82), with very little variance of the slopes of Block (SD = 0.30) or of the Age × Block interaction ($SD_{Block \times Age7}$ = 0.64, $SD_{Block \times Age8}$ = 0.55, $SD_{Block \times Age11}$ = 0.47). This indicates that most interindividual variability came from differences in initial accuracy at different ages, with relative homogeneity in the amount of speedup within blocks and in the trajectories across ages. Residual variance was SD = 7.69.

Now we describe the fixed effects. A significant main effect of Block was revealed ($F_{(1,75.02)}$ = 34.74, p < .001), surprisingly indicating decreasing overall accuracy throughout the task (b = -0.22, 95% CI = [-0.29, -0.14]). This effect indicates that on average, contrary to reaction times, participants seemed not to improve in terms of accuracy. These decreases in accuracy might stem from fatigue or the waning of attention. The main effect of Age was significant as well ($F_{(3,126.41)}$ = 4.98, p = .003), indicating increasing overall accuracy during development (Age 7: 87.7 %, 95% CI = [85.8, 89.5]; Age 8: 88.3 %, 95% CI = [86.2, 90.3]; Age 11: 90.6 %, 95% CI = [88.8, 92.5]; Age 14: 93.1 %, 95% CI = [90.7, 95.6]; with age 14 being higher than age 7 (p = .008) and 8 (p = .021) and age 11 being higher than age 7 (p = .036)).

The model also resulted in a significant main effect of Triplet type ($F_{(1,11673.58)}$) = 203.99, p < .001), indicating higher accuracy for high-probability triplets than for low-probability triplets (high: 90.9 %, 95% CI = [89.8, 92.1]; low: 88.9 %, 95% CI = [87.8, 90.1]). This effect indicates that on average, participants engaged in statistical learning. The Block × Triplet type interaction also reached significance ($F_{(1,11673.58)}$ = 15.87, p < .001), stemming from a shallower decreasing slope for high-probability (b = -0.168, 95% CI = [-0.245, -0.091]), than low-probability triplets (b = -0.266, 95% CI = [-0.343, -0.189]), indicating increasing statistical learning with time within task. The lack of significant two-way Triplet type × Age and three-way Triplet type × Age × Block interactions indicate that contrary to reaction times, for accuracy, both the overall amount and the dynamics of statistical learning during the task remained relatively constant. Similarly, the lack of a statistically significant two-way Block ×

Age interaction indicates that the dynamics of overall accuracy remained relatively constant with development.

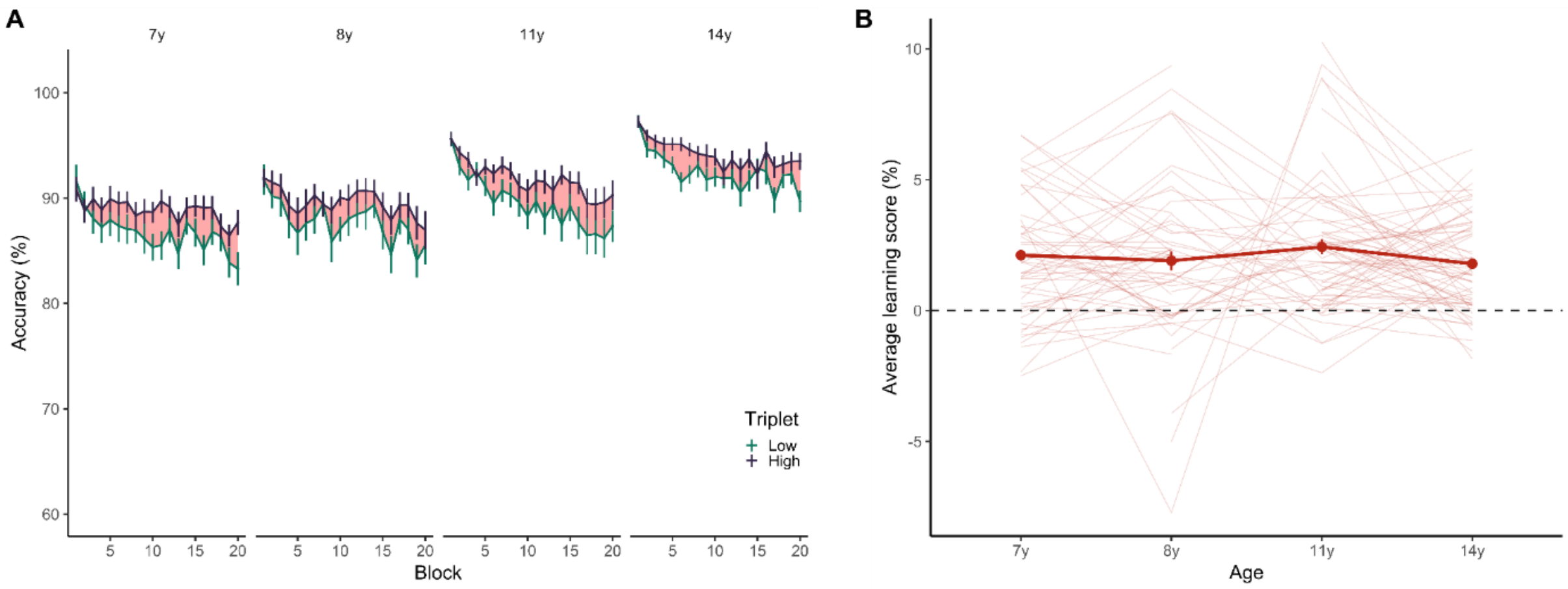

Figure S1. Trajectories of statistical learning in accuracy. A) Average block-wise accuracies as a function of triplet type (green for low-probability, indigo for high-probability) for the 4 ages tested. Differences in accuracy between the more (high-probability) and less predictable (low-probability) stimuli indicate statistical learning and are highlighted by light red shading. Visually, statistical learning is marked by the distance between the green and indigo lines. Linear mixed models revealed that the amount of statistical learning stayed constant with development. **B)** Whole task average learning scores for the 4 ages. Learning scores indicate the overall amount of statistical learning and are calculated by subtracting accuracy scores between the high- and low-probability triplets (corresponding with the light red shading of panel A). Individual learning trajectories are shown by the light red lines. In all subpanels, points indicate the mean, error bars indicate 1 within-subjects standard error of the mean calculated by the method of Morey[8] and data are shown for N = 97 participants.

Table S1. Type III test of effects of the linear mixed model, predicting block-wise accuracy.

**Type III test of effects**	*F*	*df*	*p*
Age	4.98	3, 126.41	.003*
Block	34.74	1, 75.02	< .001*
Triplet type	203.99	1, 11673.58	< .001*
Age × Block	0.72	3, 119.48	.543
Age × Triplet type	0.91	3, 11673.58	.434
Block × Triplet type	15.87	1, 11673.58	< .001*
Age × Block × Triplet type	0.89	3, 11673.59	.447

Notes. Significant terms are marked with an asterisk. All *p* values are two-sided and unadjusted for multiple comparisons.

Table S2. Fixed and random effects of the linear mixed model, predicting block-wise accuracy.

Fixed effects	*b*	*SE b*	*95% CI*	*t*	*df*	*p*
(Intercept)	89.93	0.59	88.77 – 91.10	153.37	92.98	< .001*
Age [7y]	-2.26	0.77	-3.78 – -0.73	-2.93	97.32	.004*
Age [8y]	-1.67	0.79	-3.23 – -0.11	-2.13	91.25	.036*
Age [11y]	0.72	0.72	-0.73 – 2.15	0.99	82.45	.323
Block	-0.22	0.04	-0.29 – -0.14	-5.89	75.02	< .001*
Triplet type [Low]	-1.01	0.07	-1.14 – -0.87	-14.28	11673.58	< .001*
Age [7y] × Block	0.03	0.07	-0.11 – 0.17	0.39	101.11	.694
Age [8y] × Block	0.03	0.07	-0.11 – 0.17	0.47	94.77	.640
Age [11y] × Block	-0.09	0.06	-0.21 – 0.03	-1.42	84.86	.159
Age [7y] × Triplet type [Low]	-0.08	0.12	-0.31 – 0.15	-0.70	11673.57	.486
Age [8y] × Triplet type [Low]	0.07	0.13	-0.18 – 0.33	0.54	11673.57	.586
Age [11y] × Triplet type [Low]	-0.13	0.13	-0.38 – 0.11	-1.06	11673.57	.290
Block × Triplet type [Low]	-0.05	0.01	-0.07 – -0.02	-3.98	11673.58	< .001*
Age [7y] × Block x Triplet type [Low]	-0.02	0.02	-0.06 – 0.02	-0.76	11673.57	.340
Age [8y] × Block × Triplet type [Low]	0.01	0.02	-0.03 – 0.06	0.51	11673.57	.556
Age [11y] × Block × Triplet type [Low]	-0.02	0.02	-0.06 – 0.02	-0.97	11673.57	.324

Random Effects	
σ^2	59.12 (7.69)
$\tau_{00\ \text{Participant}}$	29.300 (5.39)
$\tau_{11\ \text{Block}}$	0.09 (0.30)
$\tau_{11\ \text{Age[7y]}}$	47.24 (6.87)
$\tau_{11\ \text{Age[8y]}}$	38.27 (6.19)
$\tau_{11\ \text{Age[11y]}}$	33.92 (5.82)
$\tau_{11\ \text{Block × Age[7y]}}$	0.41 (0.64)
$\tau_{11\ \text{Block × Age[8y]}}$	0.30 (0.55)
$\tau_{11\ \text{Block × Age[11y]}}$	0.23 (0.47)
ICC	0.33
$N_{\text{Participant}}$	97
Observations	12318
Marginal R^2 / Conditional R^2	0.082 / 0.384

Notes. The table shows regression coefficients of fixed effects and summary information about the random effects. Random effects are presented as variance and SD in brackets. The marginal R-squared considers only the variance of the fixed effects, while the conditional R-squared takes both the fixed and random effects into account (based on Nakagawa et al.[9]). Degrees of freedom are based on Satterthwaite's approximation. Significant terms are marked with an asterisk. All *p* values are two-sided and unadjusted for multiple comparisons. Terms in brackets indicate the level of the factor that is contrasted against the reference level (High triplets for Triplet type and Age 14 for Age). Because we were interested in lower order effects too, both Age and Triplet type were effects coded, therefore lower order interactions and main effects are to be interpreted as deviations from the grand mean value of the dataset (at age 10 in our case, even though this value was not practically observed, and at the average of high- and low-triplets).
Model equation in *lmer* syntax: Accuracy ~ Age*Block*Triplet type + (Block*Age || Participant)

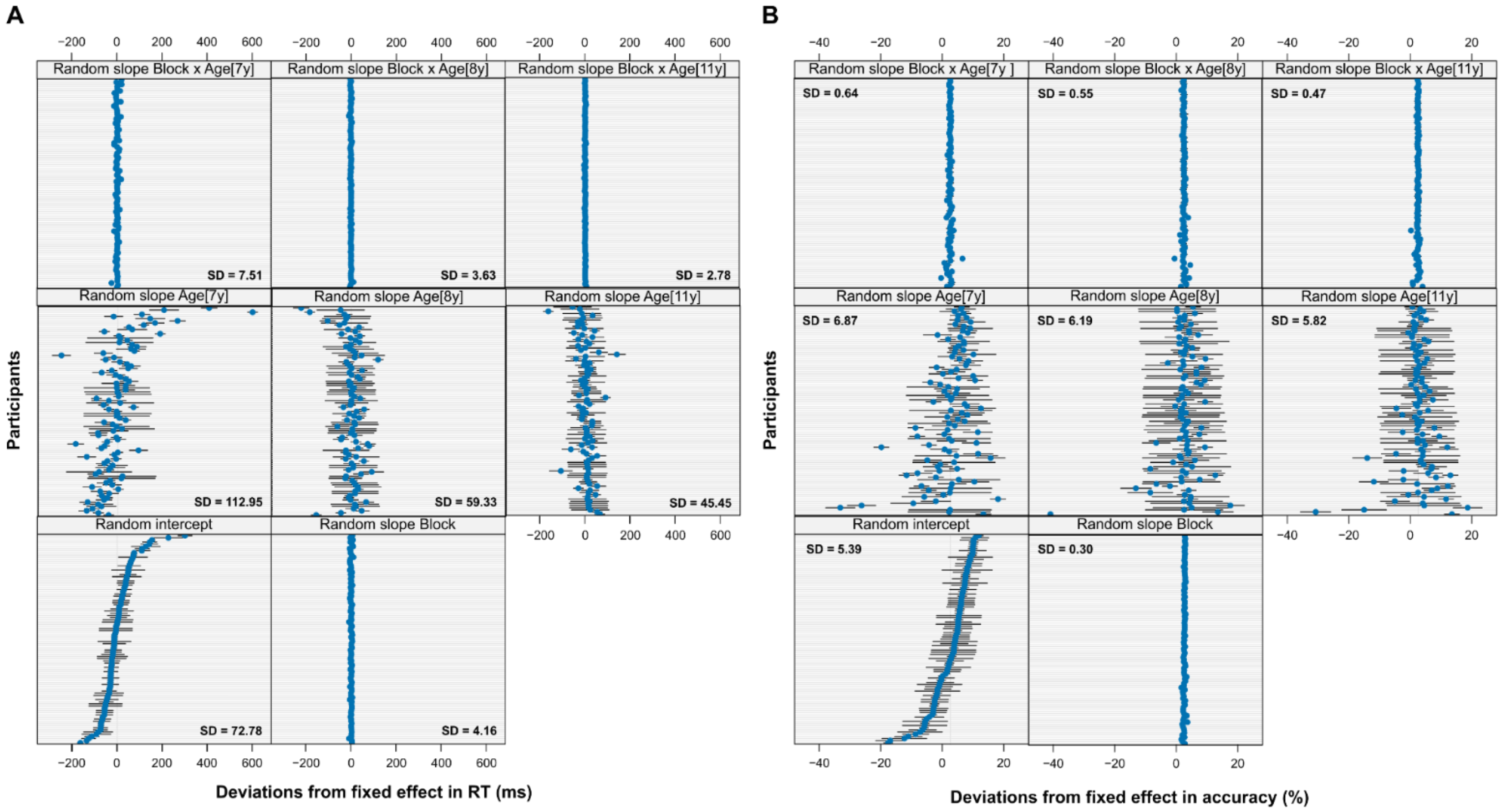

Figure S2. Caterpillar plots of random effects for RT (A) and accuracy (B). Participants (N = 97) are ordered in each panel according to their random intercepts (bottom left panel, representing deviations in baseline RT from the group average). Each row is one participant, error bars represent one standard deviation. The variance estimate for each random effect is presented in each subpanel as standard deviation.

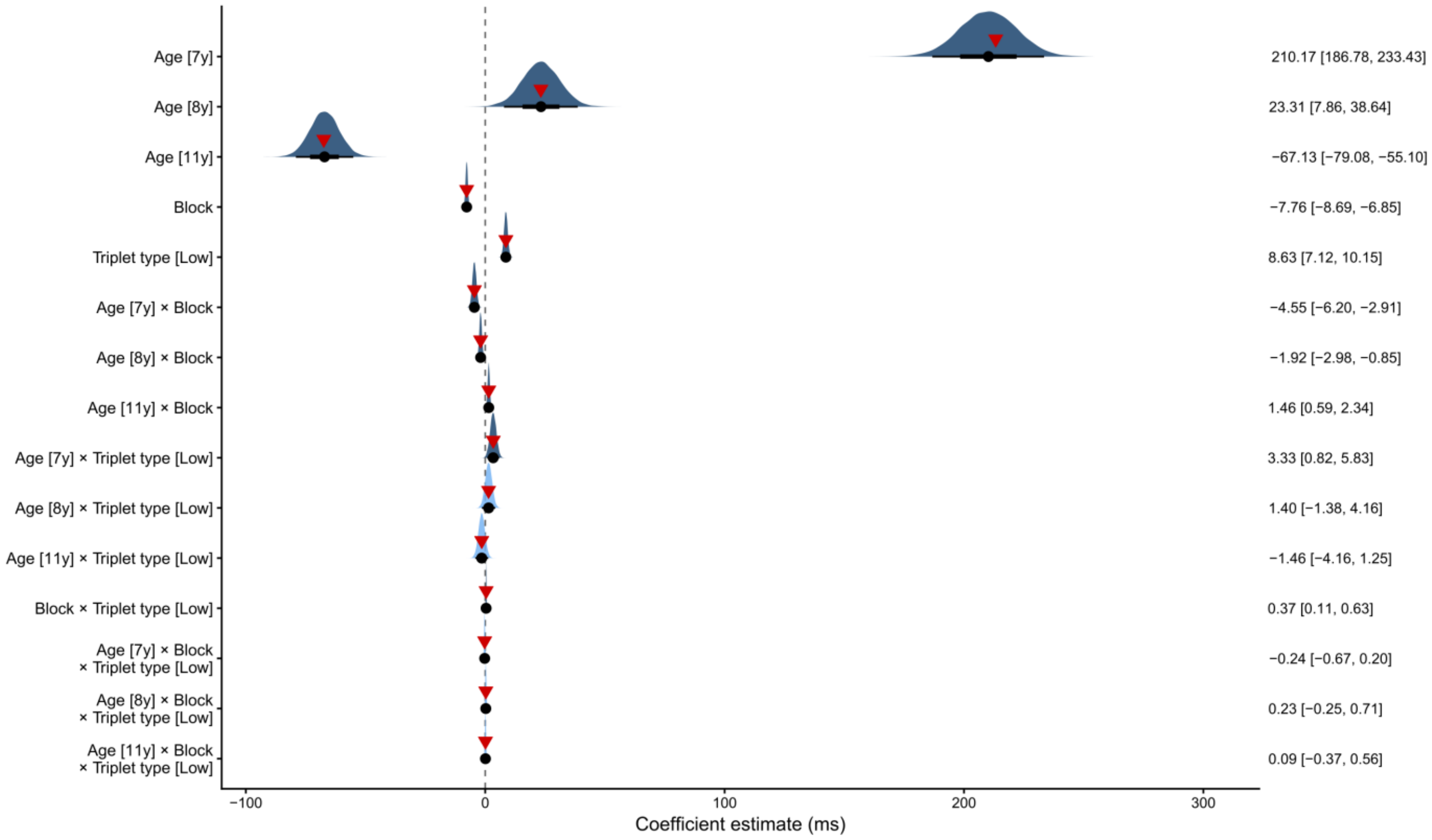

Figure S3. Posterior distributions of fixed-effect estimates from the Bayesian linear mixed-effects model of reaction times. Half-eye plots show the posterior distributions of the fixed-effect coefficients for the effects of Age, Block, Triplet type, and their interactions. Points indicate posterior medians, with thick and thin horizontal intervals representing the 68% and 95% credible intervals, respectively. Red triangles indicate the coefficient point estimates from the frequentist model reported in the main text. The vertical dashed line denotes a coefficient value of zero. Dark blue distributions indicate coefficients whose 95% credible interval excludes zero, whereas light blue distributions indicate coefficients whose 95% credible interval includes zero. Numerical labels on the right report the posterior mean and 95% credible interval for each coefficient. Positive coefficients indicate longer reaction times relative to the reference condition or contrast coding, whereas negative coefficients indicate shorter reaction times. All Rhats < 1.01, all bulk ESS > 1566.

Supplementary Note S2 – Description of the 1- and 2-class LCA models

1-class model

This model identifies a single class, which is characterized by a relatively high intercept (*b* = 19.22 ± 2.29 SE, $p < .001$) and a modest decrease in learning scores with development (*b* = -1.33 ± 0.45 SE, $p = .004$) (Fig. S4). This class resembles the 'decrease' class of the main, 3-class model described in the main text.

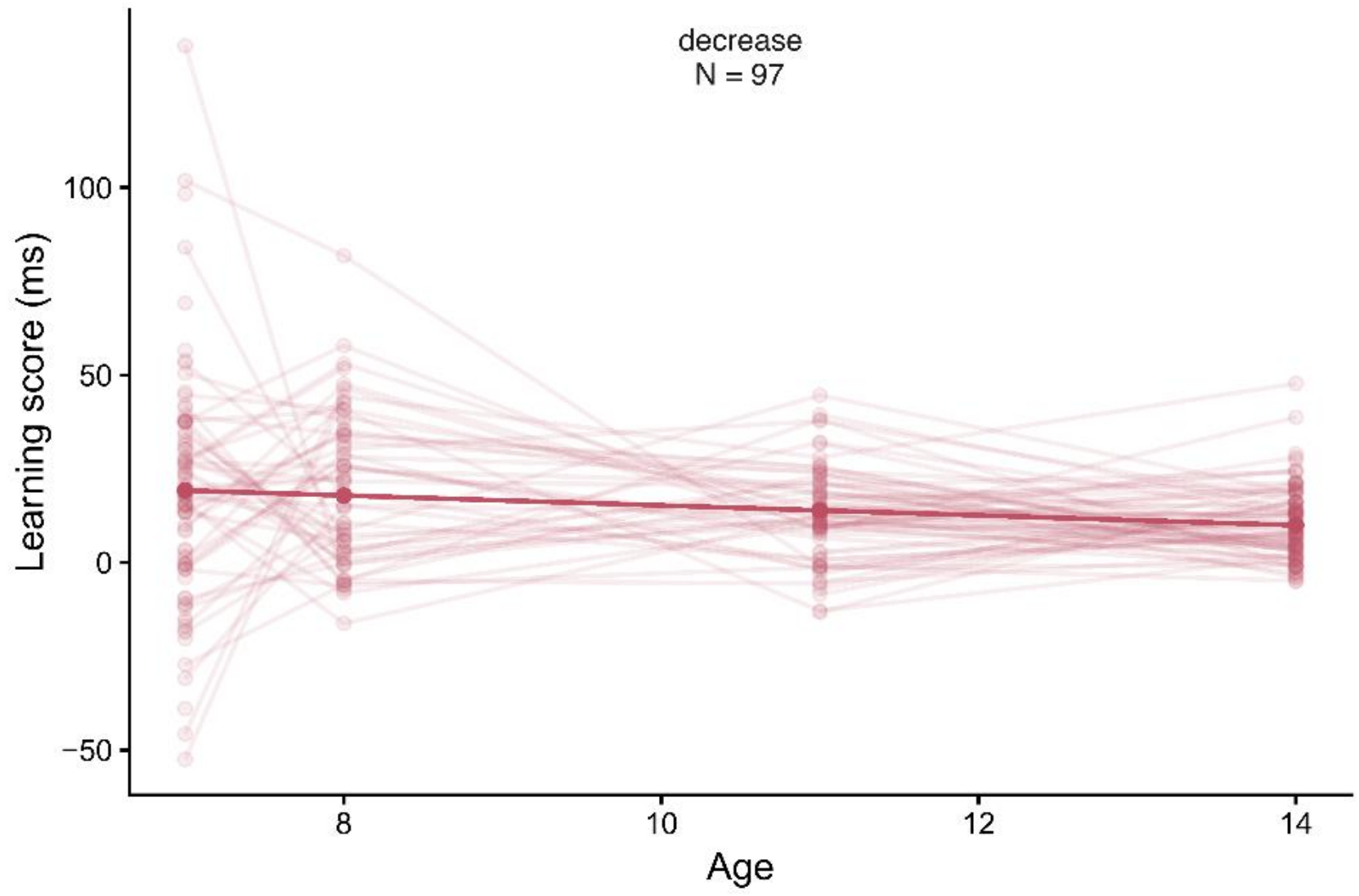

Figure S4. Results of the 1-class latent class analysis model. This model identifies a single class of participants, which on the basis of their developmental trajectory could be labelled 'decrease'. Estimated means within each class are indicated by thick lines and points, whereas actual individual trajectories of participants within each class are indicated by slightly transparent lines and points. Data are shown for N = 97 participants.

2-class model

This model identifies two extremely unbalanced classes (Fig. S5). The first class contains 3 participants, has a mean posterior probability of .96, and is characterized by a high intercept (*b* = 80.02 ± 12.97 SE, $p < .001$) and a large decrease in learning scores with development (*b* = -12.82 ± 2.64 SE, $p < .001$). This class resembles the 'high start steep decrease' class of the main, 3-class model described in the main text. The second class contains 94 participants, has a mean posterior probability of .99, and is characterized by a modest intercept (*b* = 16.89 ± 2.64 SE, $p < .001$) and a modest decrease in learning scores with development (*b* = -0.89 ± 0.42 SE, $p = .030$). This class resembles the 'decrease' class of the main, 3-class model described in the main text.

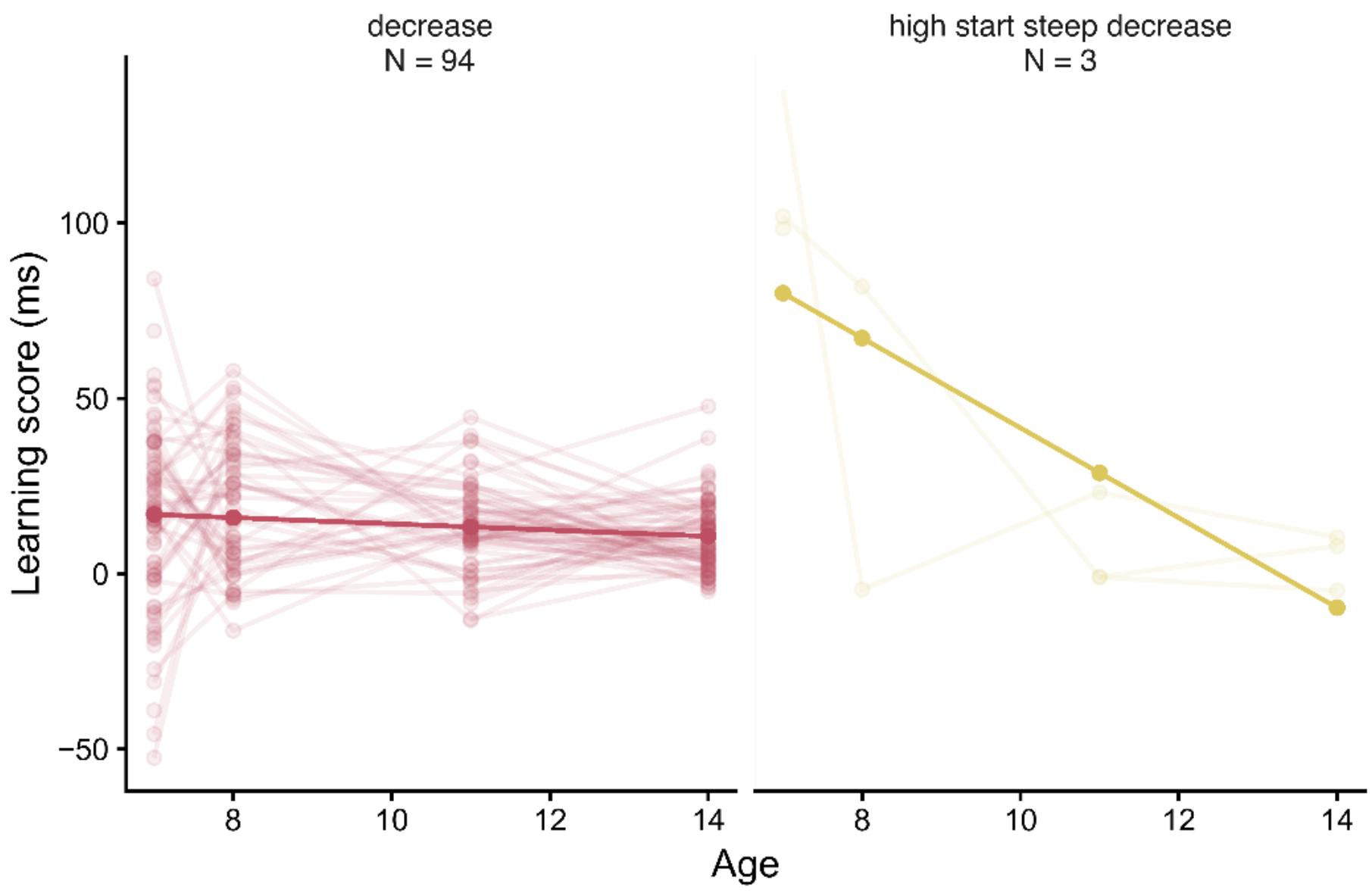

Figure S5. Results of the 2-class latent class analysis model. This model identified 2 classes of participants, which on the basis of their developmental trajectories could be labelled ‘high start steep decrease’, and ‘decrease’, with most participants being characterized by the ‘decrease’ pattern. Estimated means within each class are indicated by thick lines and points, whereas actual individual trajectories of participants within each class are indicated by slightly transparent lines and points. Data are shown for N = 97 participants.

Supplementary Note S3 – Latent class analysis robustness check

We fit a range of longitudinal clustering models spanning distinct algorithmic families, including regression-based k-means (LMKM), mixed-model–based k-means (GCKM), group-based trajectory modelling (GBTM), and growth mixture modelling with random intercept only (GMM1), and compared them to our original growth mixture model including random slopes (GMM2). We present model selection results in Table S3, and recovered trajectories in Fig. S6. We also investigated the robustness of the EF factor – Trajectory membership associations by replicating our main analysis for each sensitivity model. These are presented in Fig. S9 and S10.

Table S3. SABIC values for the different class solutions for all model types tested.

Model type	*1-class*	*2-class*	*3-class*
LMKM	551.382	381.103	269.971*
GCKM	1242.668	1087.077	946.917*
GBTM	2724.674	2696.637	2687.085*
GMM1	2726.091	2698.090	2688.503*
GMM2	2694.475	2689.649*	2691.337

Notes. Across most model types, the 3-class solution proved best fitting. Our original GMM2 model is the sole exception, with slightly better SABIC for the 2-class solution. Asterisk indicates lowest SABIC. In all cases, the 3-class model was chosen.

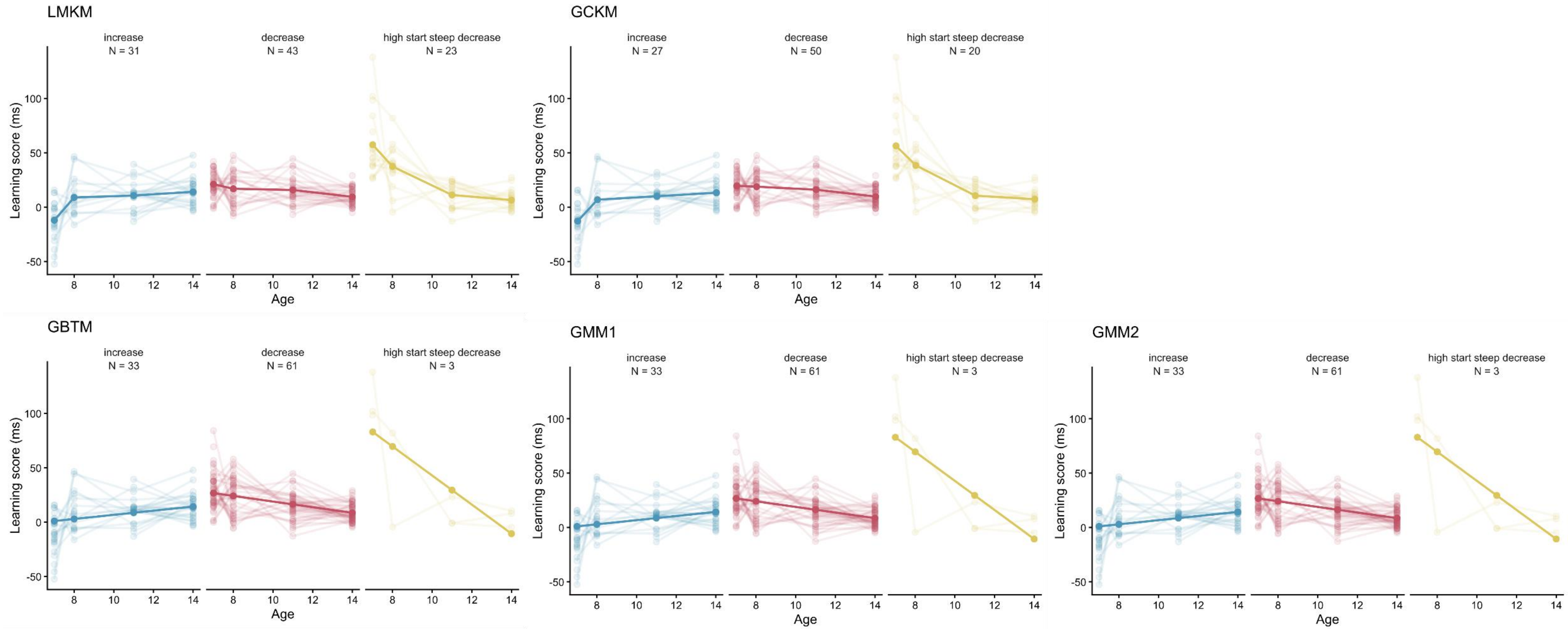

Figure S6. Recovered trajectories of best-fitting alternative longitudinal clustering models [regression-based k-means (LMKM), mixed-model–based k-means (GCKM), group-based trajectory (GBTM), growth mixture model with random intercept (GMM1)], and our original growth mixture model including random slopes (GMM2). In all panels, data are shown for N = 97 participants.

Supplementary Note S4 – Factor analysis of EF measures

We determined the factor structure of the EF measures in our dataset using maximum likelihood exploratory factor analysis (ML EFA) with varimax rotation, as implemented in the *psych* package in R[1], with default settings. The correlation matrix of EF measures is presented in Fig. S7. There are two noticeable clusters: one formed by the WM-oriented measures of the CSPAN, DSPAN, BackDSPAN, and Corsi; and one formed by the two verbal fluency components.

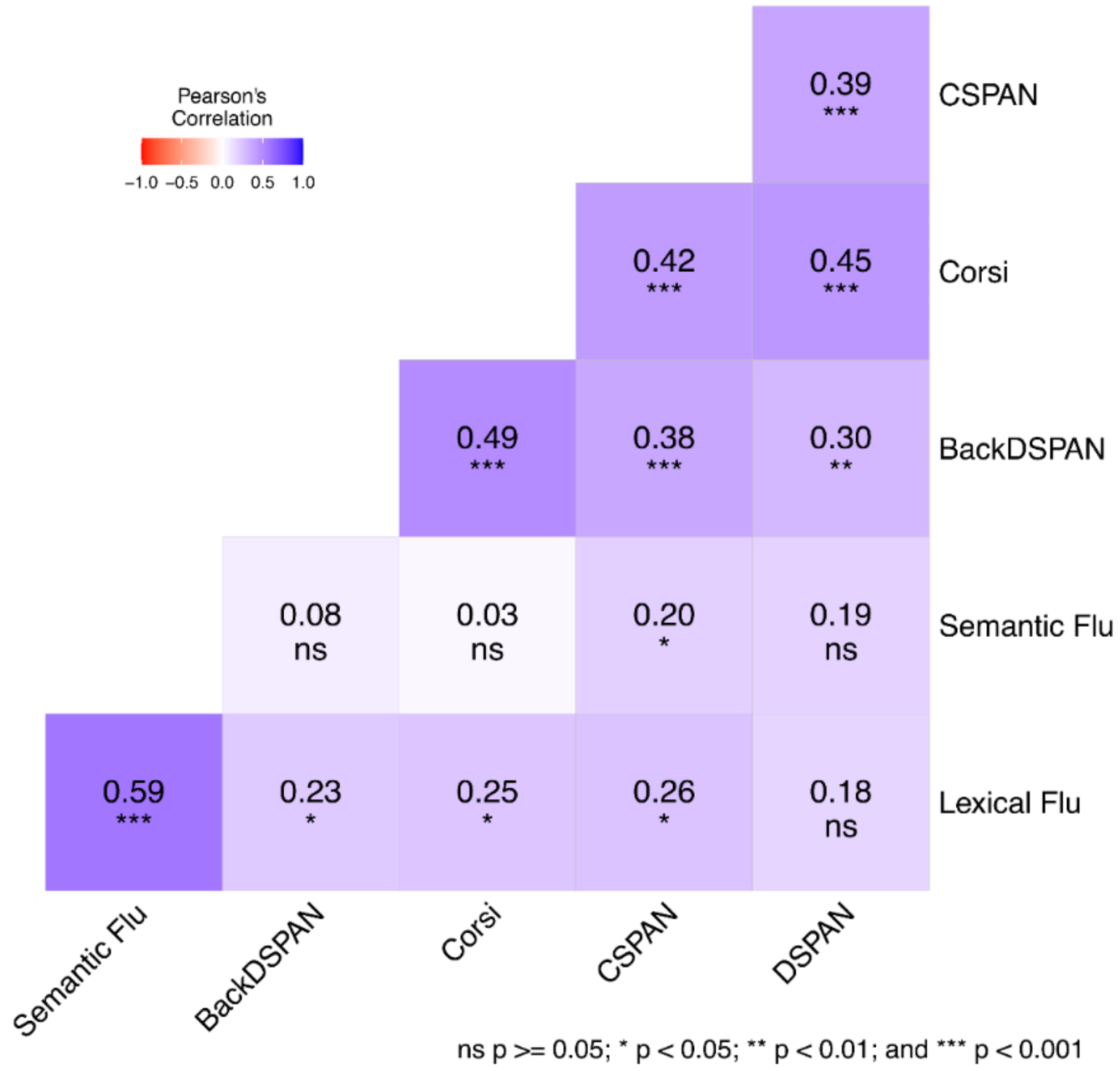

Figure S7. Bivariate Pearson's correlations between all EF measures. *P*-values are uncorrected.

To assess the factorability of the data, we utilised 3 complementary approaches[2]. Firstly, we inspected the off-diagonal elements of the anti-image covariance matrix. If the dataset is appropriate for factor analysis, these elements should be all above .50. This was the case for our dataset, with all values above .59. Secondly, we computed the Kaiser-Meyer-Olkin (KMO) test of sampling adequacy. The higher the overall KMO index, the more appropriate a factor analytic model is for the data. This value was .67, above the usual cut-off score of .60[2,3]. Finally, we performed Bartlett's test of sphericity, which tests the hypothesis that the sample correlation matrix came from a multivariate normal population in which the variables of interest are independent[4]. Rejection of the hypothesis is taken as an indication that the data are appropriate for analysis. Bartlett's test of sphericity was significant (χ^2 (15) = 131.846, $p <$ .001). Based on all three approaches, we deemed our dataset appropriate for factor analysis.

We determined the number of factors to extract using Horn's parallel analysis[5]. This approach is based on comparing the eigenvalues of factors of the observed data with those of random data from a matrix of the same size. Factors with higher eigenvalues in the observed, than in the random data are kept. We used a more stringent criterion, and compared the observed eigenvalues to the 95th percentile, instead of the mean of the simulated distributions. This analysis suggested that 2 factors were extractable (Fig. S8). Thus, the 2-factor solution was selected.

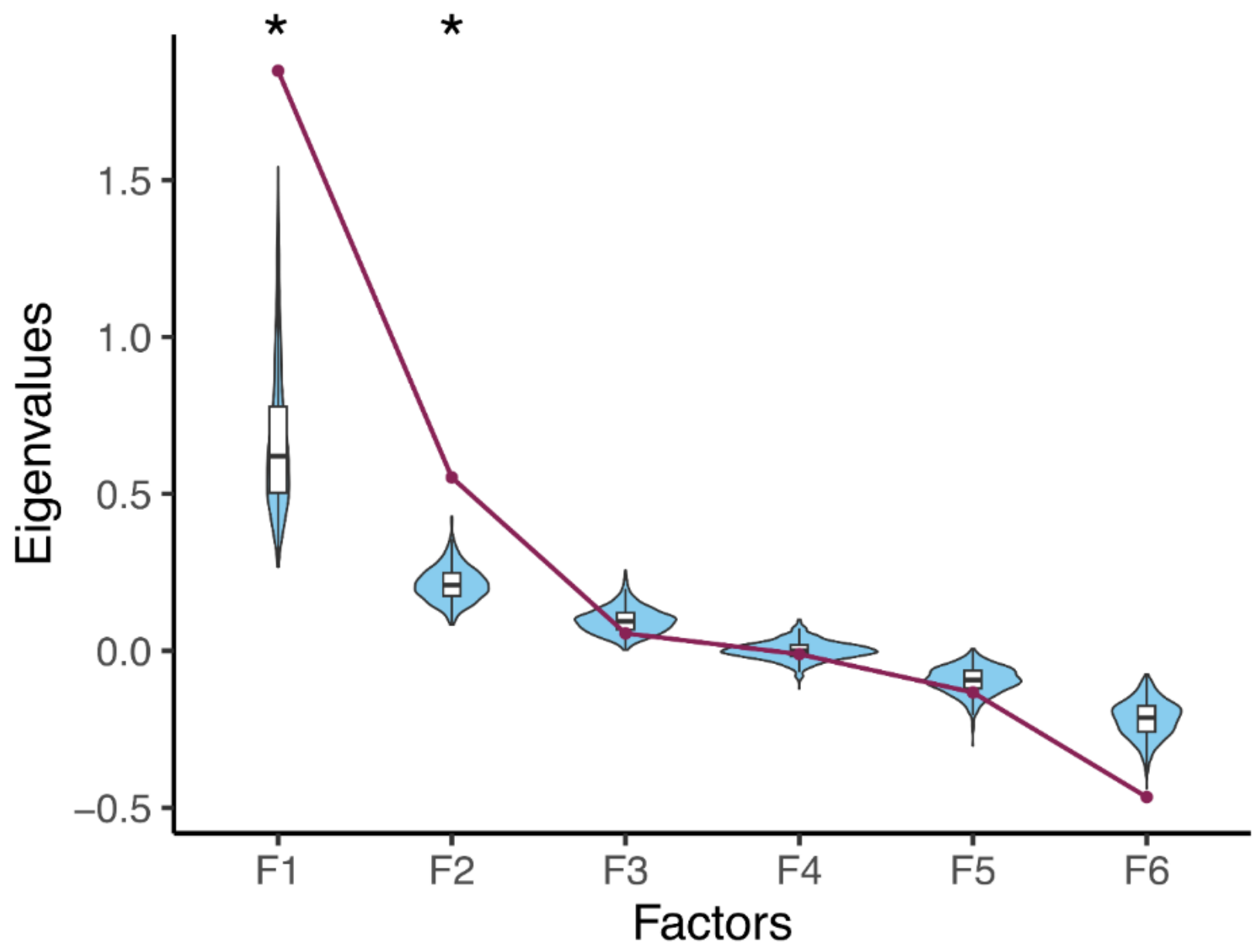

Figure S8. Parallel analysis to determine the extractable factors. The observed eigenvalues are indicated by purple dots connected by lines. These were compared to the distribution of eigenvalues obtained from simulated data, indicated by violin plots and boxplots. Stars indicate factors for which the observed eigenvalue is larger than the 95th percentile of the simulated distributions, and thus were deemed extractable.

These two factors explained 28% and 24% of the variance, respectively. The total variance explained of the model was thus 52%. The χ2 test of the null hypothesis that 2 factors are sufficient was not significant (χ^2 (4) = 2.973, p = .562), suggesting that the null can be accepted, meaning that our 2 factors are sufficient in capturing the full dimensionality of the data. Goodness of fit indices were also indicative of good fit (RMSEA = .000, 90% CI = [.000, .135]; SRMR = .028)[6]. Factor loadings are presented in Table S4. Factor 1 had high loadings from the working memory measures, namely the CSPAN, DSPAN, BackDSPAN and Corsi tasks. Factor 2 had high loadings from the two fluency measures. Thus, we tentatively call Factor 1 the WM, and Factor 2 the Fluency factors. We proceeded to calculate factor scores for each participant for both factors, using Thomson's[7] method, and used these scores as predictors of latent class membership.

Table S4. Results of the 2-factor solution.

	Factor 1 WM	Factor 2 Fluency	Communality
CSPAN	.56*	.19	.35
DSPAN	.54*	.18	.33
BackDSPAN	.61*	.07	.38
Corsi	.80*	.02	.64
Lexical fluency	.27	.59*	.42
Semantic fluency	.01	.99*	.99

Notes. Factor loadings and communalities of each EF measure on the 2 factors, based on the 2 factor varimax rotated ML solution (53% of variance explained). Loadings above the threshold of .30 are marked with an asterisk to aid interpretation.

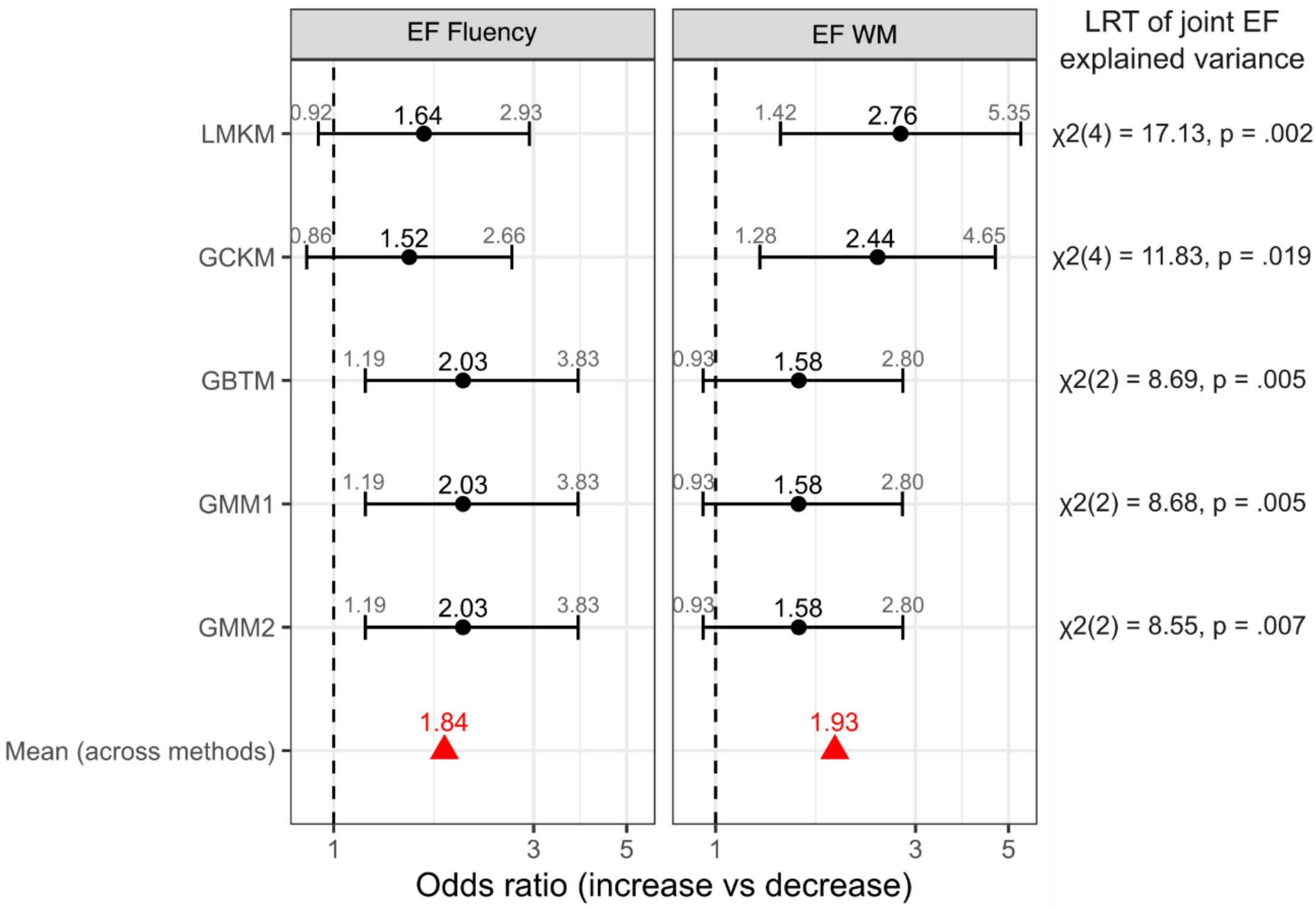

Figure S9. Odds ratios (increase vs decrease) of EF effects across longitudinal clustering models. Across all models, participants were less likely to be classified in the ‘increase' trajectory subgroup and more likely to be classified in the ‘decrease' trajectory subgroup with increasing EF scores. The joint effect of both EF scores was also statistically significant in each model, according to LRT tests. Note that even though the GBTM, GMM1 and GMM2 models recovered the exact same classification, their posterior probabilities differ, which is why these probability-weighted regressions can also be slightly different.

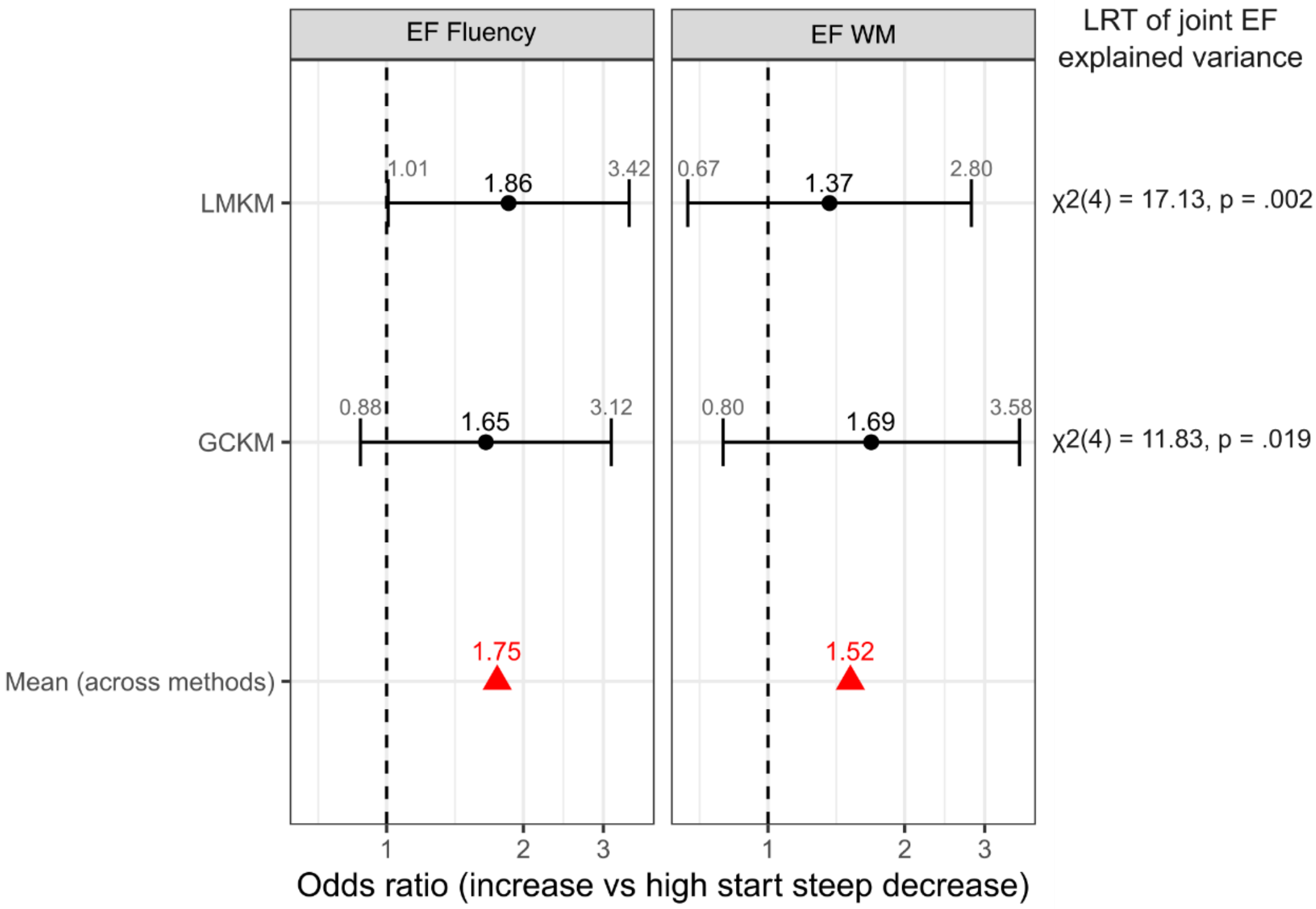

Figure S10. Odds ratios (increase vs high start steep decrease) of EF effects across longitudinal clustering models. Only two approaches (LMKM and GCKM) resulted in a high start steep decrease class with enough participants for this analysis.

Supplementary Note S5 – Repeated measures correlations on the whole ASRT task

In the main manuscript, we conducted repeated measures correlations to test whether lower RTs were systematically associated with lower statistical learning scores. For completeness, here, we present the results from the entire task (all 20 blocks).

This analysis revealed a statistically significant, weak negative relationship at age 8, 11 and 14, but not at age 7 (Age 7: $r_{rm}(1595) = 0.041$, 95% CI = [-0.008, 0.090], $p = .103$, $BF_{01} = 3.85$; Age 8: $r_{rm}(1155) = -0.079$, 95% CI = [-0.136, -0.021], $p = .007$, $BF_{01} = 0.50$; Age 11: $r_{rm}(1270) = -0.055$, 95% CI = [-0.110, 0.000], $p = .049$, $BF_{01} = 2.50$; Age 14: $r_{rm}(1819) = -0.079$, 95% CI = [-0.125, -0.034], $p < .001$, $BF_{01} = 0.07$). At ages 8, 11, and 14, faster blocks were modestly associated with higher learning scores, the opposite of the pattern that would be expected if lower RTs artificially suppressed learning. These negative correlations most likely reflect the confounding influence of ongoing learning: as participants become increasingly faster on high-probability compared to low-probability triplets (indicating stronger learning), they also become faster overall. This joint improvement creates a spurious negative correlation between RTs and learning scores when early blocks are included. Overall, the whole-task results presented here support our decision to restrict the within-participant correlation analyses to the second half of the task, where the influence of such confounds is reduced.

Supplementary Note S6 - The effects of RT transformation and correlations between reaction time variability and learning

Although transforming reaction times is a common approach to control for baseline RT differences, we chose not to apply it, as transformation can alter RT variability and potentially obscure meaningful information. In more detail, if effects are scale-dependent, altering the scale properties of a distribution can cause meaningful effects to disappear or introduce spurious effects[10]. As Lo and Andrews[11] argue, since most theories in chronometric cognitive psychological research have been based on raw RTs, analyses should be conducted on this original scale whenever possible. Second, transformations can alter RT variability, potentially obscuring valuable information – particularly if the relationship between RT variability and learning varies with age. To investigate this issue, within each age group, we calculated the within-subject coefficient of variation of reaction times (RTCV) for the first block and the entire task and correlated these scores with whole task learning scores. Our findings revealed that whole task RT variability was positively correlated with learning scores uniquely at age 14 (Fig. S11). This suggests that higher initial and overall variability in response times may be related to more efficient learning, with this relationship emerging during development. We illustrate the drastic changes to the distribution of overall RT due to transformation in Fig. S12. Specifically, we display the distribution of raw, unstandardized RTs alongside two commonly used transformation methods. The first method is a ratio-based transformation, in which median RTs for high- and low probability triplets in each block are divided by the participant's median RT (irrespective of triplet identity) from the first block of that session (e.g., [12]). The second involves a Z-score transformation applied separately for each participant and age group (e.g., [13]). As shown, both transformations homogenize the central tendency of the distributions–as intended effect–but also significantly distort the scale, shape, and variability of the distributions. Given these limitations, we opted to analyse raw RTs using linear mixed models with a random effects structure that included random, participant- and age-specific uncorrelated intercepts and slopes for the Block factor, providing a more faithful representation of individual and developmental variability in statistical learning.

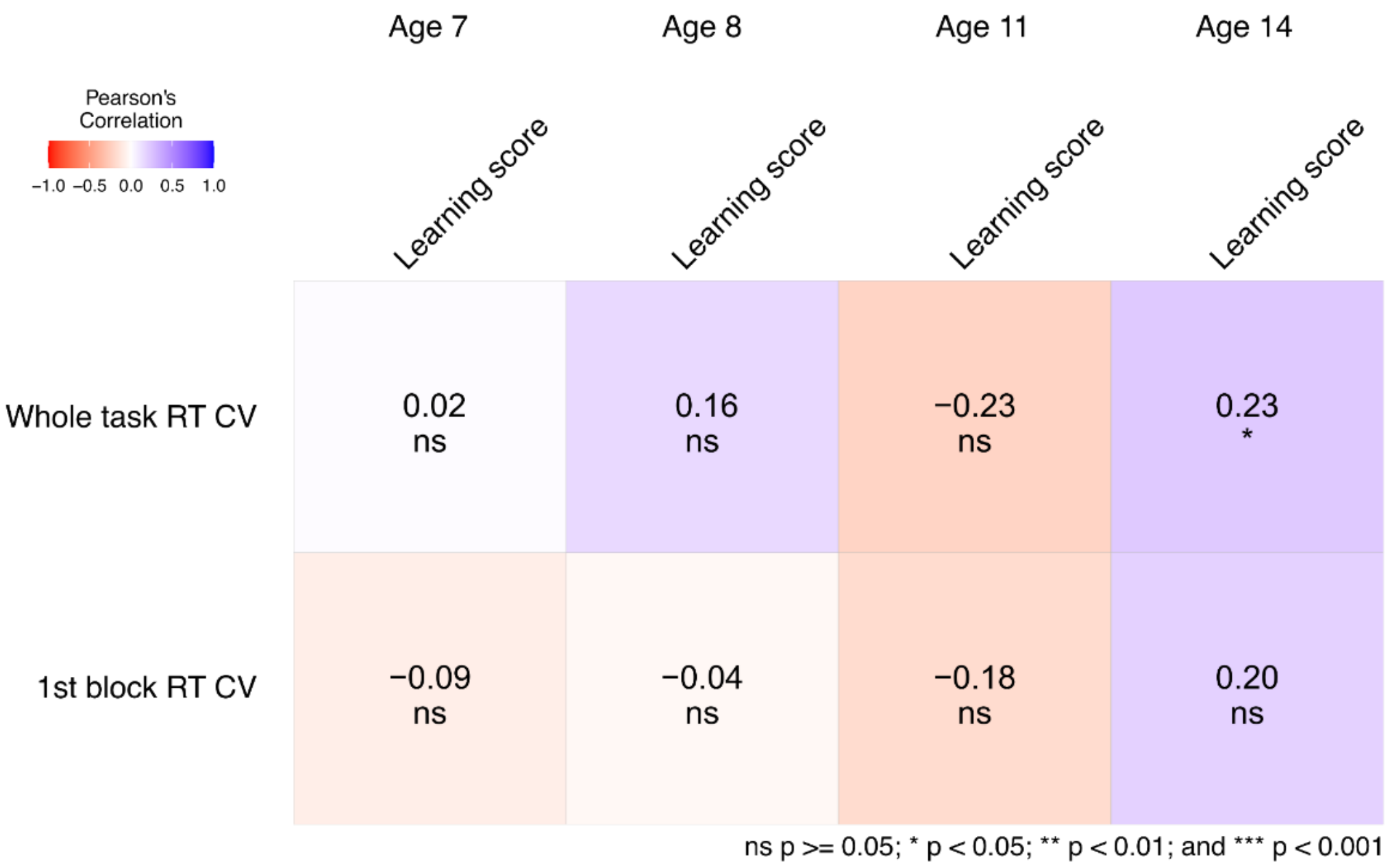

Figure S11. Correlations between reaction time variability and learning. For each age group, the correlation between 1st block and whole task RT variability (measured by the coefficient of variation [CV] of RTs) and whole task learning scores is shown. Relationships between response variability and overall learning were inconsistent across age groups. A statistically significant relationship emerged between whole task RT variability and learning at age 14. P values are uncorrected.

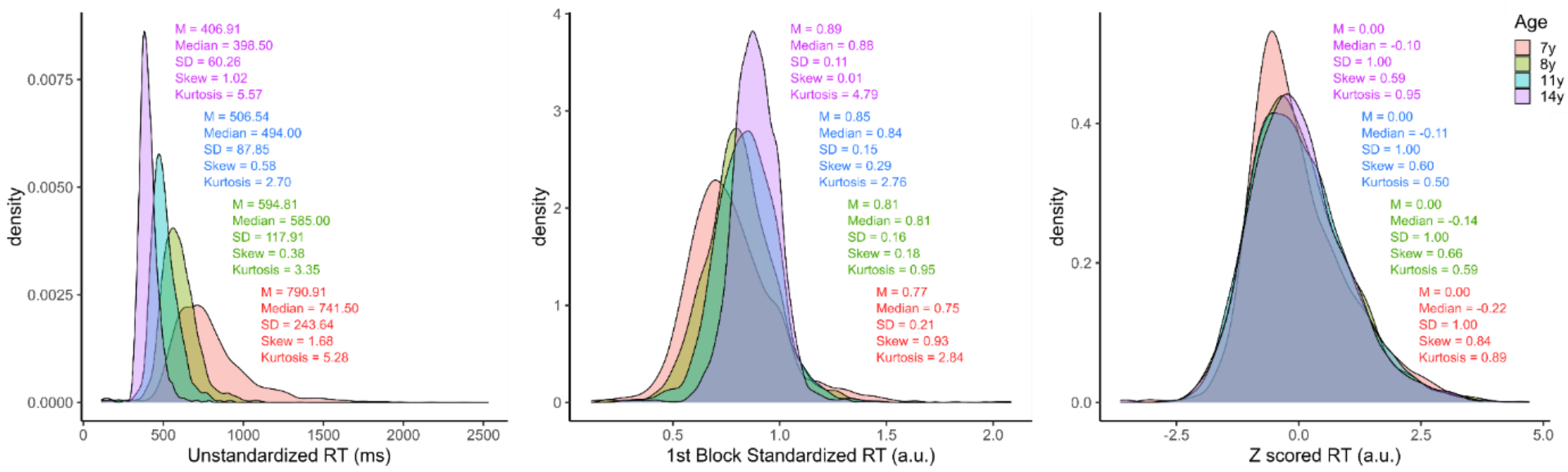

Figure S12. Distribution of unstandardized (left), 1st block standardized (middle) and z-transformed (right) reaction times across the four ages, along with summary statistics of each distribution. The Y axis represents the kernel density estimate from the geom_density function of the ggplot2 R package. As shown, the transformations not only homogenize the central tendency of the distributions–as intended–but also substantially alter their shape and variability.

Supplementary Note S7 – Sensitivity analysis with exclusion of participants with repeated sequences

Developmental trajectories of statistical learning

We repeated the main LMM on the reduced sample that excluded the 17 participants who were assigned the same sequence on at least 2 assessments, leading to a sample size of 80. Block wise median reaction time was used as the outcome variable in a linear mixed model including Triplet type (high- vs. low-probability), Block (Block 1-20), and Age (7, 8, 11 or 14), as well as all their higher order interactions as fixed effects, and uncorrelated by-participant intercepts and slopes for Block, Age, and their interaction as random effects.

Model results are summarized in Table S5 and S6. All effects described in the main text proved to be robust. There was a significant main effect of Block ($F_{(1,74.26)}$ = 242.17, p < .001), indicating decreasing overall reaction times throughout the task (b = -7.96, 95% CI = [-8.98, -6.94]). The main effect of Age was also significant ($F_{(3,90.93)}$ = 120.36, p < .001), indicating decreasing overall reaction times during development (Age 7: 789 ms, 95% CI = [759, 819]; Age 8: 597 ms, 95% CI = [574, 620]; Age 11: 505 ms, 95% CI = [485, 526]; Age 14: 406 ms, 95% CI = [373, 439]; with each pairwise comparison being p < .001). There was a significant Age × Block interaction as well ($F_{(3,79.61)}$ = 16.19, p < .001). Post hoc estimated marginal trends revealed that the slope of Block progressively became less steep with development (Age 7: b = -13.02, 95% CI = [-15.04, -11.01]; Age 8: b = -9.66, 95% CI = [-11.31, -8.01]; Age 11: b = -6.55, 95% CI = [-7.91, -5.19]; Age 14: b = -2.60, 95% CI = [-4.78, -0.42]; each pairwise comparison being significant with p < .019). This effect likely stemmed from the generally faster starting reaction times, leaving less room to improve at later ages at the group level.

The model resulted in a significant main effect of Triplet type ($F_{(1,9577.87)}$ = 108.47, p < .001), indicating faster reaction times for high-probability triplets than for low-probability triplets (high: 566 ms, 95% CI = [550, 582]; low: 583 ms, 95% CI = [567, 599]). This effect indicates that on average, participants engaged in statistical learning. The Block × Triplet type interaction was also significant ($F_{(1,9577.92)}$ = 6.26, p = .012), stemming from a steeper slope for high-probability (b = -8.32, 95% CI = [-9.38, -7.26]), than low-probability triplets (b = -7.60, 95% CI = [-8.65, -6.54]), indicating increasing statistical learning with time within task. There was a significant Age × Triplet type interaction ($F_{(3,9577.87)}$ = 4.20, p = .006). Learning scores indicated decreasing statistical learning during development (Age 7: 24.49 ms, 95% CI = [18.35, 30.60]; Age 8: 20.01 ms, 95% CI = [12.65, 27.40]; Age 11: 14.98 ms, 95% CI = [8.23, 21.70]; Age 14: 9.88 ms, 95% CI = [4.15, 15.60]; with age 14 learning scores being significantly lower than age 7 (p < .001) and 8 (p = .034), and age 11 learning scores being lower than age 7 (p = .041)).

Table S5. Type III test of effects of the linear mixed model, predicting block-wise median reaction times.

**Type III test of effects**	*F*	*df*	*p*
Age	120.36	3,90.93	< .001*
Block	242.17	1, 74.26	< .001*
Triplet type	108.47	1, 9577.87	< .001*
Age × Block	16.19	3, 79.61	< .001*
Age × Triplet type	4.20	3, 9577.87	.006*
Block × Triplet type	6.26	1, 9577.92	.012*
Age × Block × Triplet type	0.38	3, 9577.92	.974

Notes. Significant terms are marked with an asterisk. All *p* values are two-sided and unadjusted for multiple comparisons.

Table S6. Fixed and random effects of the linear mixed model, predicting block-wise median reaction times.

**Fixed effects**	*b*	*SE b*	*95% CI*	*t*	*df*	*p*
(Intercept)	574.39	7.90	558.64 – 590.13	72.67	75.97	< .001*
Age [7y]	214.47	13.08	188.45 – 240.48	16.39	83.98	< .001*
Age [8y]	22.86	8.06	6.76 – 38.96	2.84	61.86	.006*
Age [11y]	-68.94	6.77	-82.48 – -55.41	-10.18	62.34	< .001*
Block	-7.96	0.51	-8.98 – -6.94	-15.56	74.26	< .001*
Triplet type [Low]	8.67	0.83	7.04 – 10.30	10.41	9577.87	< .001*
Age [7y] × Block	-5.07	0.89	-6.83 – -3.30	-5.72	84.70	< .001*
Age [8y] × Block	-1.70	0.61	-2.91 – -0.49	-2.80	70.43	.007*
Age [11y] × Block	1.41	0.46	0.49 – 2.33	3.06	64.43	.003*
Age [7y] × Triplet type [Low]	3.57	1.39	0.86 – 6.29	2.58	9577.76	.010*
Age [8y] × Triplet type [Low]	1.34	1.57	-1.74 – 4.41	0.85	9577.85	.394
Age [11y] × Triplet type [Low]	-1.18	1.48	-4.07 – 1.71	-0.80	9578.04	.424
Block × Triplet [Low]	0.36	0.14	0.08 – 0.64	2.50	9577.92	.012*
Age [7y] × Block × Triplet type [Low]	-0.05	0.24	-0.52 – 0.42	-0.21	9577.78	.834
Age [8y] × Block × Triplet type [Low]	0.03	0.27	-0.50 – 0.57	0.12	9577.81	.905
Age [11y] × Block × Triplet type [Low]	0.09	0.26	-0.41 – 0.59	0.36	9578.22	.718

**Random Effects**	
σ^2	6765.68 (82.25)
$\tau_{00\ \text{Participant}}$	4534.58 (67.34)
$\tau_{11\ \text{Block}}$	17.50 (4.18)
$\tau_{11\ \text{Age[7y]}}$	12271.90 (110.78)
$\tau_{11\ \text{Age[8y]}}$	3003.38 (54.80)
$\tau_{11\ \text{Age[11y]}}$	2291.25 (47.87)
$\tau_{11\ \text{Block × Age[7y]}}$	52.78 (7.27)
$\tau_{11\ \text{Block × Age[8y]}}$	14.37 (3.79)
$\tau_{11\ \text{Block × Age[11y]}}$	7.15 (2.67)
ICC	0.40
$N_{\text{Participant}}$	80
Observations	10113
Marginal R^2 / Conditional R^2	0.690 / 0.814

Notes. The table shows regression coefficients of fixed effects and summary information about the random effects. Random effects are presented as variance and SD in brackets. The marginal R-squared considers only the variance of the fixed effects, while the conditional R-squared takes both the fixed and random effects into account (based on Nakagawa et al.[9]). Degrees of freedom are based on Satterthwaite's approximation. Significant terms are marked with an asterisk. All p values are two-sided and unadjusted for multiple comparisons. Terms in brackets indicate the level of the factor that is contrasted against the reference level (High triplets for Triplet type and Age 14 for Age). Because we were interested in lower order effects too, both Age and Triplet type were effects coded, therefore lower order interactions and main effects are to be interpreted as deviations from the grand mean value of the dataset (at age 10 in our case, even though this value was not practically observed, and at the average of high- and low-triplets).
Model equation in *lmer* syntax: RT ~ Age*Block*Triplet type + (Block*Age || Participant)

Subgroups with different developmental trajectories of statistical learning

We similarly repeated the LCA model on this reduced sample. The three-class model once again provided the best trade-off between goodness of fit, class balance and classification performance (Table S7). Although the three-class model had the highest SABIC (SABIC = 2190.850), it resulted in relatively balanced classes (class 1 = 77.50 %; class 2 = 2.50 %; class 3 = 20.00 %). Mean posterior classification probabilities were also adequate in each class ($p_{(class1)}$ = .86; $p_{(class2)}$ = .99; $p_{(class3)}$ = .84).

Table S7. Results of the Latent Class Mixed Models.

Model	*AIC*	*SABIC*	*Entropy*	*class1 (N,%)*	*class2 (N,%)*	*class3 (N,%)*
1-class	2194.518*	2189.890	1.00	80 (100%)	-	-
2-class	2196.217	2189.275*	0.95	2 (2.50%)	78 (97.50%)	-
3-class*	2200.106	2190.850	0.70	56 (70.00%)	2 (2.50%)	22 (27.50%)

Notes. The AIC and SABIC are shown as goodness of fit indices, the entropy characterizes the classification performance, with higher values indicating that individuals can be classified with more confidence. Sizes of classes (in absolute number, and relative percentage of participants) are also reported. Asterisks indicate which model is the best fitting according to the fit index. The solution that provided the best trade-off between goodness of fit, class balance, and classification performance is the 3-class model. AIC = Akaike information criterion; SABIC = Sample size adjusted Bayesian information criterion.

The resulting classes were similar to the class identified in the model in the main text. The first class included the largest number of participants (N = 56). It was characterized by a relatively high intercept (b = 27.30 ± 3.33 SE, p < .001) and a modest decrease in learning scores with development (b = -2.78 ± 0.65 SE, p < .001). This class resembles the 'decrease' class of the main model. The second class had the lowest number of participants (N = 2). It was characterized by an extremely high intercept (b = 81.73 ± 9.74 SE, p < .001) and a large decrease in learning scores with development (b = -12.62 ± 2.16 SE, p < .001). This class resembles the 'high start, steep decrease' class of the main model. The third and final class included about one fourth of participants (N = 22). It was characterized by a relatively low intercept (b = -1.03 ± 5.77 SE, p = .858) and a small increase in learning scores with development (b = 1.93 ± 1.01 SE, p = .056). This class resembles the 'increase' class of the main model. Once again, most participants showed a decreasing trend in their statistical learning scores with development.

Executive functions predicting subgroup membership

We also replicated the logistic regression, with class membership weighted by the posterior probabilities as outcomes, and Fluency and WM latent executive function factor scores as predictors, and the reference class set to the ‘increase’ class described above.

The effects of the EF factor scores were replicated. As Fluency scores increased, participants were more likely to be classified in the ‘decrease' than in the ‘increase' trajectory profile (OR = 2.03, 95% CI = [1.08, 4.14], p = .040). As WM scores increased, participants were also more likely to be classified in the ‘decrease' than in the ‘increase' trajectory profile, but this was not statistically significant (OR = 1.61, 95% CI = [0.85, 3.32], p = .169).

Supplementary References

1. Revelle, W. psych: Procedures for Psychological, Psychometric, and Personality Research. Northwestern University (2022).
2. Dziuban, C. D. & Shirkey, E. C. When is a correlation matrix appropriate for factor analysis? Some decision rules. *Psychol. Bull.* **81**, 358–361 (1974).
3. Kaiser, H. F. A second generation little jiffy. *Psychometrika* **35**, 401–415 (1970).
4. Bartlett, M. S. Tests of significance in factor analysis. *Br. J. Stat. Psychol.* **3**, 77–85 (1950).
5. Horn. A rationale and test for the number of factors in factor analysis. *Psychometrika* **30**, 179–185 (1965).
6. Hu, L. & Bentler, P. M. Cutoff criteria for fit indexes in covariance structure analysis: Conventional criteria versus new alternatives. *Struct. Equ. Model.* **6**, 1–55 (1999).
7. Thomson, G. H. *The Factorial Analysis of Human Ability*. (Houghton Mifflin, Boston, 1939).
8. Morey, R. D. Confidence Intervals from Normalized Data: A correction to Cousineau (2005). *Tutor. Quant. Methods Psychol.* **4**, 61–64 (2008).
9. Nakagawa, S., Johnson, P. C. D. & Schielzeth, H. The coefficient of determination R2 and intra-class correlation coefficient from generalized linear mixed-effects models revisited and expanded. *J. R. Soc. Interface* **14**, (2017).
10. Loftus, G. R. On interpretation of interactions. *Mem. Cognit.* **6**, 312–319 (1978).
11. Lo, S. & Andrews, S. To transform or not to transform: using generalized linear mixed models to analyse reaction time data. *Front. Psychol.* **6**, (2015).
12. Tóth-Fáber, E., Janacsek, K. & Németh, D. Statistical and sequence learning lead to persistent memory in children after a one-year offline period. *Sci. Rep.* **11**, 12418 (2021).
13. Janacsek, K., Fiser, J. & Nemeth, D. The best time to acquire new skills: age-related differences in implicit sequence learning across the human lifespan: Implicit learning across human lifespan. *Dev. Sci.* **15**, 496–505 (2012).
