# Supplementary material for: Longitudinal evidence for decreasing statistical learning abilities across childhood
Source: Nat Commun. 2026 Sep 3;17:10471. doi: 10.1038/s41467-026-77261-1 (PMC13635216; doi:10.1038/s41467-026-77261-1)
Supplement: Supplementary file 2 [file 41467_2026_77261_MOESM2_ESM.pdf]

nature portfolio

Corresponding author(s): Dezso Nemeth

Last updated by author(s): Jul 30, 2026

Reporting Summary

Nature Portfolio wishes to improve the reproducibility of the work that we publish. This form provides structure for consistency and transparency in reporting. For further information on Nature Portfolio policies, see our Editorial Policies and the Editorial Policy Checklist.

Statistics

For all statistical analyses, confirm that the following items are present in the figure legend, table legend, main text, or Methods section.

n/a	Confirmed	
☐	☒	The exact sample size (n) for each experimental group/condition, given as a discrete number and unit of measurement
☐	☒	A statement on whether measurements were taken from distinct samples or whether the same sample was measured repeatedly
☐	☒	The statistical test(s) used AND whether they are one- or two-sided<br>*Only common tests should be described solely by name; describe more complex techniques in the Methods section.*
☐	☒	A description of all covariates tested
☐	☒	A description of any assumptions or corrections, such as tests of normality and adjustment for multiple comparisons
☐	☒	A full description of the statistical parameters including central tendency (e.g. means) or other basic estimates (e.g. regression coefficient) AND variation (e.g. standard deviation) or associated estimates of uncertainty (e.g. confidence intervals)
☐	☒	For null hypothesis testing, the test statistic (e.g. F, t, r) with confidence intervals, effect sizes, degrees of freedom and P value noted<br>*Give P values as exact values whenever suitable.*
☐	☒	For Bayesian analysis, information on the choice of priors and Markov chain Monte Carlo settings
☐	☒	For hierarchical and complex designs, identification of the appropriate level for tests and full reporting of outcomes
☐	☒	Estimates of effect sizes (e.g. Cohen's d, Pearson's r), indicating how they were calculated

Our web collection on statistics for biologists contains articles on many of the points above.

Software and code

Policy information about availability of computer code

Data collection	Custom-written E-Prime code was used.
Data analysis	Statistical analysis was performed in R 4.2.1.

For manuscripts utilizing custom algorithms or software that are central to the research but not yet described in published literature, software must be made available to editors and reviewers. We strongly encourage code deposition in a community repository (e.g. GitHub). See the Nature Portfolio guidelines for submitting code & software for further information.

Data

Policy information about availability of data

All manuscripts must include a data availability statement. This statement should provide the following information, where applicable:

- Accession codes, unique identifiers, or web links for publicly available datasets
- A description of any restrictions on data availability
- For clinical datasets or third party data, please ensure that the statement adheres to our policy

All data reported in this study and the codes for data analyses are available to download via https://doi.org/10.17605/OSF.IO/GRBD4.

Research involving human participants, their data, or biological material

Policy information about studies with human participants or human data. See also policy information about sex, gender (identity/presentation), and sexual orientation and race, ethnicity and racism.

Reporting on sex and gender	Sex was determined based on self-reporting. Sex- or gender-based analyses were not performed.
Reporting on race, ethnicity, or other socially relevant groupings	Such data was not used.
Population characteristics	The research sample consisted of healthy participants who were tested four times: at age 7, 8, 11 and 14 years (N = 135).
Recruitment	Primary schools were approached to request participation in the study.
Ethics oversight	All experimental procedures were approved by the research ethics committee of Eötvös Loránd University, Budapest, Hungary, and was conducted in accordance with the Declaration of Helsinki.

Note that full information on the approval of the study protocol must also be provided in the manuscript.

Field-specific reporting

Please select the one below that is the best fit for your research. If you are not sure, read the appropriate sections before making your selection.

☐ Life sciences ☒ Behavioural & social sciences ☐ Ecological, evolutionary & environmental sciences

For a reference copy of the document with all sections, see nature.com/documents/nr-reporting-summary-flat.pdf

Behavioural & social sciences study design

All studies must disclose on these points even when the disclosure is negative.

Study description	Quantitative study assessing reaction times and response accuracy. The aim of the study was to explore the development of statistical learning in a longitudinal design.
Research sample	The research sample consisted of healthy participants who were tested four times: at age 7, 8, 11 and 14 years (N = 135). All participants had normal or corrected to normal vision and hearing and none of them had any neurological, psychiatric, or neurodevelopmental disorder.
Sampling strategy	Primary schools were approached to request participation in the study. All participation was completely voluntary. The sample size was determined based on previous studies using the same task.
Data collection	The data were recorded with a notebook. Participants attended the experiment alone, only the participant and the experimenter were present during the experiment. All experimenters were blind to the exact hypothesis of the study.
Timing	Data collection took place from 2010 to 2018.
Data exclusions	A total of 135 participants were initially recruited for the study. Given that our primary focus was the longitudinal, within-subject development of statistical learning, we excluded participants who did not complete at least two of the four assessments (24 participants). From this, we further excluded participants with missing data on any of the neuropsychological tests (14 participants). This resulted in a final sample size of 97 participants.
Non-participation	24 participants completed only one out of the four assessments regarding our main task.
Randomization	Participants were not allocated into experimental groups.

Reporting for specific materials, systems and methods

We require information from authors about some types of materials, experimental systems and methods used in many studies. Here, indicate whether each material, system or method listed is relevant to your study. If you are not sure if a list item applies to your research, read the appropriate section before selecting a response.

Materials & experimental systems

n/a	Involved in the study
☒	☐ Antibodies
☒	☐ Eukaryotic cell lines
☒	☐ Palaeontology and archaeology
☒	☐ Animals and other organisms
☒	☐ Clinical data
☒	☐ Dual use research of concern
☒	☐ Plants

Methods

n/a	Involved in the study
☒	☐ ChIP-seq
☒	☐ Flow cytometry
☒	☐ MRI-based neuroimaging

Plants

Seed stocks	*Report on the source of all seed stocks or other plant material used. If applicable, state the seed stock centre and catalogue number. If plant specimens were collected from the field, describe the collection location, date and sampling procedures.*
Novel plant genotypes	*Describe the methods by which all novel plant genotypes were produced. This includes those generated by transgenic approaches, gene editing, chemical/radiation-based mutagenesis and hybridization. For transgenic lines, describe the transformation method, the number of independent lines analyzed and the generation upon which experiments were performed. For gene-edited lines, describe the editor used, the endogenous sequence targeted for editing, the targeting guide RNA sequence (if applicable) and how the editor was applied.*
Authentication	*Describe any authentication procedures for each seed stock used or novel genotype generated. Describe any experiments used to assess the effect of a mutation and, where applicable, how potential secondary effects (e.g. second site T-DNA insertions, mosiacism, off-target gene editing) were examined.*
