# Supplementary material for: Longitudinal evidence for decreasing statistical learning abilities across childhood
Source: Nat Commun. 2026 Sep 3;17:10471. doi: 10.1038/s41467-026-77261-1 (PMC13635216; doi:10.1038/s41467-026-77261-1)
Supplement: Supplementary file 3 [file 41467_2026_77261_MOESM3_ESM.pdf]

nature portfolio

Peer Review File

Longitudinal evidence for decreasing statistical learning abilities across childhood

Corresponding Author: Professor Dezső Németh

This file contains all reviewer reports in order by version, followed by all author rebuttals in order by version.

Version 0:

Reviewer comments:

Reviewer #1

(Remarks to the Author)
The authors of 'Longitudinal evidence for decreasing statistical learning abilities across childhood' present data from an ASRT task at four longitudinal timepoints, and a battery of executive functon(EF) tasks at the 4th time-point. They then measure the difference in response times to the targets which are easier versus harder to predict based on the statistical structure of a spatial sequence. The main findings are that participants 'statistical learning score' (operationalized as the difference in reaction time to hard to predict targets and easy to predict targets) goes down across developmental time, and that participants who show better EF at time-point 4 are more likely to have shown this developmental trajectory. I commend the authors extensive longitudinal data collection and attempt to link developmental trajectories in statistical learning to other cognitive processes—these questions are timely and of interest to developmental and cognitive psychologists. I have some reservations about the authors primary claims, based off the way they operationalized their statistical learning score. I detail these and other questions below.

1. My primary concern is about how the authors chose to operationalize a 'statistical learning score' as the difference between reaction times to high probability trials (i.e. targets at the end of a high-probability triplet) and low probability trials (i.e. targets at the end of low-probability triplets). The youngest children show the biggest difference in these trials, while the oldest participants show the smallest difference. My concern is that the older participants are essentially at ceiling in their reaction times. This is supported by the finding that there is less of a decrease in reaction times across blocks in the older participants (which the authors explain as "likely stemm[ing] from faster initial reaction times, leaving less room to improve at later ages."), and leads to the possibility that the small difference in RTs to high and low probability targets is because the older participants are already responding very quickly to both types of trials. This would mean the authors have minimized their chances to observe any difference in the reaction time to high and low probability targets in the older groups (and might help explain why the accuracy results presented in the supplement show a different pattern). Figure 1a also makes it look like the error bars between the low and high probability targets are less overlapping in older participants than younger participants, suggesting that the difference in responses to these trial types might actually be more distinct in older groups.
2. Relatedly, could this smaller subtraction score also imply that older participants are doing relatively better on the less predictable trials than younger participants? I understand from the authors' citations that previous reports of statistical learning using this task have used the difference between response times to high and low probability targets, but much of the ongoing work studying developmental differences in statistical learning is acknowledging that what learners of different ages might be learning about their statistical experiences is different. One possibility is that the younger ages are pretty good at learning about the strong predictive relationships, but struggle with the less predictive ones, while older children are able to also learn the less common relationships in the low-probability triplets—this could also lead to a smaller difference in the reaction times across these trial types with age. It will be important to more carefully explain where exactly any possible developmental differences are emerging before jumping into the subtraction directly.
3. The question of whether there are developmental trade-offs between statistical learning and executive functions is interesting. That said, there are a number of control analyses needed still. For instance, are there trade-offs in executive function and statistical learning within one Age Group (i.e. Do differences in Executive Function at Age 14 relate to differences in statistical learning at Age 14?)? Another example is that most of the variability in the learning trajectories appears to be coming from participants' starting point (i.e. how good their statistical learning was at Age 7), not the end point. This means that the authors' decision to use learning trajectories could mean that what they have found is that participants who are relatively better statistical learners at Age 7 go on to have relatively better executive functions at Age 14, which could then still be faciliatory or antagonistic at Age 14 (but is not accounted for in the current approach). I will note also that

these control analyses are related to my concerns about the learning score: for instance, if it is the case that better Working Memory scores at Age 14 are related to worse statistical learning scores, and a worse statistical learning score reflects someone who is at ceiling on reaction time performance, this would mean that those with better working memory have better reaction times (which is probably to be expected).
4. I would also urge the authors to be careful about the directionality with which they describe their longitudinal results. Right now, the analytic approach (using EF scores to model changes in the likelihood of a learning trajectory) and the way the authors describe the longitudinal relationship implies that differences in executive function are shaping changes in statistical learning performance. For instance, the authors write that "children with lower EF abilities at age 14 were more likely to improve their statistical learning abilities as they developed", which implies that a changes in executive function predict a change in statistical learning. But the longitudinal relationship these data could potentially speak to is the opposite: statistical learning changes might predict later executive functions.
5. As a smaller point, the authors' description of their task structure could be clearer in a number of places. For example, the authors say that a 'trial refers to a single element in the sequence' but then later in that paragraph say that 'of all trials, 62.5% ended with…". How can one element of a sequence end with something? (Should this perhaps read, "Of all triplets, 62.5% ended…"?). I wonder if the authors could just use "item" instead of trial? The Figure 1 caption also says that "every second element is part of an 8-element probabilistic sequence". My understanding from the authors' description of the task is that every element (trial) part of a probabilistic sequence, and that every second element is part of deterministic sequence. Please clarify. As a third example, it is also not clear to me why some of the highlighted ovals overlap in Figure 1b while others do not. It might also be helpful to explicitly state that there are no adjacent statistics that are useful for the participants to learn.
6. Finally, there are two different types of high-probability triplets. The majority are made up of a beginning and an ending that are both part "pattern" elements, while a small portion (12.5 of the total trials) are made up of "random" beginning and ending elements. Do participants behave similarly on these two types of trials? If not, it would be important to understand how they differ, and whether the authors' conclusions all hold when restricting their analyses to either type.

Reviewer #2

(Remarks to the Author)
I co-reviewed this manuscript with one of the reviewers who provided the listed reports. This is part of the Nature Communications initiative to facilitate training in peer review and to provide appropriate recognition for Early Career Researchers who co-review manuscripts.

Dear authors,

We read your manuscript Longitudinal evidence for decreasing statistical learning abilities across childhood with great interest. The topic is timely and of interest to readers of Nature Communications. In general, the manuscript is well-written and well-structured. The motivation for the study is clear, and its longitudinal design, the focus on statistical learning in isolation (rather than having it integrated with language of musicality) as well as the clear positioning of the study within research on models of cognitive development are strong points of this study. Despite, we have some issues and some questions for clarification that we hope the authors can address for a revised version of their manuscript.

Major points
1. Although the overall motivation for the study is clear, the author's choice for the ASRT task as a measure of statistical learning should be motivated. Why did the authors specifically use the ASRT task as a measure of statistical learning? They provide some clue in their method section when they mention a strength of the ASRT task (p.9, lines 268-271), but it would be helpful if they motivate their choice for this task in the Introduction as well. The ASRT task is also commonly used as a measure of Procedural learning, and it remains unclear whether the authors consider procedural learning and statistical learning as similar or different concepts. Furthermore, there has been considerable debate on the reliability of the serial reaction time task as an individual measure of statistical learning/procedural (e.g., West et al., 2017; 2018) this should be discussed somewhere in the manuscript.
2. Similarly, the authors choice of their EF-tasks requires a better motivation. For example, why not including an inhibition and switching task as well? And what is the rationale for including a verbal fluency task? For both the ASRT task (see point above) and the EF-tasks it would be nice if the reader received some explicit foreshadowing of what tasks were used to assess children, what they are supposed to measure and why they were suited for this longitudinal study/research question.
3. In both their Introduction and Discussion, the authors provide explanations for why developmental differences in statistical learning may occur. They discuss the role of prior knowledge. In this regard, it might be good to also cite work from Noam Siegelman's lab who explicitly investigated how prior knowledge (in their case prior linguistic knowledge) may impact statistical learning (not only developmentally, but also across modalities).
4. Relatedly, we do see how the outcomes of this study contribute to the "competition model" account. We do not necessarily see, however, how the outcomes of this study contribute to open-minded input driven vs. knowledge driven account as an explanation for developmental differences. The current set-up of the Study does not directly address/test for the role of prior/entrenched knowledge in the development of statistical learning. Therefore, the author's discussion of the findings in light of this second account (pages 16-17, lines 524-536) should address this, and explicitly mention that this part of their explanation is speculative.
5. Although we appreciate the visualizations of the design and outcomes, we found most of the figures not very clear and/or informative:
a. Figure 1B: It was not intuitive to us to visually understand the difference between high- and low-probability elements included in a triplet as shown in Figure 1B. Figure 1A and 1C serve, according to us, their purpose and provide a clear

visualization of the (part of) the task in combination with the Figure's description and in-text explanation, but Figure 1B remains unclear.
b. Figure 3: When figure 3A is mentioned in the text for the first time (p. 10; line 352) it was not yet clear to us that smaller differences between high and low triplets indicate decreased statistical learning (and this also doesn't become very clear from figure 3A). This meant that the interpretation of Figure 3A only became clear to us when the Age*TripletType interaction is explained later in the Results section (which also relates to Figures 3C and 3D).
c. Figure 4B: The figure is a bit hard to interpret, and we found the in-text explanation clearer.
d. Figures 4CD: we do not really see how these figures support the outcome that better EF ability at age 14 is related to a decreasing trajectory of statistical learning between ages 7 and 14, given that the individual scores/violin plots for the different subgroups overlap and thus suggest that there are no real differences between the different subgroups?
6. The authors may want to consider including the within-subject RTCV approach in the main paper instead of supplementary text. This approach addresses important issue in developmental psychology and they draw considerable conclusions from it in their Discussion.
7. One of the conclusions is that there is a competitive relationship between statistical learning and cognitive processes (i.e., children with higher EF ability were more likely to show a decreasing profile of statistical learning and vice versa). We wonder how the authors would explain these findings within theories on a statistical/procedural learning deficit in children with DLD, a group of children who often showed reduced EF abilities as well, which seems to contradict the competition model?
8. Relatedly, the Discussion may benefit from a more elaborate reflection on what it may mean (in practice/real life) or theoretically that there are differ subgroups of statistical learners? What is the practical and/theoretical merit of this finding?

Minor points
1. It would be helpful if the Introduction ends with an explicit outline of the research questions (as they are explicitly outlined in the Results section).
2. At several points in the manuscript, the authors refer to 'internal models'. Not every reader (including us) may be familiar with this term. As such it would be helpful if the authors could elaborate a bit more on what they mean with internal models.
3. Page 5, line 155. What is the purpose/functioning of providing this type of feedback to participants?
4. What is the purpose/function of Table 1? The overall mean accuracy scores and response times are not very informative for this type of task, are they? Also, it is not entirely clear what the missing data represents (individual trials?)
5. Page 9: the authors describe how their effects are coded in the linear mixed models, but if we are correct, they do not explain their coding of Age
6. On page 11, the authors introduce the concept learning scores (high-low triplet type differences). Are these differences in raw response times or are these differences based on the estimates from the model outcomes?
7. We found two typos:
o ASRT sequence line 179
o Backward digit span task acronym line 211

Reviewer #3

(Remarks to the Author)
I co-reviewed this manuscript with one of the reviewers who provided the listed reports. This is part of the Nature Communications initiative to facilitate training in peer review and to provide appropriate recognition for Early Career Researchers who co-review manuscripts.

Version 1:

Reviewer comments:

Reviewer #1

(Remarks to the Author)
The authors have addressed many of my concerns, but I am still not sure how to interpret their link between the executive function data and the statistical learning data. In particular, the authors' control analyses found that there is no relationship between the statistical learning score at 14 years of age, and the EF measures. This seems to contradict the argument that executive functions and statistical learning processes are at odds with one another across development. The authors state that because the statistical learning scores at age 7 do not relate to scores on the EF tasks, nor do the statistical learning scores at age 14, the interpretation should be that the developmental trajectory is what underpins this link. Can the authors elaborate on what they mean by this in a more mechanistic way? If the argument is that overall statistical learning performance is irrelevant to EF task performance (but only the direction of change in the preceding 7 years is predictive), I'm not sure this supports the conclusion that these tasks are tapping underlying cognitive functions which are increasingly at odds with one another across development.

Second, I also did not understand the authors' decision to restrict their 2nd control analyses for my concern about ceiling effects to the second half of the experiment (although I appreciate the many other control analyses which point towards the same conclusion!). My original concern was that a learning signal measured as a difference in reaction time might be, by definition, lower if the reaction times are at ceiling in a group of participants. By restricting this control analysis to periods of time which have the most restricted range already, the authors might have been least likely to capture variability due to my original concern. Could the authors please more carefully explain why it makes sense to restrict the analyses to the period of time when this difference is the smallest?

Reviewer #2

(Remarks to the Author)
Again, I co-reviewed this manuscript with one of the reviewers who provided the listed reports. This is part of the Nature Communications initiative to facilitate training in peer review and to provide appropriate recognition for Early Career Researchers who co-review manuscripts.

Dear authors, dear editor,

Overall the authors adequately addressed my comments (I was reviewer 2/3 in the previous round, and if they decided to not address my comment/suggestion in the revised manuscript they had good reasons to do so. Together with their changes in response to reviewer 1 I think the manscript definetely improved and I would accept the current version with only two minor comments/suggestions:

1. There are a few null results in the new analyses that aim to test whether an overall decrease in RTs can explain the age-related changes. Please be carefull in interpreting these null effects: in their discussion the authors now write that the observed decline in statistical learning cannot be explained by baseline RT confounds and instead reflects meaning age-related changes. This conclusion is formulated a bit too strong: one can only conclude from the null effect that there is no evidence that the observed decline in statistical learning can be explained by baseline RT confounds, which means something slightly different. Relatedly, the authors write in their Discussion that EF measures did not correlate with statistical learning at either age 7 or 14 and that this suggests that the observed relationship is specific to the developmental trajectory itself. A better formulation would be that they found no evidence that EF measures correlate with statistical learning at either age 7 or 14, which may suggest that […].

2. The authors did a good job in clarifying their Figures. However, I still had some difficulty understanding the difference between the low probability and high probability triplets (and Figure 1 did not immediately help). For me, it would help if the authors make explicit that in low propability trials (e.g., 2-X-1 and 2-X-2) the start and end trials are random and the X-element is part of the pattern (2, 4, 3, 1 in the concrete example from Figure 1), whereas in the high probability trials the start and end trial are part of the pattern and the X-element is random.

Reviewer #3

(Remarks to the Author)
I co-reviewed this manuscript with one of the reviewers who provided the listed reports. This is part of the Nature Communications initiative to facilitate training in peer review and to provide appropriate recognition for Early Career Researchers who co-review manuscripts.

Version 2:

Reviewer comments:

Reviewer #1

(Remarks to the Author)
Dear editors and authors,
I re-read the manuscript and read the authors' response to my concerns about claiming a trade-off or support for a competitive model between statistical learning and executive functions across development. While I agree that the trajectory analyses are interesting and worth reporting, I still find the that claim that this means statistical learning and executive functions are competitive processes, or at odds with one another, across development is not well supported.
A primary concern is that the manuscript and rebuttal present a somewhat misleading account of the neurocognitive mechanisms underlying statistical learning. The text implies that statistical learning is uniquely supported by striatal processes, whereas hippocampal and prefrontal regions are not involved. For example, the authors state, "During early and middle childhood, striatal systems that support input-driven learning, including SL, are dominant and function efficiently even in infants and young children3,11,81." However, these citations do not demonstrate that striatal systems support statistical learning, nor uniquely: one paper shows that infants can perform well on statistical learning tasks, another reports developmental changes in striatal connectivity during adolescence, and a third shows reaction time changes across childhood in statistical learning tasks—but none of these report any evidence linking striatal function to statistical learning. While there is evidence that the basal ganglia are involved in statistical learning in adults and children (e.g. Mcnealy et al., 2010; Turk-Browne et al., 2009), there is much evidence that the hippocampus and prefrontal cortex also contribute to statistical learning in both adults (see, for instance: Schapiro et al., 2016; Schapiro et al., 2017; Turk-Browne et al., 2009; Covington et al., 2018; Schapiro et al., 2014) and young learners (Ellis et al., 2021; Finn et al., 2019; Schlichting et al. 2017). Thus, building a theoretical argument based on striatal and hippocampal/prefrontal development to describe potential trade-offs between statistical learning and executive functions in the developing brain (especially without neural evidence) is premature. It is, of course, possible that these systems contribute differently across development and that the developmental trade-offs you suggest do exist, but the current evidence does not firmly support this claim. I leave the final decision to the editor, but I recommend that the authors either remove these claims, or frame their mechanistic claims much more cautiously throughout the manuscript (for instance, the final paragraph still includes this claim), with more careful citations,

acknowledging both the broader neural contributions and the speculative nature of developmental interactions.

Reviewer #2

(Remarks to the Author)
Dear authors,

Thank you for your resubmission. You've addressed the comments of my previous revision and I look forward seeing your manuscript published.

Reviewer #3

(Remarks to the Author)
I co-reviewed this manuscript with one of the reviewers who provided the listed reports. This is part of the Nature Communications initiative to facilitate training in peer review and to provide appropriate recognition for Early Career Researchers who co-review manuscripts.

Reviewer #4

(Remarks to the Author)
I was asked to review the LCA methodology.

Whilst it is commendable that authors applies this method, the fit statistics strongly indicated that the two-class solution provided the most appropriate balance of model fit, parsimony, and classification quality. Both AIC and SABIC favoured the two-class model (lower values indicating better fit). Entropy for the three-class solution also fell to 0.65 from 0.95, indicating poor class separation and uncertainty in individual class assignments. Entropy < 0.70 is generally considered unacceptable for LCA as it indicates that the classes overlap too much. Class sizes further supported this: both the two- and three-class models generated a very small class containing only 3 individuals, suggesting this subgroup reflected statistical noise/outliers rather than a substantively meaningful latent class. In contrast, the remaining classes in the three-class model were split into two larger groups without any corresponding improvement in fit, implying over-extraction or an artefact of the method used. The authors do not specify if they considered specifying time as non-linear (e.g, using polynomial or spline functions). Furthermore, a different clustering algorithm could yield different results. The authors have not provided sufficient justification for retaining the third class. In Figure 4, the CIs cross, indicating no difference in scores.

For the "Increase" class, the authors mention that there is a "slight increase in average learning scores with development". Is this an appropriate label? How much of an increase would be considered meaningful (e.g., could this be judged on standard deviation increase between the first and last time-points?). What about those who have a stable trajectory? I believe more exploratory work should be undertaken to understand if these classes are real.

Furthermore, because one of the latent classes contained only three individuals, I don't believe it is methodologically appropriate to estimate predictors of class membership using multinomial regression. Multinomial models require sufficient observations within each outcome category to obtain stable parameter estimates, and extremely small classes produce unreliable or non-estimable coefficients, and large standard errors. In this case, the tiny class size, combined with the poor entropy observed in the three-class solution, indicates that the class is statistically unstable and unlikely to represent a meaningful underlying subgroup. Running a multinomial regression would, therefore, yield results that are neither robust nor interpretable. It was difficult to judge the results of the multinomial regression as no regression table was provided and it wasn't clear what the reference category was.

Version 3:

Reviewer comments:

Reviewer #1

(Remarks to the Author)
I have no further comments. Thanks!

Reviewer #4

(Remarks to the Author)
That authors have addressed my comments. They conducted substantial sensitivity analyses, which gives me greater confidence in the results.

Open Access This Peer Review File is licensed under a Creative Commons Attribution 4.0 International License, which permits use, sharing, adaptation, distribution and reproduction in any medium or format, as long as you give appropriate credit to the original author(s) and the source, provide a link to the Creative Commons license, and indicate if changes were made.
In cases where reviewers are anonymous, credit should be given to 'Anonymous Referee' and the source.
The images or other third party material in this Peer Review File are included in the article's Creative Commons license, unless indicated otherwise in a credit line to the material. If material is not included in the article's Creative Commons license and your intended use is not permitted by statutory regulation or exceeds the permitted use, you will need to obtain permission directly from the copyright holder.
To view a copy of this license, visit https://creativecommons.org/licenses/by/4.0/

Addressing the issues highlighted in the Reviewers' comments

Reviewer #1

Comment #1

The authors of 'Longitudinal evidence for decreasing statistical learning abilities across childhood' present data from an ASRT task at four longitudinal timepoints, and a battery of executive function (EF) tasks at the 4th time-point. They then measure the difference in response times to the targets which are easier versus harder to predict based on the statistical structure of a spatial sequence. The main findings are that participants 'statistical learning score' (operationalized as the difference in reaction time to hard to predict targets and easy to predict targets) goes down across developmental time, and that participants who show better EF at time-point 4 are more likely to have shown this developmental trajectory. I commend the authors extensive longitudinal data collection and attempt to link developmental trajectories in statistical learning to other cognitive processes—these questions are timely and of interest to developmental and cognitive psychologists. I have some reservations about the authors primary claims, based off the way they operationalized their statistical learning score. I detail these and other questions below.

Response #1

We are grateful to the Reviewer for devoting their attention to our manuscript and for their constructive comments. In response to these comments, we have carefully revised the manuscript and conducted several control analyses to demonstrate that the age-related decline in learning scores is both meaningful and robust. We believe that these revisions have strengthened the manuscript significantly.

Comment #2

My primary concern is about how the authors chose to operationalize a 'statistical learning score' as the difference between reaction times to high probability trials (i.e. targets at the end of a high-probability triplet) and low probability trials (i.e. targets at the end of low-probability triplets). The youngest children show the biggest difference in these trials, while the oldest participants show the smallest difference. My concern is that the older participants are essentially at ceiling in their reaction times. This is supported by the finding that there is less of a decrease in reaction times across blocks in the older participants (which the authors explain as "likely stemm[ing] from faster initial reaction times, leaving less room to improve at later ages."), and leads to the possibility that the small difference in RTs to high and low probability targets is because the older participants are already responding very quickly to both types of trials. This would mean the authors have minimized their chances to observe any difference in the reaction time to high and low probability targets in the older groups (and might help explain why the accuracy results presented in the supplement show a different pattern). Figure 1a also makes it look like the error bars between the low and high probability targets are less overlapping in older participants than younger participants, suggesting that the difference in responses to these trial types might actually be more distinct in older groups.

Response #2

We thank the Reviewer for raising the question of age-related changes in baseline reaction times (RTs). We agree that this is an important aspect to consider when comparing data across

different ages. We sought to further examine the potential impact of age-related changes in RTs on statistical learning by three additional sets of analyses.

Before we present these results, we would like to note that in order to more clearly present the sources of interindividual variability, we adjusted our initial random effects structure so that the model now contains uncorrelated by-participant intercepts and slopes for Block, Age, and their interaction as random effects. In terms of *lmer* syntax, we changed from "(Block | Participant:Age)" to "(Block*Age || Participant)". The sources of interindividual variability are now clearly explained in the Results section (pp. 11). Briefly, the results of the random effects confirm, as expected, that most interindividual variability comes from differences in overall reaction time levels at different ages. Importantly, all results initially reported were unchanged in this model with the updated random effects structure.

We now turn to the three sets of analyses conducted to examine the potential impact of age-related changes in RTs on statistical learning.

First, if lower RTs are generally associated with lower learning scores (leading to a ceiling effect), this should be evident not only across ages but also *between individuals* within a certain age group. To test this, we performed correlational analyses, and found no significant association between overall RTs and statistical learning scores at any age (age 7: $r = 0.17$, $p = .127$; age 8: $r = 0.11$, $p = .406$; age 11: $r = 0.10$, $p = .406$; age 14: $r = 0.16$, $p = .116$, see also Response #4). A similar absence of correlation between overall RTs and statistical learning scores was reported across nine distinct age groups–ranging from 7-8 to 61-85 years–in a cross-sectional ASRT study spanning the human lifespan[1]. Our result, as well as the findings of Juhasz et al.[1], demonstrate that lower RTs are not associated with lower learning scores. This indicates that the age-related decline in learning scores observed in our main analysis was not due to a parallel decrease in RTs.

Second, following the same logic, if lower RTs are systematically associated with lower statistical learning scores (and lead to a ceiling effect), then this relationship should also be evident *within individuals* in a single ASRT session. In other words, for a given individual, the blocks where they respond faster should also show lower learning scores. We sought to examine this by calculating the repeated measures correlation[2] of median RTs and statistical learning scores across blocks for each ASRT session. This approach estimates the common within-individual association between two continuous variables, accounting for non-independence due to repeated measurements. Unlike simple correlation, repeated measures correlation removes between-subject variance, isolating the consistent relationship across individuals. It fits parallel regression lines (same slope, different intercepts) for each individual and computes a shared slope-based correlation coefficient. This method provides a more accurate and powerful estimate of within-subject associations than traditional correlation or aggregation approaches. We restricted this analysis to the second half of the task (blocks 11 to 20) to make sure that some learning has already occurred. This analysis revealed a statistically significant, weak positive relationship at age 7, $r_{rm}(755) = 0.100$, 95% CI = [0.029, 0.170], $p = 0.006$, but no relationship at any other age, Age 8: $r_{rm}(547) = 0.006$, 95% CI = [-0.078, 0.090], $p = 0.890$; Age 11: $r_{rm}(600) = 0.014$, 95% CI = [-0.066, 0.093], $p = 0.738$; Age 14: $r_{rm}(895) = 0.030$, 95% CI = [-0.037, 0.097], $p = 0.377$. Although a statistically significant correlation was found at age 7, the association between overall RTs and learning scores was weak, with a very small correlation coefficient. Importantly, this association was completely absent at all other ages, including age 14, a critical point in our dataset where potential confounding could arise due to near-ceiling performance, reflected in the lowest overall RTs in the dataset. These findings

provide further evidence that differences in overall RTs are unlikely to have significantly confounded our main results.

Third, we conducted an additional analysis based on an approach proposed in the literature to account for potential confounding effects of overall RTs on learning measures[1]. This approach involves selecting subsets of participants with comparable overall RTs within each age group and testing learning effects within these subgroups. Using this method, Juhasz et al.[1] showed that the greater within-session improvement in younger age groups could not be explained by generally slower RTs. This approach had to be adjusted for our longitudinal design as selectively including only certain assessment timepoints for a participant—while discarding others—would have undermined the integrity of the dataset. Therefore, we excluded participants who at age 14 fell within the lowest fifth of the sample in terms of their overall RTs and thus were the most likely to have reached ceiling performance, and fit our LMM on this dataset (N = 76). The Age × Triplet type interaction remained significant ($F_{(3,9121.34)} = 3.72$, $p = .011$), and the age-related decrease in statistical learning was still clearly present (learning score contrasts at Age 7: 24.00 ms, 95% CI = [17.61, 30.50]; Age 8: 20.00 ms, 95% CI = [12.40, 27.60]; Age 11: 13.80 ms, 95% CI = [6.56, 21.00]; Age 14: 10.00 ms, 95% CI = [4.02, 16.00]). We replicated this analysis while excluding the fastest fourth and fastest third of participants, respectively. The results remained remarkably consistent across all models (see Working Figure 1). In addition, to demonstrate that any changes in these models (wider confidence intervals and variability in p-values) reflect reduced statistical power due to the smaller sample size rather than selective exclusion of participants with the lowest overall RTs at age 14, we conducted further sensitivity analyses. We repeated the subsampling procedure, this time excluding the same number of participants at random instead of based on their RTs. These sensitivity analyses yielded highly comparable results, indicating that reduced sample size–not RT-based selection–was responsible for the wider confidence intervals and variability of p-values for the Age × Triplet Type interaction. Across all analyses, the age-related decline in statistical learning remained a consistent and robust finding.

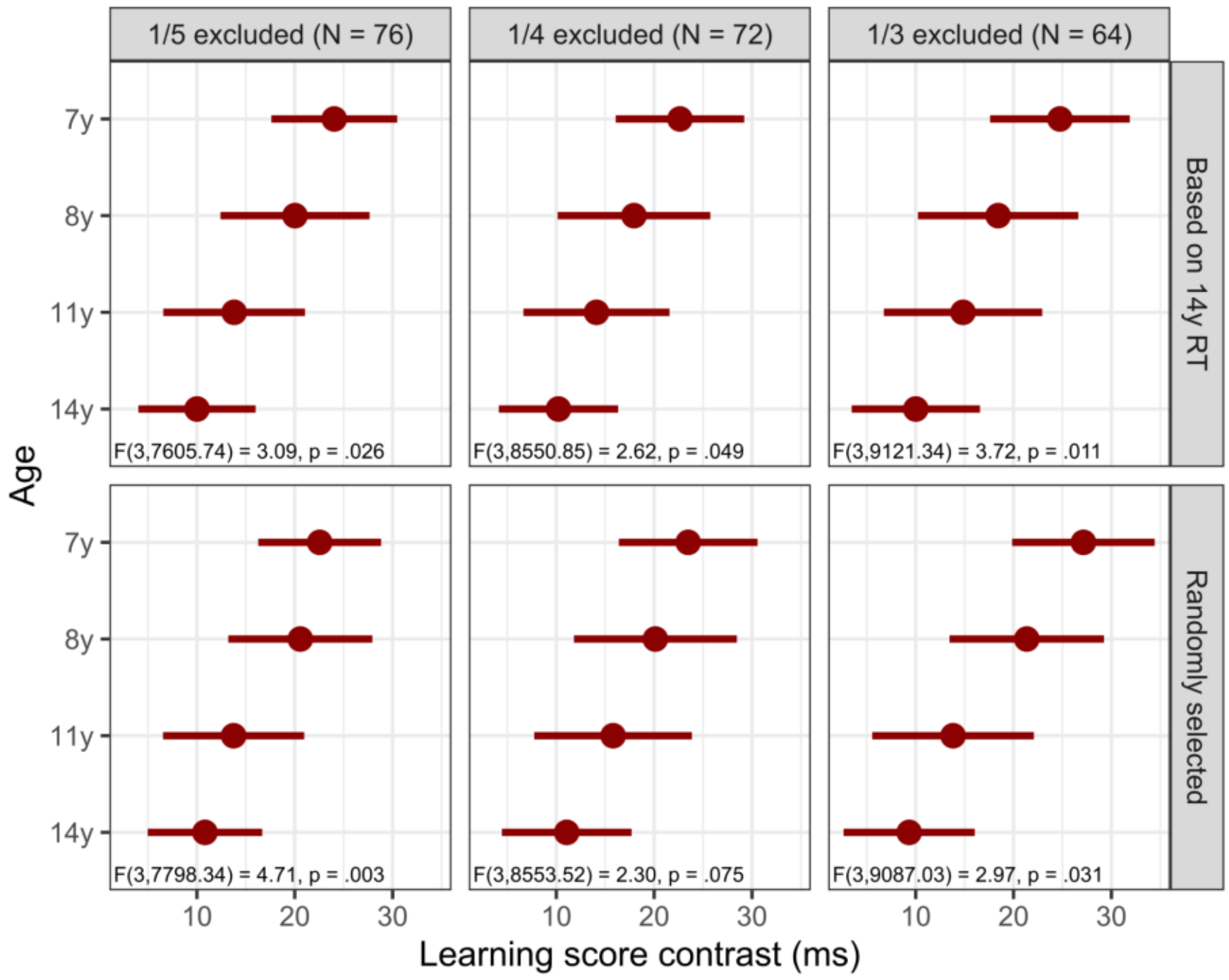

Working Figure 1. Sensitivity analyses with listwise exclusion of participants based on either their RTs at age 14 (top), or at random (bottom). Shown are the learning score contrasts and their 95% CI on the X-axis as a function of age on the Y-axis, separately for excluding one third, one fourth, or one fifth of participants. The results of the corresponding F test of the Age × Triplet interaction are also shown on the bottom left of each panel. The age-related decrease in statistical learning scores is present in all models.

In summary, our comprehensive set of control analyses–including between- and within-subject correlations, subset analyses, and sensitivity tests–consistently indicate that the observed age-related decline in statistical learning is not due to the parallel decrease in overall RTs across development. We have incorporated all control analyses in the revised manuscript (pp. 16-18).

Comment #3

Relatedly, could this smaller subtraction score also imply that older participants are doing relatively better on the less predictable trials than younger participants? I understand from the authors' citations that previous reports of statistical learning using this task have used the difference between response times to high and low probability targets, but much of the ongoing work studying developmental differences in statistical learning is acknowledging that what learners of different ages might be learning about their statistical experiences is different. One possibility is that the younger ages are pretty good at learning about the strong predictive relationships, but struggle with the less predictive ones, while older children are able to also learn the less common relationships in the low-probability triplets—this could also lead to a smaller difference in the reaction times across these trial types with age. It will be important to more carefully explain where exactly any possible developmental differences are emerging before jumping into the subtraction directly.

Response #3

We thank the Reviewer for raising this important point. To address the concern, we conducted additional analyses examining developmental changes in RTs for low- and high-probability triplets separately.

We found that RTs for both triplet types followed highly similar developmental trajectories. Specifically, for low-probability triplets, the estimated RT differences between consecutive ages were as follows: from age 7 to 8: -192 ms, 95% CI = [-228, -156]; from 8 to 11: -94 ms, 95% CI = [-120, -67]; and from 11 to 14: -103 ms, 95% CI = [-143, -63] (all ps < .001). For high-probability triplets, the corresponding RT differences were: 7 to 8: -188 ms, 95% CI = [-224, -151]; 8 to 11: -88 ms, 95% CI = [-114, -62]; and 11 to 14: -100 ms, 95% CI = [-140, -60] (all *ps* < .001).

Next, we directly compared these developmental trajectories to determine whether participants began responding differently to either the low- or high-probability triplets at any specific time point. That is, we examined whether the magnitude of RT change from ages 7 to 8, 8 to 11, and 11 to 14 differed significantly between the two triplet types. The contrasts revealed no significant differences across any of the intervals (from age 7 to 8: -3.90 ms, p = .381; from 8 to 11: -5.69 ms, p = .225; from 11 to 14: -3.60 ms, p = .394).

These results indicate that the significant Age × Triplet interaction observed in our main model reflects a decrease in the RT *difference* between high- and low-probability triplets over

development, rather than diverging developmental patterns for the two triplet types themselves. This analysis is now included in the revised manuscript (pp. 12).

Taken together with the control analyses presented in Responses #2 and #4, these results support the interpretation that the learning score used in our study captures a meaningful developmental change in statistical learning itself.

Comment #4

The question of whether there are developmental trade-offs between statistical learning and executive functions is interesting. That said, there are a number of control analyses needed still. For instance, are there trade-offs in executive function and statistical learning within one Age Group (i.e. Do differences in Executive Function at Age 14 relate to differences in statistical learning at Age 14?)? Another example is that most of the variability in the learning trajectories appears to be coming from participants' starting point (i.e. how good their statistical learning was at Age 7), not the end point. This means that the authors' decision to use learning trajectories could mean that what they have found is that participants who are relatively better statistical learners at Age 7 go on to have relatively better executive functions at Age 14, which could then still be facilitatory or antagonistic at Age 14 (but is not accounted for in the current approach). I will note also that these control analyses are related to my concerns about the learning score: for instance, if it is the case that better Working Memory scores at Age 14 are related to worse statistical learning scores, and a worse statistical learning score reflects someone who is at ceiling on reaction time performance, this would mean that those with better working memory have better reaction times (which is probably to be expected).

Response #4

We appreciate the Reviewer's suggestions regarding additional control analyses, and we conducted the suggested analyses accordingly.

First, we examined whether statistical learning performance was related to executive function scores at age 14. Correlational analyses revealed no significant association between statistical learning and either the Working Memory (WM) factor (r = -0.06, p = .546) or the Fluency factor (r = 0.11, p = .271).

Next, in contrast to the Reviewer's suggestion that our results may reflect that "participants who are relatively better statistical learners at Age 7 go on to have relatively better executive functions at Age 14", we found no strong evidence for such a relationship. Specifically, statistical learning scores at age 7 showed no significant correlation with WM or Fluency factor scores at age 14 (r = 0.09, p = .402 and r = 0.14, p = .210, respectively). These findings suggest that the observed negative relationship between the *decrease* in statistical learning performance from ages 7 to 14 and executive functions at age 14 indeed relates to the longitudinal trajectory and not to a correlation between the two cognitive processes at either time point.

Finally, we also tested whether overall RTs were associated with executive function and statistical learning scores. As expected, faster RTs at age 14 were significantly, albeit weakly associated with better executive functioning, as evidenced by negative correlations with both the WM factor (r = -0.33, p < .001) and the Fluency factor (p = -0.24, p = .018). However, RTs were not significantly correlated with statistical learning scores at age 14 (r = 0.16, p = .116), nor at any other assessment point (age 7: r = 0.17, p = .127; age 8: r = 0.11, p = .406; age 11: r = 0.10, p = .406). These findings are consistent with results from a cross-sectional ASRT study

by Juhasz et al.[1], which also reported no correlation between overall RTs and statistical learning scores.

Taken together, these control analyses indicated that while individuals with better executive functions at age 14 tend to respond generally faster in the ASRT task, their statistical learning scores appear to be independent of both baseline speed and executive function performance at that age. These control analyses are now included in the revised manuscript (pp. 15).

Comment #5

I would also urge the authors to be careful about the directionality with which they describe their longitudinal results. Right now, the analytic approach (using EF scores to model changes in the likelihood of a learning trajectory) and the way the authors describe the longitudinal relationship implies that differences in executive function are shaping changes in statistical learning performance. For instance, the authors write that "children with lower EF abilities at age 14 were more likely to improve their statistical learning abilities as they developed", which implies that a changes in executive function predict a change in statistical learning. But the longitudinal relationship these data could potentially speak to is the opposite: statistical learning changes might predict later executive functions.

Response #5

We thank the Reviewer for raising this important point. Our analytical approach is grounded in the theoretical frameworks that are most relevant to our study.

One central model guiding our interpretation is the competition model, which posits that as top-down, internally-driven processes–such as executive functions–mature, individuals become progressively less sensitive to raw environmental regularities, which are typically extracted through bottom-up, input-driven mechanisms. This reduced sensitivity, in turn, leads to decreased statistical learning performance[1,3,4].

Several complementary theoretical accounts suggest a similar developmental dynamic between bottom-up and top-down cognitive processes. For instance, Wu et al.[5] propose that in childhood, learning tends to be input-driven and open-minded due to limited prior experience. This form of learning is well-suited for detecting raw environmental regularities, a core component of statistical learning. As individuals mature and accumulate knowledge, they tend to rely more heavily on knowledge-driven learning strategies, which can be related to goal-directed behaviour and executive functions. This developmental shift may introduce cognitive biases that reduce sensitivity to new environmental patterns, potentially explaining the observed decline in statistical learning during adolescence and adulthood.

A related perspective comes from the developmental account of the explore/exploit trade-off[6], which describes how the balance between exploring new information and exploiting existing knowledge changes over time. In early development, children tend to favor exploration over exploitation, facilitating broader learning from environmental input. With increasing age and accumulated experience, individuals shift toward exploitation, relying more heavily on prior knowledge to guide behavior. This shift again implies greater engagement of top-down processes, which may constrain the bottom-up, input-driven detection of novel environmental regularities.

Drawing on these theoretical frameworks, and in alignment with our study's primary objective to investigate the developmental trajectory of statistical learning, we focused on how

emerging top-down processes may influence this capacity. We believe this direction of influence is the most appropriate and theoretically supported. Nevertheless, we acknowledge that our study design does not allow for causal inferences. Therefore, in the revised manuscript, we have moderated the assertions that suggest directionality and added a new paragraph discussing this limitation explicitly (pp. 19-20).

Comment #6

As a smaller point, the authors' description of their task structure could be clearer in a number of places. For example, the authors say that a 'trial refers to a single element in the sequence' but then later in that paragraph say that 'of all trials, 62.5% ended with…". How can one element of a sequence end with something? (Should this perhaps read, "Of all triplets, 62.5% ended…"?). I wonder if the authors could just use "item" instead of trial? The Figure 1 caption also says that "every second element is part of an 8-element probabilistic sequence". My understanding from the authors' description of the task is that every element (trial) part of a probabilistic sequence, and that every second element is part of deterministic sequence. Please clarify. As a third example, it is also not clear to me why some of the highlighted ovals overlap in Figure 1b while others do not. It might also be helpful to explicitly state that there are no adjacent statistics that are useful for the participants to learn.

Response #6

We thank the Reviewer for drawing our attention to the vague parts of the task description. We thoroughly revised both the main text and Figure 1 caption and added more information to make the task description clearer. Please see the revised manuscript for the relevant changes (pp. 5-7).

Comment #7

Finally, there are two different types of high-probability triplets. The majority are made up of a beginning and an ending that are both part "pattern" elements, while a small portion (12.5 of the total trials) are made up of "random" beginning and ending elements. Do participants behave similarly on these two types of trials? If not, it would be important to understand how they differ, and whether the authors' conclusions all hold when restricting their analyses to either type.

Response #7

We thank the Reviewer for raising this question. We interpreted this comment as a suggestion to investigate the potential differences in responses to Random high and Pattern high triplets, and whether such differences might have affected the Age × Triplet type interaction in our main results. To address this, we re-ran our primary model, this time including only Random high and Pattern high triplet types. Specifically, we predicted block-wise median RTs with a linear mixed model containing Triplet type (Random high vs. Pattern high), Block (Block 1-20), and Age (7, 8, 11 or 14), along with all their higher order interactions as fixed effects, and uncorrelated by-participant intercepts and slopes for Block, Age, and their interaction as random effects.

The model revealed a significant main effect of Block ($F_{(1, 85.21)} = 259.36$, $p < .001$), indicating that RTs decreased over the course of the task ($b = -7.93$, 95% CI = [-8.91, -6.95]). The main effect of Age was also significant ($F_{(3, 96.80)} = 143.85$, $p < .001$), reflecting faster

overall RTss with increasing age (Age 7: 779 ms, 95% CI = [752, 807]; Age 8: 589 ms, 95% CI = [567, 612]; Age 11: 502 ms, 95% CI = [483, 522]; Age 14: 401 ms, 95% CI = [370, 431]; with all pairwise comparisons significant at $p < .001$). The Age × Block interaction was also significant ($F_{(3,\ 98.88)} = 17.45$, $p < .001$), with post hoc estimated marginal trends revealing a progressively less steep slope for Block with development (Age 7: b = -12.07, 95% CI = [-13.7, -10.18]; Age 8: b = -10.20, 95% CI = [-11.69, -8.71]; Age 11: b = -6.38, 95% CI = [-7.72, -5.05]; Age 14: b = -3.06, 95% CI = [-5.05, -1.06]; all pairwise comparisons significant at $ps < .016$, except between age 7 and 8).

While there was a significant Triplet type main effect ($F_{(1,\ 11587.34)} = 7.28$, $p = .007$), with slightly faster RTs for Random high triplets (Random high: 565 ms, 95% CI = [549, 581]; Pattern high: 570 ms, 95% CI = [555, 586]), this effect was much smaller than the difference found between low- and high-probability triplets in the main analysis (which as a reminder was: high-probability: 568 ms, 95% CI = [552, 583]; low-probability: 585 ms, 95% CI = [569, 600]). This pattern has been observed in previous ASRT studies as well[7–10] and is thought to reflect structural features of the ASRT task–specifically, the same triplet tends to reappear after shorter intervals in random trials compared to pattern trials[9].

Crucially, none of the interactions involving Triplet type were significant, Triplet type × Block: $F_{(1,\ 11620.82)} = 1.23$, $p = .267$, Triplet type × Age: $F_{(3,\ 11585.72)} = 0.30$, $p = .823$, and Triplet type × Age × Block: $F_{(3,\ 11614.91)} = 0.48$, $p = .695$. This indicates that the subtle difference between Random high and Pattern high triplets did not vary meaningfully across development or task progression (see Working Figure 4). Consequently, these results suggest that the distinction between Pattern high and Random high triplets is unlikely to have significantly influenced or confounded the Age × Triplet type interaction reported in our main analysis.

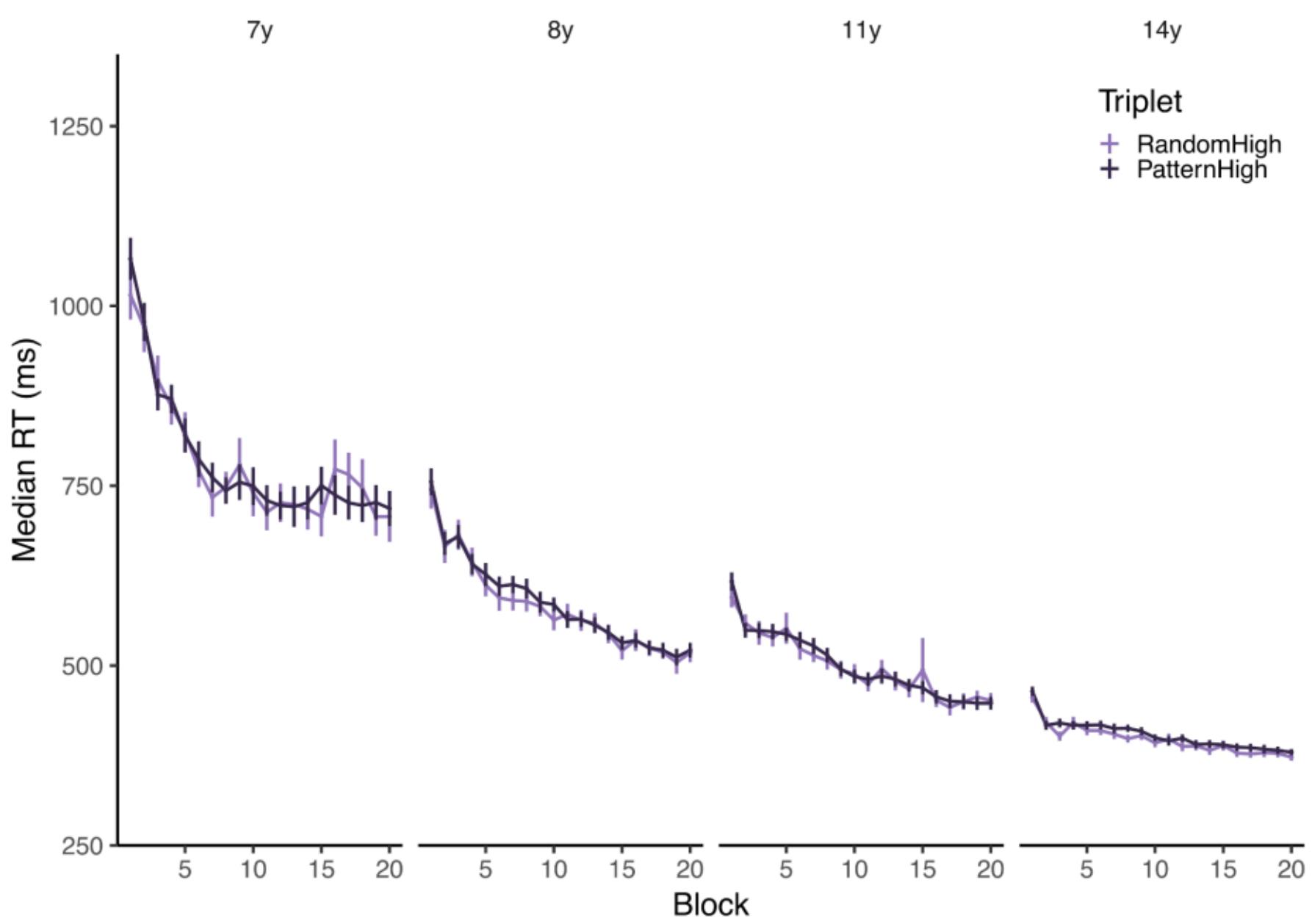

Working Figure 4. Average block-wise median reaction times as a function of triplet type (light purple for Random high, dark indigo for Pattern high) for the four ages tested.

<u>Reviewer #2 & #3</u>

I co-reviewed this manuscript with one of the reviewers who provided the listed reports. This is part of the Nature Communications initiative to facilitate training in peer review and to provide appropriate recognition for Early Career Researchers who co-review manuscripts.

Comment #1

Dear authors,

We read your manuscript Longitudinal evidence for decreasing statistical learning abilities across childhood with great interest. The topic is timely and of interest to readers of Nature Communications. In general, the manuscript is well-written and well-structured. The motivation for the study is clear, and its longitudinal design, the focus on statistical learning in isolation (rather than having it integrated with language of musicality) as well as the clear positioning of the study within research on models of cognitive development are strong points of this study. Despite, we have some issues and some questions for clarification that we hope the authors can address for a revised version of their manuscript.

Response #1

We thank the Reviewers for their thorough evaluation of the manuscript. We have taken the comments into account and revised the manuscript accordingly. Specifically, we have included our reasoning behind choosing the tasks employed in our study and revised the figures and figure legends to enhance their understandability. We believe that these changes have improved the manuscript significantly.

Major points

Comment #2

Although the overall motivation for the study is clear, the author's choice for the ASRT task as a measure of statistical learning should be motivated. Why did the authors specifically use the ASRT task as a measure of statistical learning? They provide some clue in their method section when they mention a strength of the ASRT task (p.9, lines 268-271), but it would be helpful if they motivate their choice for this task in the Introduction as well. The ASRT task is also commonly used as a measure of Procedural learning, and it remains unclear whether the authors consider procedural learning and statistical learning as similar or different concepts. Furthermore, there has been considerable debate on the reliability of the serial reaction time task as an individual measure of statistical learning/procedural (e.g., West et al., 2017; 2018) this should be discussed somewhere in the manuscript.

Response #2

We thank the Reviewers for highlighting this important issue. We selected the ASRT task as our measure of statistical learning for several reasons.

First, previous large-scale cross-sectional studies[1,11] also employed the ASRT task. Using the same task enables direct comparison with these studies, allowing our findings to be contextualized within the broader literature.

Second, we aimed to use a task that provides online measures of learning, rather than offline ones. Tasks involving offline measures such as two-alternative forced choice paradigms mostly rely on judgments that participants are required to make following the learning phase. These tasks primarily assess the outcomes of the learning process, rather than the learning process itself, and are potentially confounded by individual differences in memory capacity and metacognitive abilities. In contrast, online measures like those provided by the ASRT task allow for continuous assessment of learning as it unfolds, offering insights into the process of learning itself[12,13]. These online learning measures could be considered more sensitive and reliable indicators of learning.

Third, we chose the ASRT task for its relative reliability. Although we are aware of the ongoing debate on the reliability of certain serial reaction time (SRT) tasks, it is important to note that West and colleagues criticize the deterministic SRT task, which indeed shows low reliability. The ASRT task, in contrast, has demonstrated higher reliability[14] and robust learning effects across a range of designs and samples[15–17]. We now clarify these motivations in the revised Introduction (pp. 4).

We also thank the Reviewers for raising the question regarding the relationship between procedural and statistical learning. There are several assumptions in the literature about this relationship (and also, about the relationship between implicit learning and sequence learning). As Bogaerts, Siegelman and Frost[18] point out in their paper, the conceptual boundaries between these constructs are often blurred. We agree with their assessment and consider these constructs to be closely related, though not interchangeable. In their proposed framework[18], implicit learning is presented as an overarching category, encompassing procedural learning, (visual) statistical learning and sequence learning, with notable overlaps between these processes.

We believe that while procedural and statistical learning share commonalities as they both involve the acquisition of statistical regularities, they reflect distinct constructs. For example, statistical learning can occur without proceduralization (i.e., in an embedded pattern task with forced-choice testing). At the task level, the ASRT task likely taps into multiple learning processes: statistical learning is likely to dominate early in the task as participants extract regularities, followed by proceduralization of the acquired knowledge as practice continues.

In our manuscript, we chose to refer exclusively to statistical learning to maintain conceptual clarity and avoid overwhelming readers with overlapping terminology. However, we included procedural memory as a keyword to ensure visibility among researchers in the procedural learning domain. We have now added a sentence to the manuscript briefly linking statistical learning to procedural memory but refrained from an in-depth discussion of their relationship, as this would go beyond the scope of the current study.

Comment #3

Similarly, the authors' choice of their EF-tasks requires a better motivation. For example, why not including an inhibition and switching task as well? And what is the rationale for including a verbal fluency task? For both the ASRT task (see point above) and the EF-tasks it would be nice if the reader received some explicit foreshadowing of what tasks were used to assess children, what they are supposed to measure and why they were suited for this longitudinal study/research question.

Response #3

We appreciate the Reviewer's insightful comment. First and foremost, we would like to emphasize that all tasks included in this study are well-established, reliable, and widely used across the lifespan[19–23].

Ideally, a comprehensive assessment of executive functions would involve multiple tasks for each factor, administered alongside the statistical learning task. However, several practical constraints shaped our study design. Given that we conducted assessments in a school setting with children as young as seven years old, it was essential to limit each session to approximately 45 minutes—the standard duration of a classroom lesson—in order to maintain engagement and minimize fatigue. The ASRT task alone required 25–30 minutes to complete, leaving us with only 15–20 minutes to assess executive functions.

In light of these constraints, we prioritized a detailed assessment of the working memory (updating) factor, covering verbal, visuo-spatial, and complex working memory, alongside the verbal fluency task, which taps into multiple executive components, including shifting, inhibition, and updating[24]. This selection was informed by prior findings demonstrating an antagonistic relationship between statistical learning and executive functions, particularly working memory and verbal fluency, in both neurotypical young adults[4] and clinical populations[25]. Notably, our test selection has been further supported by a recent study[26], which reported negative correlations between statistical learning and executive function factors that included verbal fluency, counting span, and digit span across two experiments with neurotypical adults .

Nevertheless, in future studies, it would be beneficial to explore the relationship between statistical learning and other executive function factors, such as cognitive flexibility and inhibition, particularly during childhood. In the revised manuscript, we have now included additional information regarding our task selection rationale in the Introduction (pp. 4).

Comment #4

In both their Introduction and Discussion, the authors provide explanations for why developmental differences in statistical learning may occur. They discuss the role of prior knowledge. In this regard, it might be good to also cite work from Noam Siegelman's lab who explicitly investigated how prior knowledge (in their case prior linguistic knowledge) may impact statistical learning (not only developmentally, but also across modalities).

Response #4

We thank the Reviewers for drawing our attention to these findings, we have now incorporated them into the revised Discussion (pp. 18).

Comment #5

Relatedly, we do see how the outcomes of this study contribute to the "competition model" account. We do not necessarily see, however, how the outcomes of this study contribute to open-minded input driven vs. knowledge driven account as an explanation for developmental differences. The current set-up of the Study does not directly address/test for the role of prior/entrenched knowledge in the development of statistical learning. Therefore, the author's discussion of the findings in light of this second account (pages 16-17, lines 524-536) should address this, and explicitly mention that this part of their explanation is speculative.

Response #5

We thank the Reviewers for bringing this issue to our attention. Our intention referencing these frameworks was to illustrate that the interplay between top-down and bottom-up cognitive processes is a recurring theme across multiple areas of cognitive science. However, we acknowledge that our original manuscript did not clearly communicate this rationale. Therefore, we revised both the Introduction and Discussion to clarify that our study did not assess participants' prior knowledge. Please, see the modifications in the revised manuscript (pp. 4 and 19).

Comment #6a

Although we appreciate the visualizations of the design and outcomes, we found most of the figures not very clear and/or informative:

Figure 1B: It was not intuitive to us to visually understand the difference between high- and low-probability elements included in a triplet as shown in Figure 1B. Figure 1A and 1C serve, according to us, their purpose and provide a clear visualization of the (part of) the task in combination with the Figure's description and in-text explanation, but Figure 1B remains unclear.

Response #6a

We thank the Reviewer for pointing out the lack of clarity in the Figure 1 legend. We thoroughly revised both the main text and Figure 1 legend to improve clarity and completeness of the task description. We hope that these revisions make the relevance and interpretation of Figure 1B more transparent to the reader. Please see the revised manuscript for the relevant changes (pp. 5-7).

Comment #6b

Figure 3: When figure 3A is mentioned in the text for the first time (p. 10; line 352) it was not yet clear to us that smaller differences between high and low triplets indicate decreased statistical learning (and this also doesn't become very clear from figure 3A). This meant that the interpretation of Figure 3A only became clear to us when the Age*TripletType interaction is explained later in the Results section (which also relates to Figures 3C and 3D).

Response #6b

We thank the Reviewers for highlighting the lack of clarity in our figures and their accompanying explanations. We revised Figure 3 (pp. 14) to enhance interpretability and focus. The updated figure now includes only two panels: Figure 3A presents median RTs as a function of triplet type at each assessment point, illustrating triplet-level performance across development. Figure 3B shows the average learning score (that is, the raw RT difference between low- and high-probability triplets) at each assessment point, highlighting the age-related decline in statistical learning.

Comment #6cd

Figure 4B: The figure is a bit hard to interpret, and we found the in-text explanation clearer.

Figures 4CD: we do not really see how these figures support the outcome that better EF ability at age 14 is related to a decreasing trajectory of statistical learning between ages 7 and 14, given

that the individual scores/violin plots for the different subgroups overlap and thus suggest that there are no real differences between the different subgroups?

Response #6cd

We thank the Reviewers for their attention to the interpretation of Figure 4. We would like to clarify that the error bars in Figures 4C and 4D represent standard errors, not confidence intervals. Importantly, the statistical inferences in the main text are based on coefficients from the logistic regression predicting subgroup membership from both EF factor scores. Therefore, the error bars in the figure should not be interpreted as confidence intervals associated with the p-values reported in the main text. To improve clarity, we revised Figure 4 by removing panel B, and updated the figure legend to explicitly state that the figure presents descriptive statistics only, and that the logistic regression provides the relevant inferential test (pp. 16).

Comment #7

The authors may want to consider including the within-subject RTCV approach in the main paper instead of supplementary text. This approach addresses important issue in developmental psychology and they draw considerable conclusions from it in their Discussion.

Response #7

We appreciate the Reviewers' suggestion. During the revision process, we incorporated several control analyses directly relevant to our main research question, which we felt were important to present in the main manuscript. Given that these additions substantially increased the length of the Results section, we felt it was more appropriate to present the RTCV approach in the Supplementary Materials, along with information regarding RT transformations.

Comment #8

One of the conclusions is that there is a competitive relationship between statistical learning and cognitive processes (i.e., children with higher EF ability were more likely to show a decreasing profile of statistical learning and vice versa). We wonder how the authors would explain these findings within theories on a statistical/procedural learning deficit in children with DLD, a group of children who often showed reduced EF abilities as well, which seems to contradict the competition model?

Response #8

We thank the Reviewers for raising this important question. Indeed, at first glance, the procedural learning deficit hypothesis in developmental language disorder may appear to contradict the competition model. Nevertheless, we believe that these frameworks can coexist and serve different explanatory purposes.

The procedural learning deficit hypothesis aims to explain why a specific neurodevelopmental disorder occurs. In such conditions, both the development of cognitive functions and their interactions–as well as their underlying neural mechanisms–are atypical. Therefore, it would be inappropriate to expect that the competition model, which is intended to describe typical developmental trajectories, would also apply directly to atypical development.

Theoretical models of atypical development are essential and contrasting them with models of typical development can yield valuable insights. However, in our view, apparent contradictions between these models are more likely to reflect altered neurodevelopmental

processes in atypical development, rather than inconsistencies in our understanding of typical development.

Comment #9

Relatedly, the Discussion may benefit from a more elaborate reflection on what it may mean (in practice/real life) or theoretically that there are differ subgroups of statistical learners? What is the practical and/theoretical merit of this finding?

Response #9

We thank the Reviewers for their feedback, we extended our discussion on this topic (pp. 20).

Minor points

Comment #10

It would be helpful if the Introduction ends with an explicit outline of the research questions (as they are explicitly outlined in the Results section).

Response #10

We thank the Reviewers for their suggestion, now we explicitly outline our research questions in the revised Introduction (pp. 4).

Comment #11

At several points in the manuscript, the authors refer to 'internal models'. Not every reader (including us) may be familiar with this term. As such it would be helpful if the authors could elaborate a bit more on what they mean with internal models.

Response #11

We thank the Reviewers for bringing this to our attention. We chose to use the term 'internal models' to maintain consistency with the terminology used in the literature[3,4,11,27,28]. However, we recognize that a clear definition of the term was lacking. As a result, we have now revised the relevant section in the Introduction to improve clarity (pp. 3-4).

Comment #12

Page 5, line 155. What is the purpose/functioning of providing this type of feedback to participants?

Response #12

We provided participants with feedback on their average speed and accuracy to help maintain engagement and ensure sustained attention to the task.

Comment #13

What is the purpose/function of Table 1? The overall mean accuracy scores and response times are not very informative for this type of task, are they? Also, it is not entirely clear what the missing data represents (individual trials?)

Response #13

We thank you for the feedback. We included the descriptives for completeness and comparability with prior, cross-sectional studies. The missing data row indicated the number of participants with missing ASRT data at a given assessment point. We clarified this in the revised table.

Comment #14

Page 9: the authors describe how their effects are coded in the linear mixed models, but if we are correct, they do not explain their coding of Age

Response #14

We thank the Reviewers for pointing this out. The coding of Age in the linear mixed models are now included in the revised manuscript (pp. 9).

Comment #15

On page 11, the authors introduce the concept learning scores (high-low triplet type differences). Are these differences in raw response times or are these differences based on the estimates from the model outcomes?

Response #15

The learning scores are based on the estimates from the model outcomes. In the revised manuscript, we use the term *learning score contrast* to refer to performance differences between high- and low-probability triplets estimated from the LMM using the estimated marginal means approach, and the term *average learning score* to refer to performance differences between high- and low probability triplets calculated from raw data by averaging. This statement is now included in the revised manuscript (pp. 11).

Comment #16

We found two typos:

o ASRT sequence line 179

o Backward digit span task acronym line 211

Response #16

Thank you, we fixed the mentioned typos.

References

1. Juhasz, D., Nemeth, D. & Janacsek, K. Is there more room to improve? The lifespan trajectory of procedural learning and its relationship to the between- and within-group differences in average response times. *PLoS ONE* **14**, e0215116 (2019).
2. Bakdash, J. Z. & Marusich, L. R. Repeated Measures Correlation. *Front. Psychol.* **8**, 456 (2017).
3. Janacsek, K. & Nemeth, D. Procedural Memory: The Role of Competitive Neurocognitive Networks Across Development. in *The Cognitive Unconscious: The First Half Century* (eds. Reber, A. S. & Allen, R.) 22–36 (Oxford University Press, Oxford, 2022).
4. Nemeth, D., Janacsek, K., Polner, B. & Kovacs, Z. A. Boosting Human Learning by Hypnosis. *Cerebral Cortex* **23**, 801–805 (2013).
5. Wu, R., Rebok, G. W. & Lin, F. V. A Novel Theoretical Life Course Framework for Triggering Cognitive Development across the Lifespan. *Human Development* **59**, 342–365 (2016).
6. Gopnik, A. Childhood as a solution to explore–exploit tensions. *Phil. Trans. R. Soc. B* **375**, 20190502 (2020).
7. Song, S., Howard, J. H. & Howard, D. V. Implicit probabilistic sequence learning is independent of explicit awareness. *Learn. Mem.* **14**, 167–176 (2007).
8. Kóbor, A. *et al.* Tracking the implicit acquisition of nonadjacent transitional probabilities by ERPs. *Mem Cogn* **47**, 1546–1566 (2019).
9. Éltető, N., Nemeth, D., Janacsek, K. & Dayan, P. Tracking human skill learning with a hierarchical Bayesian sequence model. *PLoS Comput Biol* **18**, e1009866 (2022).
10. Szegedi-Hallgató, E., Janacsek, K. & Nemeth, D. Different levels of statistical learning-hidden potentials of sequence learning tasks. *PloS one* **14**, e0221966 (2019).
11. Janacsek, K., Fiser, J. & Nemeth, D. The best time to acquire new skills: age-related differences in implicit sequence learning across the human lifespan: Implicit learning across human lifespan. *Developmental Science* **15**, 496–505 (2012).
12. Lukács, Á., Lukics, K. S. & Dobó, D. Online statistical learning in developmental language disorder. *Frontiers in Human Neuroscience* **15**, 715818 (2021).
13. Siegelman, N., Bogaerts, L., Kronenfeld, O. & Frost, R. Redefining "learning" in statistical learning: What does an online measure reveal about the assimilation of visual regularities? *Cognitive science* **42**, 692–727 (2018).
14. Farkas, B. C., Krajcsi, A., Janacsek, K. & Nemeth, D. The complexity of measuring reliability in learning tasks: An illustration using the Alternating Serial Reaction Time Task. *Behav Res* (2023) doi:10.3758/s13428-022-02038-5.
15. Tóth-Fáber, E. *et al.* Dissociation within the procedural system in Tourette syndrome: Enhanced statistical and impaired sequence learning. *Manuscript submitted for publication.* (2019).
16. Janacsek, K., Borbély-Ipkovich, E., Nemeth, D. & Gonda, X. How can the depressed mind extract and remember predictive relationships of the environment? Evidence from implicit probabilistic sequence learning. *Progress in Neuro-Psychopharmacology and Biological Psychiatry* **81**, 17–24 (2018).
17. Kóbor, A. *et al.* ERPs differentiate the sensitivity to statistical probabilities and the learning of sequential structures during procedural learning. *Biological Psychology* **135**, 180–193 (2018).
18. Bogaerts, L., Siegelman, N. & Frost, R. Statistical Learning and Language Impairments: Toward More Precise Theoretical Accounts. *Perspect Psychol Sci* 174569162095308 (2020) doi:10.1177/1745691620953082.
19. Wechsler, D. & Kodama, H. *Wechsler Intelligence Scale for Children*. vol. 1 (Psychological corporation New York, 1949).

20. Tánczos, T., Janacsek, K. & Németh, D. Verbal fluency tasks I. Investigation of the Hungarian version of the letter fluency task between 5 and 89 years of age. *Psychiatria Hungarica: A Magyar Pszichiatriai Tarsasag tudomanyos folyoirata* **29**, 158–180 (2014).
21. Tánczos, T., Janacsek, K. & Németh, D. A verbális fluencia-tesztek II. A szemantikus fluencia-teszt magyar nyelvű vizsgálata 5-től 89 éves korig. *Psychiatria Hungarica* **29**, 181–207 (2014).
22. Case, R., Kurland, D. M. & Goldberg, J. Operational efficiency and the growth of short-term memory span. *Journal of Experimental Child Psychology* **33**, 386–404 (1982).
23. Mammarella, I. C., Pazzaglia, F. & Cornoldi, C. Evidence for different components in children's visuospatial working memory. *British journal of developmental psychology* **26**, 337–355 (2008).
24. Shao, Z., Janse, E., Visser, K. & Meyer, A. S. What do verbal fluency tasks measure? Predictors of verbal fluency performance in older adults. *Front. Psychol.* **5**, (2014).
25. Virag, M. *et al.* Competition between frontal lobe functions and implicit sequence learning: evidence from the long-term effects of alcohol. *Exp Brain Res* **233**, 2081–2089 (2015).
26. Pedraza, F. *et al.* Evidence for a competitive relationship between executive functions and statistical learning. *npj Sci. Learn.* **9**, 30 (2024).
27. Clark, A. Whatever next? Predictive brains, situated agents, and the future of cognitive science. *Behav Brain Sci* **36**, 181–204 (2013).
28. Wolpert, D. M., Ghahramani, Z. & Jordan, M. I. An internal model for sensorimotor integration. *Science* **269**, 1880–1882 (1995).

Addressing the issues highlighted in the Reviewers' comments

Reviewer #1

Comment #1

The authors have addressed many of my concerns, but I am still not sure how to interpret their link between the executive function data and the statistical learning data. In particular, the authors' control analyses found that there is no relationship between the statistical learning score at 14 years of age, and the EF measures. This seems to contradict the argument that executive functions and statistical learning processes are at odds with one another across development. The authors state that because the statistical learning scores at age 7 do not relate to scores on the EF tasks, nor do the statistical learning scores at age 14, the interpretation should be that the developmental trajectory is what underpins this link. Can the authors elaborate on what they mean by this in a more mechanistic way? If the argument is that overall statistical learning performance is irrelevant to EF task performance (but only the direction of change in the preceding 7 years is predictive), I'm not sure this supports the conclusion that these tasks are tapping underlying cognitive functions which are increasingly at odds with one another across development.

Response #1

We are grateful to the Reviewer for once again devoting their time and attention to our manuscript. We are pleased that most of our responses and clarifications were well received. We also acknowledge an important limitation of our dataset: executive functions were assessed only once, at age 14. This inevitably constrains our ability to disentangle the mechanisms underlying the observed associations. One possibility is that EF performance at age 14 serves as a proxy for earlier EF abilities, which may have been the true drivers of the relationship with SL trajectories. Alternatively, the EF score at age 14 may reflect the rate of EF development throughout childhood, such that individuals who matured more rapidly achieved higher scores by adolescence. This developmental pace could likewise explain the observed association.

When we argue that the developmental trajectory underpins the link between EF and SL, our central point is that cross-sectional correlations at a single time point miss the crucial dynamic patterns unfolding across development. For example, two individuals might show similar SL scores at age 14, yet follow very different developmental paths: one may have declined steadily from a higher baseline, while the other may have improved from a lower baseline. Such heterogeneity in learning trajectories likely obscures associations in cross-sectional analyses. Moreover, we do not necessarily expect the negative EF–SL correlations frequently reported in adulthood[1–3] to appear in childhood or adolescence. In adults, both systems underlying SL and EF are fully mature, enabling stable patterns of functional interaction. In contrast, the developmental timing of these systems is asynchronous in younger populations: SL typically reaches its peak level earlier in childhood, whereas EF continues to develop into late adolescence and young adulthood[4,5]. This developmental asynchrony may explain why cross-sectional associations emerge in adults but are absent or inconsistent in younger samples.

We believe that our findings align with a mechanistic account in which EFs at age 14 reflect the developmental culmination of a broader neurocognitive transition from input-driven learning to model-based control. During early and middle childhood, striatal systems that support input-driven learning, including SL, are dominant and function efficiently even in

infants and young children[4,6,7]. However, through adolescence, prefrontal–hippocampal circuits that support executive control and internal models continue to mature[5,8–10], gradually shifting learning dynamics from exploration of environmental input toward the strategic exploitation of prior knowledge[11–15]. As these prefrontal–hippocampal systems strengthen, they may gate, reinterpret, or down-weight environmental input, thereby reducing sensitivity to environmental patterns (e.g., statistical regularities) in favour of model-based (top-down) strategies. This shift is not necessarily suppressive in real time but reflects a restructuring of learning priorities: increasingly efficient model-based (top-down) processes begin to dominate behaviour, constraining the influence of input-driven (bottom-up) systems. EF components such as working memory and verbal fluency, often viewed as reflective of internal model access and control[16], thus act as indicators of the degree to which this transition has occurred. Consequently, adolescents with stronger EF at age 14, a proxy for more advanced prefrontal–hippocampal maturation and entrenched internal models, are precisely those who, over the preceding years, have experienced a steeper decline in SL performance. In contrast, adolescents with weaker EF may have undergone a slower transition to model-based control, preserving broader sensitivity to statistical input and resulting in flat or increasing SL trajectories. Importantly, this account explains why EF at age 14 predicts SL trajectory (i.e., class membership in latent class models) but not concurrent SL performance at age 14: EF is not acting as a real-time inhibitor of SL but as a developmental readout of cumulative neural and cognitive specialization. That is, the EF–SL relationship reflects a trajectory-dependent mechanism across development, not a cross-sectional trade-off.

Converging evidence supports this account. (1) Asynchronous maturation (earlier striatal vs. protracted hippocampal/PFC development) sets up changing system weights across childhood and adolescence[4,5,8–10]. (2) Competitive or interactive dynamics between striatal and hippocampal-prefrontal systems can suppress striatal learning when hippocampal-prefrontal control dominates[17]. (3) Entrenchment of prior knowledge and schemas with age can bias processing away from environmental regularities (statistics)[11,12,18]. (4) Finally, the well-documented developmental shift from exploration to exploitation reduces input-driven learning and promotes top-down, model-based control—core features of EF[13–15].

Taken together, this developmental account clarifies why EF at age 14 is associated with the **trajectory** of SL performance between ages 7 and 14, even in the absence of a concurrent EF–SL correlation. We now highlight this interpretation more explicitly in the revised manuscript (p. 19-20).

Comment #2

Second, I also did not understand the authors' decision to restrict their 2nd control analyses for my concern about ceiling effects to the second half of the experiment (although I appreciate the many other control analyses which point towards the same conclusion!). My original concern was that a learning signal measured as a difference in reaction time might be, by definition, lower if the reaction times are at ceiling in a group of participants. By restricting this control analysis to periods of time which have the most restricted range already, the authors might have been least likely to capture variability due to my original concern. Could the authors please more carefully explain why it makes sense to restrict the analyses to the period of time when this difference is the smallest?

Response #2

We are pleased that the Reviewer appreciates our extensive control analyses, and we thank them for pointing out that our rationale for focusing only on the second half of the task was not sufficiently explained. We acknowledge the concern that variability may be lower in the later blocks; however, our decision was motivated by the need to minimize confounds introduced by ongoing learning.

In the previous version of the manuscript, we noted that learning tends to stabilize in the second half of the task (Blocks 11-20). The correlation analysis restricted to these blocks therefore reduces the risk that the observed associations are driven by the learning effect itself. Specifically, the ASRT task involves two simultaneous processes: 1) participants respond increasingly faster to high-probability compared to low-probability triplets (reflecting statistical learning), and 2) they also become faster overall as the task progresses (general practice-related speed-up). These processes can produce a spurious negative correlation between learning scores and overall reaction times. By restricting the correlation analysis to the second half of the task, when most learning has already taken place, we aimed to limit this confounding effect.

Nevertheless, to address the Reviewer's concern, we additionally ran repeated-measures correlations for the entire task (all 20 blocks). For reference, the results based on the second half of the task (Blocks 11–20) were as follows:

Age 7: $r_{rm}(755) = 0.100$, 95% CI = [0.029, 0.170], $p = .006$, $BF_{01} = 0.25$

Age 8: $r_{rm}(547) = 0.006$, 95% CI = [-0.078, 0.090], $p = .890$, $BF_{01} = 10.00$

Age 11: $r_{rm}(600) = 0.014$, 95% CI = [-0.066, 0.093], $p = .738$, $BF_{01} = 10.00$

Age 14: $r_{rm}(895) = 0.030$, 95% CI = [-0.037, 0.097], $p = .377$, $BF_{01} = 8.33$

These results indicate a very weak positive correlation at age 7, where *faster* blocks were associated with slightly *lower* learning scores. At older ages, correlations were not significant and Bayes factors indicated moderate support for the null hypothesis. Overall, this suggests a weak relationship between baseline reaction times and learning scores at age 7, but not at later ages.

The analyses across all 20 blocks yielded the following results:

Age 7: $r_{rm}(1595) = 0.041$, 95% CI = [-0.008, 0.090], $p = .103$, $BF_{01} = 3.85$

Age 8: $r_{rm}(1155) = -0.079$, 95% CI = [-0.136, -0.021], $p = .007$, $BF_{01} = 0.50$

Age 11: $r_{rm}(1270) = -0.055$, 95% CI = [-0.110, 0.000], $p = .049$, $BF_{01} = 2.50$

Age 14: $r_{rm}(1819) = -0.079$, 95% CI = [-0.125, -0.034], $p < .001$, $BF_{01} = 0.07$

These analyses revealed very weak negative correlations at ages 8, 11, and 14, such that *faster* blocks were associated with slightly *higher* learning scores. This pattern is opposite to the Reviewer's concern and likely reflects the confounding influence of ongoing learning: as participants become faster on high-probability compared to the low-probability triplets (reflecting stronger learning), they also become faster overall, creating a spurious negative association between RTs and learning scores.

Taken together, these findings support our decision to restrict the within-participant correlation analyses to the second half of the task, where the confounding impact of the learning effect is reduced. Nonetheless, for completeness, we now report the whole-task results in the

Supplementary Materials (see Supplementary Text S5, pp. 15) and provide a more detailed explanation of our reasoning in the revised manuscript (pp. 19-20).

Reviewer #2 & Reviewer #3

Again, I co-reviewed this manuscript with one of the reviewers who provided the listed reports. This is part of the Nature Communications initiative to facilitate training in peer review and to provide appropriate recognition for Early Career Researchers who co-review manuscripts.

Dear authors, dear editor,

Overall the authors adequately addressed my comments (I was reviewer 2/3 in the previous round, and if they decided to not address my comment/suggestion in the revised manuscript they had good reasons to do so. Together with their changes in response to reviewer 1 I think the manuscript definitely improved and I would accept the current version with only two minor comments/suggestions:

Comment #1

1. There are a few null results in the new analyses that aim to test whether an overall decrease in RTs can explain the age-related changes. Please be careful in interpreting these null effects: in their discussion the authors now write that the observed decline in statistical learning cannot be explained by baseline RT confounds and instead reflects meaning age-related changes. This conclusion is formulated a bit too strong: one can only conclude from the null effect that there is no evidence that the observed decline in statistical learning can be explained by baseline RT confounds, which means something slightly different. Relatedly, the authors write in their Discussion that EF measures did not correlate with statistical learning at either age 7 or 14 and that this suggests that the observed relationship is specific to the developmental trajectory itself. A better formulation would be that they found no evidence that EF measures correlate with statistical learning at either age 7 or 14, which may suggest that […].

Response #1

We thank the Reviewers for carefully evaluating our manuscript once again and for their constructive and positive feedback. We also appreciate the Reviewers' comment regarding the interpretation of null effects. In response, we conducted Bayesian analyses for all correlational analyses to provide additional evidence regarding the null versus alternative hypotheses. Most Bayes factors indicated anecdotal to moderate support for the null hypothesis (pp. 15-17). Accordingly, we have moderated our conclusions and presented them more cautiously throughout the manuscript.

Comment #2

2. The authors did a good job in clarifying their Figures. However, I still had some difficulty understanding the difference between the low probability and high probability triplets (and Figure 1 did not immediately help). For me, it would help if the authors make explicit that in low probability trials (e.g., 2-X-1 and 2-X-2) the start and end trials are random and the X-element is part of the pattern (2, 4, 3, 1 in the concrete example from Figure 1), whereas in the high probability trials the start and end trial are part of the pattern and the X-element is random.

Response #2

We thank the Reviewers for this suggestion, we integrated it in the revised manuscript (pp. 6-7).

References

1. Pedraza, F. *et al.* Evidence for a competitive relationship between executive functions and statistical learning. *npj Sci. Learn.* **9**, 30 (2024).

2. Virag, M. *et al.* Competition between frontal lobe functions and implicit sequence learning: evidence from the long-term effects of alcohol. *Exp Brain Res* **233**, 2081–2089 (2015).

3. Ambrus, G. G. *et al.* When less is more: Enhanced statistical learning of non-adjacent dependencies after disruption of bilateral DLPFC. *Journal of Memory and Language* **114**, 104144 (2020).

4. Choi, E. J., Vandewouw, M. M., de Villa, K., Inoue, T. & Taylor, M. J. The development of functional connectivity within the dorsal striatum from early childhood to adulthood. *Developmental Cognitive Neuroscience* **61**, 101258 (2023).

5. Casey, B. J., Tottenham, N., Liston, C. & Durston, S. Imaging the developing brain: what have we learned about cognitive development? *Trends in cognitive sciences* **9**, 104–110 (2005).

6. Saffran, J. R., Aslin, R. N. & Newport, E. L. Statistical Learning by 8-Month-Old Infants. *Science* **274**, 1926–1928 (1996).

7. Janacsek, K., Fiser, J. & Nemeth, D. The best time to acquire new skills: age-related differences in implicit sequence learning across the human lifespan: Implicit learning across human lifespan. *Developmental Science* **15**, 496–505 (2012).

8. Tervo-Clemmens, B. *et al.* A canonical trajectory of executive function maturation from adolescence to adulthood. *Nat Commun* **14**, 6922 (2023).

9. Gogtay, N. *et al.* Dynamic mapping of human cortical development during childhood through early adulthood. *Proc. Natl. Acad. Sci. U.S.A.* **101**, 8174–8179 (2004).

10. Gogtay, N. *et al.* Dynamic mapping of normal human hippocampal development. *Hippocampus* **16**, 664–672 (2006).

11. Wu, R., Rebok, G. W. & Lin, F. V. A Novel Theoretical Life Course Framework for Triggering Cognitive Development across the Lifespan. *Human Development* **59**, 342–365 (2016).

12. Seitz, A. R. Perceptual Learning: Changes across the Lifespan. *Current Biology* **31**, R69–R72 (2021).

13. Gopnik, A. *et al.* Changes in cognitive flexibility and hypothesis search across human life history from childhood to adolescence to adulthood. *Proceedings of the National Academy of Sciences* **114**, 7892–7899 (2017).

14. Frankenhuis, W. E. & Gopnik, A. Early adversity and the development of explore–exploit tradeoffs. *Trends in Cognitive Sciences* S1364661323000918 (2023) doi:10.1016/j.tics.2023.04.001.

15. Gopnik, A. Childhood as a solution to explore–exploit tensions. *Phil. Trans. R. Soc. B* **375**, 20190502 (2020).

16. Unsworth, N., Brewer, G. A. & Spillers, G. J. Working memory capacity and retrieval from long-term memory: the role of controlled search. *Mem Cogn* **41**, 242–254 (2013).

17. Poldrack, R. A. & Packard, M. G. Competition among multiple memory systems: converging evidence from animal and human brain studies. *Neuropsychologia* **41**, 245–251 (2003).

18. Forest, T. A., Schlichting, M. L., Duncan, K. D. & Finn, A. S. Changes in statistical learning across development. *Nat Rev Psychol* **2**, 205–219 (2023).

Addressing the issues highlighted in the Reviewers' comments

Reviewer #1

Comment #1

Dear editors and authors,

I re-read the manuscript and read the authors' response to my concerns about claiming a trade-off or support for a competitive model between statistical learning and executive functions across development. While I agree that the trajectory analyses are interesting and worth reporting, I still find the that claim that this means statistical learning and executive functions are competitive processes, or at odds with one another, across development is not well supported.

A primary concern is that the manuscript and rebuttal present a somewhat misleading account of the neurocognitive mechanisms underlying statistical learning. The text implies that statistical learning is uniquely supported by striatal processes, whereas hippocampal and prefrontal regions are not involved. For example, the authors state, "During early and middle childhood, striatal systems that support input-driven learning, including SL, are dominant and function efficiently even in infants and young children3,11,81." However, these citations do not demonstrate that striatal systems support statistical learning, nor uniquely: one paper shows that infants can perform well on statistical learning tasks, another reports developmental changes in striatal connectivity during adolescence, and a third shows reaction time changes across childhood in statistical learning tasks—but none of these report any evidence linking striatal function to statistical learning. While there is evidence that the basal ganglia are involved in statistical learning in adults and children (e.g. Mcnealy et al., 2010; Turk-Browne et al., 2009), there is much evidence that the hippocampus and prefrontal cortex also contribute to statistical learning in both adults (see, for instance: Schapiro et al., 2016; Schapiro et al., 2017; Turk-Browne et al., 2009; Covington et al., 2018; Schapiro et al., 2014) and young learners (Ellis et al., 2021; Finn et al., 2019; Schlichting et al. 2017). Thus, building a theoretical argument based on striatal and hippocampal/prefrontal development to describe potential trade-offs between statistical learning and executive functions in the developing brain (especially without neural evidence) is premature. It is, of course, possible that these systems contribute differently across development and that the developmental trade-offs you suggest do exist, but the current evidence does not firmly support this claim. I leave the final decision to the editor, but I recommend that the authors either remove these claims, or frame their mechanistic claims much more cautiously throughout the manuscript (for instance, the final paragraph still includes this claim), with more careful citations, acknowledging both the broader neural contributions and the speculative nature of developmental interactions.

Response #1

We appreciate the time and energy the Reviewer spent re-evaluating our manuscript. We understand the Reviewer concerns that the theoretical framework is premature. We removed the paragraph describing the mechanistic account and now interpret our results much more cautiously in the revised manuscript (pp. 20-21).

Reviewer #2 & #3

Comment #1

Dear authors,

Thank you for your resubmission. You've addressed the comments of my previous revision and I look forward seeing your manuscript published.

Response #1

We greatly appreciate the Reviewers feedback.

<u>Reviewer #4</u>

Comment #1

I was asked to review the LCA methodology.

Whilst it is commendable that authors applies this method, the fit statistics strongly indicated that the two-class solution provided the most appropriate balance of model fit, parsimony, and classification quality. Both AIC and SABIC favoured the two-class model (lower values indicating better fit). Entropy for the three-class solution also fell to 0.65 from 0.95, indicating poor class separation and uncertainty in individual class assignments. Entropy < 0.70 is generally considered unacceptable for LCA as it indicates that the classes overlap too much. Class sizes further supported this: both the two- and three-class models generated a very small class containing only 3 individuals, suggesting this subgroup reflected statistical noise/outliers rather than a substantively meaningful latent class. In contrast, the remaining classes in the three-class model were split into two larger groups without any corresponding improvement in fit, implying over-extraction or an artefact of the method used. The authors do not specify if they considered specifying time as non-linear (e.g, using polynomial or spline functions). Furthermore, a different clustering algorithm could yield different results. The authors have not provided sufficient justification for retaining the third class.

Response #1

We appreciate the time the Reviewer spent evaluating our manuscript. First, we would like to respectfully note that while entropy is informative about classification certainty, our reading of the LCA/LGMM literature suggests that it is not recommended for determining the number of latent classes (Ram & Grimm, 2009; Tein et al., 2013; van de Schoot et al., 2017). Nevertheless, we fully agree that in light of the modest entropy, the robustness and validity of the "increase" and "decrease" classes could be more thoroughly investigated. To this end, we fit four additional longitudinal clustering models spanning distinct algorithmic families. Across all alternative methods, SABIC favored the 3-class solution, with qualitatively similar trajectory patterns as our original model (see Supplementary Figure S7), with moderate to perfect agreement between algorithms (Adjusted Rand Index ≈ 0.48–1.00; Normalized Mutual Information ≈ 0.55–1.00). In addition, depending on the sample sizes of the classes, we estimated multinomial or binary logistic regression models relating working memory and fluency to the probability of belonging to the "increase" versus other classes for each clustering algorithm. Across all methods, higher executive function was associated with increased odds of belonging to the decreasing trajectory, with odds ratios of comparable magnitude across models, although statistical significance dependent on the model (EF WM ≈ 1.58 - 2.76; EF Fluency ≈ 1.52 - 2.03; see Supplementary Figure S8). We believe that these additional analyses provide strong support for the 3-class solution and demonstrate that the association between executive functions and class membership is robust, further validating our approach.

Comment #2

In Figure 4, the CIs cross, indicating no difference in scores.

Response #2

This description does not accurately reflect Figure 4 in the submitted manuscript. The figure illustrates class membership prediction from executive function measures, and the error bars represent ±1 SEM (as specified in the figure legend, p. 16), not confidence intervals. The conclusion of "no difference" thus appears to result from a misinterpretation of the error bars.

Comment #3

For the "Increase" class, the authors mention that there is a "slight increase in average learning scores with development". Is this an appropriate label? How much of an increase would be considered meaningful (e.g., could this be judged on standard deviation increase between the first and last time-points?). What about those who have a stable trajectory? I believe more exploratory work should be undertaken to understand if these classes are real.

Response #3

The class labels were not assigned based on visual inspection alone, but follow directly from the estimated intercept and slope parameters of the growth mixture model. The "increase" class thus reflects a significantly positive slope, the "decrease class" a significantly negative slope, and the "high start, steep decrease" class a large significantly negative slope and a large significantly positive intercept. A "stable" trajectory would correspond to a non-significant slope; however, no such distinct class emerged in the selected model solution. To improve clarity, we have now added the model-implied difference between the first and last time points for each class, expressed both in milliseconds (RT) and in standardized units (SD change), as suggested. We have clarified these points in the relevant section of the revised manuscript (pp. 15).

Comment #4

Furthermore, because one of the latent classes contained only three individuals, I don't believe it is methodologically appropriate to estimate predictors of class membership using multinomial regression. Multinomial models require sufficient observations within each outcome category to obtain stable parameter estimates, and extremely small classes produce unreliable or non-estimable coefficients, and large standard errors. In this case, the tiny class size, combined with the poor entropy observed in the three-class solution, indicates that the class is statistically unstable and unlikely to represent a meaningful underlying subgroup. Running a multinomial regression would, therefore, yield results that are neither robust nor interpretable. It was difficult to judge the results of the multinomial regression as no regression table was provided and it wasn't clear what the reference category was.

Response #4

We agree that applying multinomial regression to a latent class comprising only three individuals would be methodologically inappropriate. However, multinomial regression was not used. Instead, logistic regression was conducted on the two largest classes to avoid precisely these issues (see pp. 15–16 in the manuscript). We explicitly state in the manuscript that the class containing only three individuals was excluded from the regression analysis: *"Due to the very low number of participants, we omitted the 'high start steep decrease' class from this analysis."* (pp. 15). Moreover, we also state that „*the reference class was set to 'decrease', as it includes the largest number of participants*" in the manuscript (pp. 15).

To further clarify, we added a regression table summarizing these results (see Table 5 in the revised manuscript). Since for the two alternative models with a large enough third class (LMKM, GCKM) it was more intuitive to compare the two decreasing subgroups to the 'increase' group as the reference. For consistency, we also changed to this model specification in the main text and reworked the relevant sections accordingly (pp. 16). Hence, in the revised manuscript, the 'increase' group is used as reference for each regression analyses. We also changed the ordering of classes in Figure 4, so that the reference category of 'increase' appears first in each subpanel, further reinforcing this. For the sensitivity analyses detailed above, we did the same.
During revision, we also updated the weighting procedure used in these analyses to align more closely with the recommended approach of using posterior probabilities directly, rather than logit-transformed posterior probabilities (Clark & Muthén, 2009). The revised analyses led to the same substantive conclusion: across all models, the joint LRT continued to indicate a significant effect of EF factor scores, with higher EF scores associated with increased odds of belonging to the 'decrease' subgroup relative to the 'increase' subgroup. Although the relative strength of the Fluency and WM scores varied across modelling approaches, the overall EF-related pattern and its interpretation remained unchanged.

Works cited

Clark, S. L., & Muthén, B. O. (2009). *Relating latent class analysis results to variables not included in the analysis*. http://www.statmodel.com/download/relatinglca.pdf

Ram, N., & Grimm, K. J. (2009). Methods and Measures: Growth mixture modeling: A method for identifying differences in longitudinal change among unobserved groups. *International Journal of Behavioral Development*, *33*(6), 565–576. https://doi.org/10.1177/0165025409343765

Tein, J.-Y., Coxe, S., & Cham, H. (2013). Statistical Power to Detect the Correct Number of Classes in Latent Profile Analysis. *Structural Equation Modeling: A Multidisciplinary Journal*, *20*(4), 640–657. https://doi.org/10.1080/10705511.2013.824781

van de Schoot, R., Sijbrandij, M., Winter, S. D., Depaoli, S., & Vermunt, J. K. (2017). The GRoLTS-Checklist: Guidelines for Reporting on Latent Trajectory Studies. *Structural Equation Modeling: A Multidisciplinary Journal*, *24*(3), 451–467. https://doi.org/10.1080/10705511.2016.1247646
